# How to protect long-term care facilities from pandemic-like events? - A systematic review on the effectiveness of non-pharmacological measures to prevent viral respiratory infections

**DOI:** 10.1186/s12879-024-09271-7

**Published:** 2024-06-17

**Authors:** Laura Arnold, Simon Bimczok, Hannah Schütt, Stefanie Lisak-Wahl, Barbara Buchberger, Jan M Stratil

**Affiliations:** 1 Academy of Public Health Services, Kanzlerstraße 4, Duesseldorf, 40472 Germany; 2 Department of International Health, Care and Public Health Research Institute—CAPHRI, Faculty of Health, Medicine and Life Sciences, Maastricht University, Maastricht, The Netherlands; 3 Robert Koch Institute, Berlin, Germany; 4 University of Duisburg-Essen, Institute for Health Care Management and Research, Essen, Germany; 5 Department of Infectious Disease Epidemiology, Robert Koch Institute, Berlin, Germany; 6 Postgraduate Training for Applied Epidemiology (PAE), Robert Koch Institute, Berlin, Germany; 7 Field Epidemiology Path (EPIET), European Centre for Disease Prevention and Control (ECDC), ECDC Fellowship Programme, Stockholm, Sweden

**Keywords:** Nursing homes, Pandemics, COVID-19, Influenza, Respiratory tract infections, Public health practice, Communicable disease control, Physical distancing, Mandatory testing, Hygiene

## Abstract

**Background:**

The SARS-CoV-2 pandemic underscored the need for pandemic preparedness, with respiratory-transmitted viruses considered as a substantial risk. In pandemics, long‐term care facilities (LTCFs) are a high-risk setting with severe outbreaks and burden of disease. Non‐pharmacological interventions (NPIs) constitute the primary defence mechanism when pharmacological interventions are not available. However, evidence on the effectiveness of NPIs implemented in LTCFs remains unclear.

**Methods:**

We conducted a systematic review assessing the effectiveness of NPIs implemented in LTCFs to protect residents and staff from viral respiratory pathogens with pandemic potential. We searched Medline, Embase, CINAHL, and two COVID-19 registries in 09/2022. Screening and data extraction was conducted independently by two experienced researchers. We included randomized controlled trials and non-randomized observational studies of intervention effects. Quality appraisal was conducted using ROBINS-I and RoB2. Primary outcomes encompassed number of outbreaks, infections, hospitalizations, and deaths. We synthesized findings narratively, focusing on the direction of effect. Certainty of evidence (CoE) was assessed using GRADE.

**Results:**

We analysed 13 observational studies and three (cluster) randomized controlled trials. All studies were conducted in high-income countries, all but three focused on SARS-CoV-2 with the rest focusing on influenza or upper-respiratory tract infections. The evidence indicates that a combination of different measures and hand hygiene interventions can be effective in protecting residents and staff from infection-related outcomes (moderate CoE). Self-confinement of staff with residents, compartmentalization of staff in the LTCF, and the routine testing of residents and/or staff in LTCFs, among others, may be effective (low CoE). Other measures, such as restricting shared spaces, serving meals in room, cohorting infected and non-infected residents may be effective (very low CoE). An evidence gap map highlights the lack of evidence on important interventions, encompassing visiting restrictions, pre-entry testing, and air filtration systems.

**Conclusions:**

Although CoE of interventions was low or very low for most outcomes, the implementation of NPIs identified as potentially effective in this review often constitutes the sole viable option, particularly prior to the availability of vaccinations. Our evidence-gap map underscores the imperative for further research on several interventions. These gaps need to be addressed to prepare LTCFs for future pandemics.

**Trial registration:**

CRD42022344149.

**Supplementary Information:**

The online version contains supplementary material available at 10.1186/s12879-024-09271-7.

## Background

The SARS-CoV-2 pandemic is estimated to have taken the lives of 18.2 million people worldwide between January 2020 and December 2021 [1], prior to the widespread availability of effective vaccines. However, it is just one of several major epidemics and pandemics that have occurred in the past century, including the 2003 SARS epidemic, the 2012 MERS epidemic, the H1N1 influenza pandemics in 2009 and 2018, and the 1957 H2N2 and 1968 influenza pandemics. As similar epidemic and pandemics are to be expected, preparation is essential.

According to the Johns Hopkins Center for Health Security, RNA viruses with respiratory transmission, particularly those disseminated through airborne and respiratory droplet routes, represent the most probable aetiological agents for initiating pandemics [2]. These included for example orthomyxoviruses, paramyxoviruses, pneumoviruses, coronaviruses, and some picornaviruses [3]. Moreover, non-epidemic acute respiratory tract infections (RTIs) resulting from viral pathogens, such as seasonal influenza, place a considerable burden on population health and healthcare systems around the world [4]. Beyond their immediate repercussions, viral-induced acute RTIs can lead to secondary bacterial lower RTIs, notably pneumococcal pneumonia, which engender elevated mortality rates, particularly within vulnerable demographic cohorts, such as the elderly.

During epidemic or pandemic events, individuals residing in long-term care facilities (LTCFs) are confronted with an elevated susceptibility. These residents often necessitate intimate care interactions involving multiple disciplines and healthcare providers. This circumstance constrains the feasibility of implementing social distancing measures and concurrently heightens the risk for infections and outbreaks within LTCFs [5, 6]. In case of an outbreak, the underlying conditions requiring individuals to receive care in the first place also increases their risk of severe courses of disease in case of infection, e.g. in the case of COVID-19 or influenza [7]. This amalgamation of factors has positioned LTCFs as focal points for the morbidity and mortality burdens associated with the SARS-CoV-2 pandemic [8] and other outbreaks precipitated by respiratory infections [9]. Notably, during the initial wave of the SARS-CoV-2 pandemic in Europe, LTCF residents accounted for a substantial proportion of fatalities, ranging from 26 and 66% deaths across 11 European countries [10]. Another analysis encompassing 21 high- and middle-income countries during the early phase of the pandemic attributed 46% of deaths to LTCF residents, although they constitute less than 1% of the population [8].

In the context of an epidemic or pandemic event, particular during the early phases when effective vaccines or treatments are scarce or non-existent, non-pharmacological interventions (NPIs) constitute the primary and often sole defence against infectious pathogens [11–13]. NPIs have several potential advantages over pharmacological interventions (PIs) of suppressing outbreaks within LTCFs. They can be swiftly implemented, are often less resource- and technology-dependent, and exhibit effectiveness across a spectrum of infectious agents, including novel viruses [11]. Even under conditions where pharmaceutical agents or vaccines are available, as exemplified in the case of influenza, reliance solely on PIs may not be sufficient to contain outbreaks in LTCFs or sufficiently ameliorating their adverse health consequences [14–16]. Consequently, the World Health Organization (WHO) has emphasized the importance of NPIs in its recent Global Influenza Strategy 2019 to 2030 [11] and other guidance documents on the management of the SARS-CoV-2 pandemic within LTCFs [6].

**Fig. 1:**
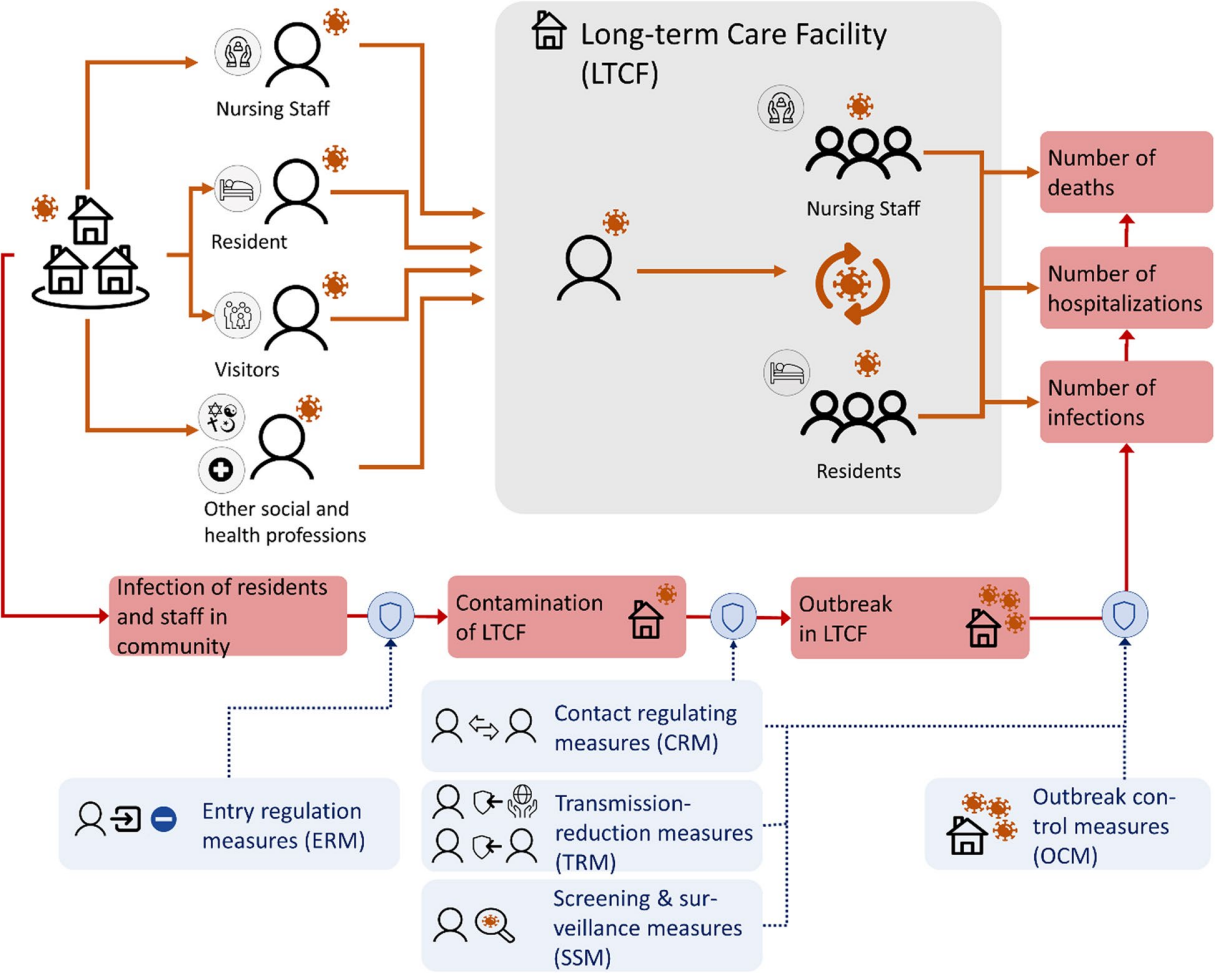
Process-based logic model on the relation between non-pharmaceutical intervention domains and potential outcome measures

Amidst the backdrop of the SARS-CoV-2 pandemic, NPIs implemented within LTCFs encompassed a range of measures, as depicted in the process-based logic model in Fig. 1. These included, but were not limited to, measures designed to restrict the introduction of the pathogen into the facility, such as entry regulation measures like visitor restrictions, contact-reduction measures involving for example the compartmentalization of residents, transmission-reducing measures like the utilization of masks and heightens surface hygiene, surveillance and testing initiatives, and measures directed at controlling outbreaks, including contact tracing, quarantine, and isolation [17]. Nonetheless, considerable uncertainties remain regarding the effectiveness of these and other NPIs to prevent or mitigate outbreaks due to SARS-CoV-2, as well as other viral RTIs aside from SARS-CoV-2.

### Objectives

The primary aim of this review was to provide evidence on the effectiveness of NPIs designed to prevent outbreaks within LTCFs and mitigate their infection-related consequences. Specifically, we assessed the impact of these interventions on the incidence of infections, hospitalizations, and mortality rates among both LTCF residents and staff, focusing on viral RTIs as a proxy for pathogens with pandemic potential. Additionally, we enhanced the applicability of our findings by consolidating the evidence related to these infection-related outcomes into one composite outcome, reflecting the potential effectiveness of the measure as a safeguarding strategy for LTCFs in forthcoming pandemics.

## Methods

In this review, we conducted an update of the Cochrane review *Non-pharmacological measures implemented in the setting of long-term care facilities to prevent SARS-CoV-2 infections and their consequences: a rapid review* (from here on: *Cochrane SARS-CoV-2 LTCF review*) [17] up to September 2022 as well as expanded the focus to include infections due to other viral pathogens with pandemic potential. In doing so, we partly draw on the methods of the Cochrane SARS-CoV-2 LTCF review as well as the Cochrane review *Physical interventions to interrupt or reduce the spread of respiratory viruses* by Jefferson et al. [18].

This review was prospectively registered in PROSPERO (CRD42022344149). The research process followed the recommendations outlined in the Cochrane Handbook for Systematic Reviews of Interventions. The Preferred Reporting Items for Systematic reviews and Meta-Analyses (PRISMA) statement, updated in 2020, was used to report this study [19].

### Criteria for including studies

We included both (cluster) randomized controlled trials (RCTs/cRCTs) as well as non-randomized observational studies of intervention effects (NRSIs) [20, 21]. We included NRSIs which allow control of observed and unobserved confounding, such as controlled before-and-after (CBA) studies, interrupted-time-series (ITS) studies, or regression discontinuity (RD) studies [21]. We furthermore included controlled prospective and retrospective cohort studies (PCS/RCS), where it was established that the intervention was introduced prior to the occurrence of the outcome. Mathematical modelling studies were excluded.

Studies that assessed at least one measure subsumed in one of the following intervention categories were included:

(i) Entry regulation measures (ERM) intended to limit the introduction of the pathogen into the LTCF (e.g., access restrictions for visitors),
(ii) Contact regulating measures (CRM) intended to reduce the number of contacts at risk of transmission within the facility (e.g., closing communal spaces),
(iii) Transmission-reduction measures (TRM) intended to reduce the risk of transmission upon contact or via surfaces (e.g., use of respiratory protective equipment, improving hand hygiene, or installing air filters),
(iv) Screening and surveillance measures (SSM) intended to detect cases early or identify asymptomatic but contagious individuals, and
(v) Outbreak control measures (OCM) implemented to interrupt or prevent further spread of an outbreak after a case of the respective disease detected (e.g., contact-tracing and quarantine).

Our review exclusively focused on NPIs implemented within the setting of LTCFs, defined as residential establishments providing care for people requiring support due to difficulties in maintaining independent living within the community. In this review, the term “LTCF” encompasses various types of facilities, including skilled nursing facilities, nursing homes, retirement homes, assisted-living facilities, residential care homes, and similar institutions [10]. We included studies that assessed the outcomes among three distinct groups: adult residents (≥ 18 years) primarily residing in LTCFs (referred to as residents), individuals visiting LTCFs (referred to as visitors), and nursing and non-nursing staff employed in LTCFs (referred to as staff). Furthermore, our inclusion criteria encompassed studies examining the impact of NPIs by means of comparative analyses, juxtaposing them against either the absence of any intervention (referred to as no measure), baseline infection control measures exclusively, less stringent implementation, or alternative NPIs.

We encompassed studies assessing infection-related outcomes in LTCFs or the population of interest. These outcomes included the number, rate, or proportion of

(i) viral respiratory infections (both confirmed or suspected),
(ii) contaminations of LTCFs (defined as at least one infection that was introduced into the LTCF),
(iii) outbreaks in LTCFs (defined as more than one infection in the facility or one case where the infection had occurred within the LTCF),
(iv) hospitalizations due to the pathogens of interest and
(v) deaths due to the pathogen of interest.

We included studies that assessed these outcomes in the context of a pandemic or epidemic event of a viral, respiratory-transmitted pathogen. Viruses of interest included, but were not limited to, Influenza, SARS-CoV-2, SARS, MERS. Additionally, we considered studies evaluating the effectiveness of NPIs against infection-related outcomes caused by similar viral pathogens which are transmitted via the respiratory route, such as influenza-like illnesses in general or acute upper respiratory tract infections (e.g., influenza, RSV-infections, rhinovirus-infections). See Supplementary files for details.

We considered studies published in English, French, German, Italian, and Spanish and excluded studies in languages other than those listed. In order to reflect changes in (medical) care and public health practice but to include publications from the 2003 SARS-pandemic, we restricted searches to the past 30 years (i.e., in or before 1992). The search covered the period up to September 2022, as this was the month the searches were conducted.

The eligibility criteria are provided in more details in Supplement Ib.

### Identification of relevant literature

Our database searches consisted of two components: The first component, an update of the database searches of the Cochrane SARS-CoV-2 LTCF review [17], focused on SARS-CoV-2 literature and was conducted in the two SARS-CoV-2 specific study registries *Cochrane COVID-19 Register* and *WHO COVID-19 Global literature on coronavirus disease*, which collectively encompass various databases. Additionally, we searched the CINAHL EBSCO database. The second component focused on literature searches on other pathogens of interest, conducted within the databases Embase (Ovid), MEDLINE (Ovid), and CINAHL EBSCO. The search strategy is provided in Supplement II. To identify relevant literature, we conducted backward citation searches via Scopus for all known literature reviews (provided in Supplement III), guidelines and all included studies.

Following Cochrane guidance [22], initial screening of titles and abstracts was carried out independently and in duplicate by two review authors, guided by pre-specified eligibility criteria (Supplement Ib) using the web-based application Rayyan [23]. Subsequently, full-text screening was similarly conducted in duplicate and independently. In both stages, discrepancies were resolved through discussion in the presence of an additional reviewer.

### Data collection, extraction, and assessment

Data extraction was performed by one author using a pre-developed and validated data extraction form and checked by one additional review author. For the risk of bias (RoB) assessment, two review authors independently employed specific tools tailored to the study type. RCTs were assessed using the Cochrane RoB 2 tool [24] with adapted versions for cluster-RCTs [25]. NRSIs were assessed using the ROBINS-I tool [26], employing guidance on the adaption of the tool laid out in the Cochrane Handbook for the RoB assessment of CBA and (controlled) ITS studies [27]. NRSI which were evaluated with ROBINS-I and judged to have a critical risk of bias rating were excluded from the evidence synthesis.

Our primary focus during the RoB assessment laid on assessing the potential influence of bias in the reported direction of effect. Specifically, the likelihood that bias may have contributed to the observed effect direction, as opposed to the true effect being either null or in the opposite direction.

An extended description of the risk of bias assessment is provided in Supplement IV.

### Data synthesis

For the data synthesis, we initially planned a meta-analysis to pool intervention effects within the same domain and category, contingent on the availability of a minimum of three relevant studies and data compatibility [28]. However, since there were fewer than three studies reporting the same outcome measure for all comparisons, we resorted to a narrative synthesis through using vote counting based on the direction of effect and visualization through effect-direction plots [29, 30], aligning with guidance from the Cochrane Handbook and reporting guideline on Synthesis without meta-analysis (SWiM) [28, 31].

The threshold for the public health relevance was defined as any difference from the null, regardless of statistical significance. We assumed that in an ongoing pandemic, any intervention which allows for a reduction of infection risk could potentially be relevant. Accordingly, the focus of the evidence appraisal and synthesis lay on the direction of effect, rather than the effect size.

Our synthesis approach involved a vote counting to determine effect directions, culminating in composite outcome measures that encapsulated intervention effectiveness in safeguarding LTCFs against infection-related endpoints. Within this composite outcome, we synthesized the direction of effect for the different infection-related outcome measures. In this synthesis, each study contributed one single effect estimate per comparison. In cases where a study addressed multiple infection-related endpoints (e.g., the study reporting both on number of infections and number of hospitalizations), priority was given to outcomes in the following order: Number, rate, or proportion of (1) outbreaks or (2) LTCF contaminations, followed by (3) infections, (4) hospital admission and (5) deaths resulting from infections caused by the pathogens of interest.

Our main interest was in assessing the components of interventions intended to prevent or reduce infection-related outcome, focusing on the effectiveness of adherence to these components, such as mask usage. Interventions intended to implement or increase effective components, like educative initiatives promoting mask wearing, were not within our scope.

More details on our approach is provided in Supplement IV.

### Assessment of certainty of evidence

We used the GRADE framework to assess the certainty of evidence regarding the primary outcomes [32, 33]. Initially, one review author compiled the evidence in summary of findings tables and established an initial certainty of evidence assessment, which was subsequently refined though deliberations within the research team. Finally, all results were verified by the research team [34].

## Results

### Results of the search

**Fig. 2:**
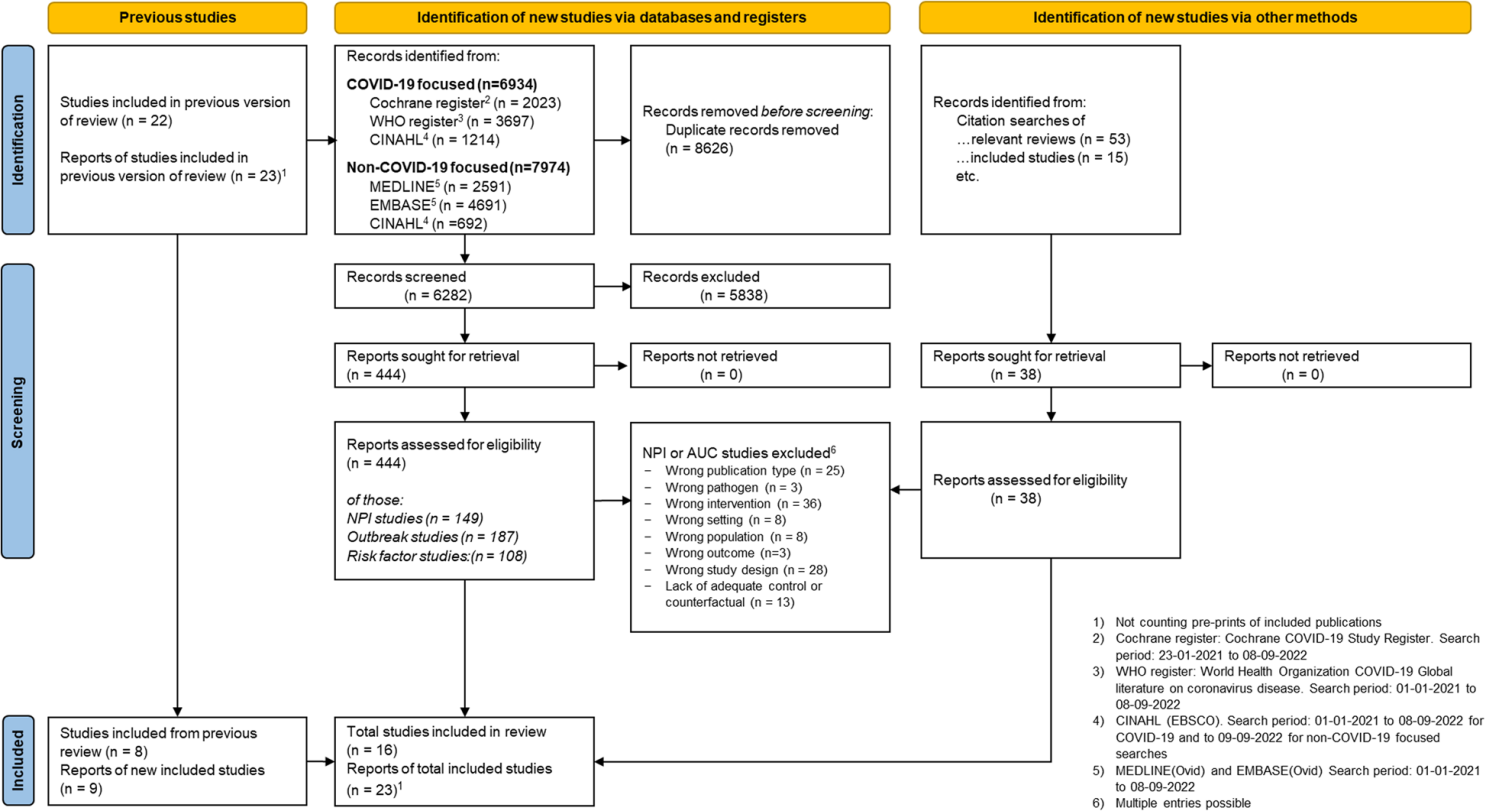
PRISMA 2020 flow diagram for new systematic reviews. (Abbreviations: *NPI* Non-pharmacological intervention, *AUC* Adverse and other unintended outcomes)

The database searches focused on SARS-CoV-2 spanned from January 2021 to September 2022, as the period before January 2021 was addressed in the Cochrane SARS-CoV-2 LTCF review [17], resulting in initial 6,934 records prior to deduplication. Additionally, 22 studies with 23 records from the previous Cochrane SARS-CoV-2 LTCF review were included in the full-text screening stage. The second component, focusing on pathogens other than SARS-CoV-2, covered the period up to September 2022 and yielded 7,974 records prior to deduplication. During full-text screening, 482 unique records were assessed. Overall, we included 23 records reporting on unique 16 studies [35–57]. Refer to Fig. 2 for more details. In our searches, we also included NRSIs investigating the adverse and unintended consequences of NPIs on resident’s and staff’s mental and physical health, which will be addressed in a separate publication. Regarding this outcome, we identified an additional two studies [58, 59].

### Description of included studies

As shown in Table 1, entry regulation measures (ERM) were assessed in one study [36], contact-regulating measures (CRM) in three [39, 42, 49] and transmission-reduction measures (TRM) in six [43, 45, 46, 48, 49, 54]. Five studies assessed screening and surveillance measures (SSM) [35, 41, 50, 55, 56] and two studies outbreak control measures (OCM) [45, 48]. Additionally, three studies examined the effects of multi-component measures (MCM) [44, 45, 57].

**Table 1:** Characteristics of the included studies

Study	Pathogen or disease	Location	Observational period	Population	Study Type	Intervention domain	Intervention category	Outcome category
Allan-Blitz 2022 [35]	SARS-CoV-2	USA, Florida	07/2020 to 04/2021	361 LTCFs	ITS/RMS	SSM	Routine testing of residents and/or staff	INF/OBS
Belmin 2020 [36–38]	SARS-CoV-2	France	03/2020 to 05/2020	17 LTCFs with 1250 residents	CCS	ERM	Self-confinement of staff in LTCFs	OBS/CONT; INF/OBS; HOSP/DEATH
Corvol 2022 [39]	SARS-CoV-2	France	03/2020 to 05/2020	231 LTCFs with 20,881 residents	CCS	CRM	Restrictions in the use of shared spaces	OBS/CONT; HOSP/DEATH
Ehrlich 2021 [41, 47]	SARS-CoV-2	USA	04/2020 to 08/2020	34 LTCFs with an average of 135 beds	ITS/RMS	SSM	Routine testing of residents and/or staff	INF/OBS
Green 2021 [42]	SARS-CoV-2	United Kingdom	04/2020 to 05/2020	34 LTCFs with 856 residents	CCS	CRM	Restrictions in the use of shared spaces	OBS/CONT
Ho 2012 [43]	respiratory outbreaks	China, Hong-Kong	11/2009 to 07/2010	18 LTCFs with 2407 residents and 810 staff members	cRCT	TRM	Hand hygiene	OBS/CONT
Lipsitz 2020 [45]	SARS-CoV-2	USA	04/2020 to 05/2020	360 LTCFs	CCS	TRM; OCM; MCM	Mask use and use of personal protective equipment (PPE)	INF/OBS; HOSP/DEATH
Lipsitz 2022 [44]	SARS-CoV-2	USA	04/2020 to 08/2020	65 LTCFs with 6578 residents	CBA	MCM	Combination of multicomponent measures	INF/OBS
Makris 2000 [46]	upper RTI	USA	not reported	8 LTCFs with 890 beds	cRCT	TRM	Hand hygiene	INF/OBS
Reyné 2020 [48]	SARS-CoV-2	France	03/2020 to 05/2020	12 LTCFs with 930 residents and 360 staff members	CCS	OCM; TRM	Cohorting of COVID-19 cases; Mask & PPE use	INF/OBS
Rolland 2020 [49]	SARS-CoV-2	France	03/2020 to 05/2020	124 LTCFs	CCS	CRM; TRM	Compartmentalization of staff and residents; Mask & PPE use; Restricted use of shared spaces; Serving meals in room; Cessation of group activities	OBS/CONT
Stemler 2022 [50]	SARS-CoV-2	Germany	10/2020 to 12/2020	65 LTCFs with 685 residents and 596 staff members	CCS	SSM	Routine testing of staff and visitors	OBS/CONT; HOSP/DEATH
Teesing 2020/21 [51–54]	influenza-like illness	The Netherlands	10/2016 to 10/2017	66 LTCFs with 1862 beds	cRCT	TRM	Hand hygiene	INF/OBS
Telford 2020 [55]	SARS-CoV-2	USA	03/2020 to 05/2020	28 LTCFs with 2868 residents and 2803 staff members tested	CCS	SSM	Routine testing of residents and/or staff	INF/OBS; HOSP/DEATH
Tulloch 2021 [56]	SARS-CoV-2	United Kingdom	12/2020 to 01/2021	82 LTCFs	CCS	SSM	Routine testing of staff and visitors	OBS/CONT; INF/OBS
Vijh 2021 [57]	SARS-CoV-2	Canada	02/2020 to 05/2020	7 LTCFs with 1144 residents and 1298 staff members	ITS/RMS	MCM	Combination of multicomponent measures	INF/OBS

*Study Type: cRCT* Cluster randomized controlled trial, *CCS* Controlled cohort study, *ITS/RMS* Interrupted time series analysis / repeated measurement study, *CBA* Controlled before-and-after study | *Intervention domain:* *ERM* Entry regulation measures, *CRM* Contact regulating measures, *TRM* Transmission-reduction measures, *SSM* Screening and surveillance measures, *OCM* Outbreak control measures, *MCM* Multi-component measures | *Outcome category:* *OBS/CONT* Likelihood of outbreaks or contaminations in LTCFs (contaminations referred to at least one case in the facility), *INF/OBS* Number of infections or size of outbreaks (Outbreak referred to at least two cases in the facility from the same assumed source and or at least one case among residents in the facility with the resident likely not having been infected elsewhere), *HOSP/DEATH* Number of severe infections, hospitalizations, or deaths

Most studies (*n* = 13), focused on infection-related outcomes regarding SARS-CoV-2/COVID-19 [35, 38, 39, 41, 42, 44, 45, 48–50, 55–57]. Only three studies—all cRCTs focusing on the effectiveness of hand hygiene interventions—focused on other pathogens or diseases of relevance: respiratory outbreaks [43], influenza-like illnesses [54], and upper-respiratory tract infections [46]. Within the infection-related outcomes, seven studies reported on outbreaks in LTCFs [38, 39, 42, 43, 49, 50, 56], nine studies reported on the number of infections among residents and/or staff [35, 38, 41, 44–46, 48, 54, 56], three studies reported on hospitalizations [38, 45, 55] and five studies reported on the number of deaths due to the pathogens of interest [38, 39, 45, 50, 55]. Notably, no study reported outcomes associated with facility contamination.

We identified 13 NRSI studies [35, 38, 39, 41, 42, 44, 45, 48–50, 55–57] and three cRCTs [43, 46, 54]. One study utilized two LTCFs to either intervention or control group [50]; due to the low number of sites this was classified as NRSI. Among the NRSIs, four employed a cohort-like design, examining the association between intervention presence or absence and outcome over time [39, 42, 48, 49]. Three studies used an ITS-like design [35, 41, 57], using longitudinal data with multiple pre- and post-intervention data points to assess the intervention’s impact on outcome trend or level changes. One study employed longitudinal data for a difference-in-difference analysis [44].

The studies assessed NPIs in LTCFs located in Canada [57], France [38, 39, 48, 49], Germany [50], the UK [42, 56], China—Hong Kong [43], the Netherlands [54, 58], and the USA [35, 41, 44–46, 55].

Six studies exclusively assessed outcomes in residents [39, 41, 44, 46, 48, 54], four investigated both residents and staff members, allowing population group differentiation [38, 45, 55, 57], while five included both residents and staff members without the option for population group stratification [35, 43, 49, 50, 56].

#### Risk of bias and quality of included studies

Among the three RCTs assessed with the RoB 2 tool [43, 46, 54], one study was classified with *some concerns* [43], while two were deemed to have a *high risk of bias* [46, 54].

Upon assessment with ROBINS-I, the comparisons in all but two NRSIs [44, 57] were deemed to exhibit a serious risk of bias, primarily stemming from concerns regarding the domain *bias due to confounding* and the domain *bias due to deviation from intended intervention*. In instances involving the domain *bias due to confounding* it remained uncertain whether observed effects were influenced by underlying LTCF attributes, such as overall LTCF quality, associated with both the presence or absence of the intervention and the outcomes.

Four studies employed a cohort-like design, employing various forms of regression analysis, to estimate the association between intervention and outcome status (e.g., occurrence of an outcome at a later time point) [39, 42, 48, 49]. In these assessments, several were considered to be at *serious or critical risk of bias* within the domain *bias due to deviation from intended intervention*. This was primarily due to inadequate consideration of effects of other implemented infection-control measures that may have varied across intervention and control groups and/or multicollinearity in the assessed measures.

The implementation and adherence to the effective component of the intervention was assessed in the domain *bias due to deviation from intended intervention*. Within this domain, two studies were classified as having a *critical risk of bias* and were consequently excluded from synthesis [50, 56]. Both studies examined the impact of providing opportunities for voluntary self-testing of staff and visitors, with very low adherence to testing observed. Thus, while these studies indicated that within the specific contexts, providing opportunities for voluntary self-testing did not increase testing rates, they did not provide evidence on the effect of routine testing to prevent or mitigate outbreaks in LTCFs.

More details on the risk of bias of included studies is provided in Supplement V.

### Evidence summary on the composite outcome

In the following, the effectiveness of the various measures presented in the included studies to protect residents and staff from infections caused by viral respiratory pathogens with pandemic potential is presented. Table 2 provides a comprehensive representation of all measures, categorized by intervention domain and category. We present the direction of effect plot of the effect measures alongside with the level of certainty of evidence. More comprehensive versions of the composite outcomes and the evidence synthesis can be found in Supplement VI and VII.

**Table 2:**
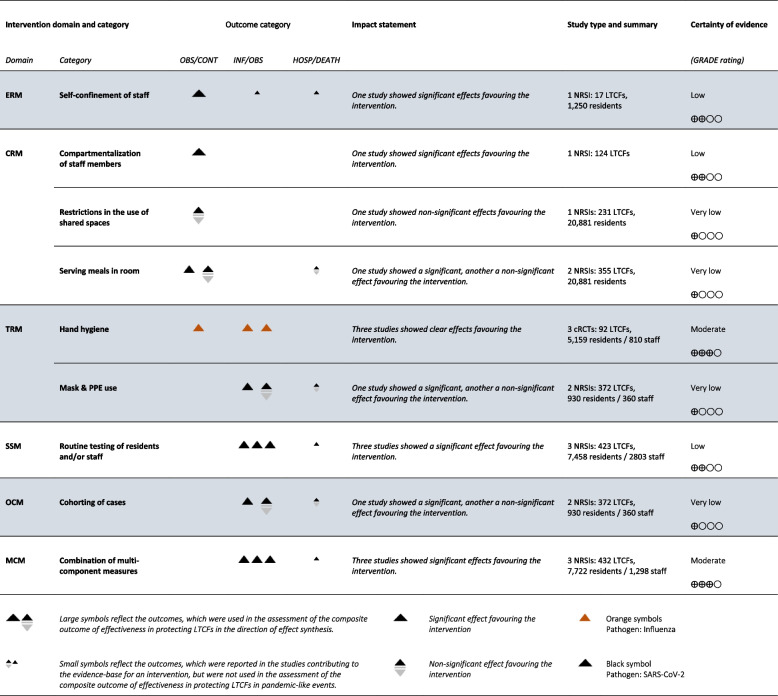
Direction of effect table on the composite outcomes of effectiveness of protecting LTCF residents and/or staff against pathogens with pandemic potential

*Abbreviations:* *(c)RCT* (cluster) Randomized controlled trial, *LTCF* Long-term care facility, *NRSI* non-randomized observational study of intervention effects, *PPE* Personal protective equipment | *Intervention domain*: *ERM* Entry regulation measures, *CRM* Contact regulation measures, *TRM* Transmission-reducing measures, *SSM* Screening and surveillance measures, *OCM* Outbreak control measures, *MCM* Multi-component measures | *Outcome category:* *OBS/CONT* Likelihood of outbreaks or contaminations in LTCFs (contaminations referred to at least one case in the facility), *INF/OBS* Number of infections or size of outbreaks (Outbreak referred to at least two cases in the facility from the same assumed source and or at least one case among residents in the facility with the resident likely not having been infected elsewhere), *HOSP/DEATH* Number of severe infections, hospitalizations, or deaths

### Entry regulation measures (ERM)

We included one observational study [38], that provided evidence on ERMs, namely self-confinement of staff with residents*.* The evidence based on this study conducted in France in the context of the SARS-CoV-2 pandemic suggested that self-confinement of staff may prevent infection-related outcomes due to SARS-CoV-2 (low certainty of evidence).

### Contact-regulating measures (CRM)

We included three observational studies [39, 42, 49] that contributed evidence on multiple different CRMs.

One NRSI conducted in France in the early phase of the SARS-CoV-2 pandemic [49] suggests that compartmentalization of staff members may reduce infection-related outcomes due to SARS-CoV-2 (low certainty of evidence).

The evidence from another NRSI, a case–control study also conducted in France during the SARS-CoV-2 pandemic, suggested that restricting the use of shared spaces [39], may be effective in reducing infection-related outcomes, but the evidence was very uncertain (very low certainty of evidence). The results of two studies also analysing the restriction of shared spaces had been excluded from this synthesis due to critical risk of bias [42, 49].

Furthermore, two NRSIs from France indicated, that serving meals in rooms, may have beneficial effects against infection-related outcomes due to SARS-CoV-2, but the evidence was very uncertain (very low certainty of evidence) [39, 49].

### Transmission-reduction measures (TRM)

We included six studies [43, 45, 46, 48, 49, 54] that contributed evidence on TRMs.

According to three cRCTs [43, 46, 54] conducted in the USA, the Netherlands, and Hong-Kong measures to improve hand hygiene likely reduce infection-related outcomes due to influenza and other upper respiratory tract infections (moderate certainty of evidence). These three studies were the only cRCTs identified in this review and the only studies not focusing on infection-related outcomes in the context of the SARS-CoV-2 pandemic.

We included two NRSIs assessing the effectiveness of mask and the use of personal protective equipment (PPE) to prevent infection-related outcomes due to SARS-CoV-2 [45, 48]. The results of another study, also assessing the effectiveness of mask and PPE use were excluded from this synthesis due to critical risk of bias [49]. One of the included studies conducted in LTCFs in the USA showed clear beneficial effects of mask and PPE use as a measure to reduce the number of infections in LTCFs and unclear beneficial effects regarding the outcome number of deaths [45]. The other study assessed the effects of delayed implementation of generalized mask-wearing in outbreaks of SARS-CoV-2 in French LTCFs. This study found a non-significant increase in the risk of infection per additional day in delay of generalized mask wearing [48]. In summary, the measure may be effective as a strategy to protect LTCFs in the context of a pandemic-like event, however the evidence was very uncertain (very low certainty of evidence).

### Screening and surveillance measures (SSM)

We included three studies that provided evidence on SSMs. These NRSIs conducted in the USA indicated that routine testing of residents and/or staff in LTCFs may be an effective measure to protect against SARS-CoV-2 [35, 41, 55], but there was a high uncertainty in the evidence (very low certainty of evidence). Another two studies focusing on the effectiveness of routine testing of staff and visitors were excluded from the analysis due to critical risk of bias [50, 56].

### Outbreak control measures (OCM)

We included two studies from the USA and from France, that provided evidence on OCMs, namely the separation of infected and non-infected residents (referred to as cohorting) [45, 48]. Both studies were conducted in the early phase of the SARS-CoV-2 pandemic. As both studies showed an effect in favour of the intervention, the measure may be effective as a strategy to protect LTCFs in the context of a pandemic-like event, but the evidence was very uncertain (very low certainty of evidence).

### Multi-component measures (MCM)

We included three studies [44, 45, 57], that provided evidence on the combination of multiple measures across multiple intervention domains (MCM). The three NRSIs showed clear beneficial effects of combining multi-component interventions as a measure to reduce the number of infections [44, 45, 57]. One of these studies also showed clear beneficial effects regarding the reduction of the number of deaths [45]. Two of these studies were the only NRSIs judged to have a moderate, rather than a serious risk of bias among all NRSIs assessed in this review. This evidence on combined multi-component interventions including entry regulation measures, contact regulating measures, transmission-reduction measures, screening and surveillance measures and outbreak control measures suggests that especially a combination of the different components were probably effective in protecting residents and staff (moderate certainty of evidence).

### Evidence-gap map

To identify gaps in the available evidence, we created an evidence-gap map derived from the logic model displayed in Fig. 1. As presented in Table 3, the evidence-gap map reveals a strong concentration on SARS-CoV-2 studies and a dearth of research on other viral, respiratory transmitted pathogens and other than hand hygiene interventions. The map furthermore shows a gap in the available evidence base for several relevant interventions (e.g., the lack of empirical studies on visiting restrictions, air filtration systems, or routine screening/testing using point of care (POC) tests).

## Discussion

### Summary of main findings

We identified 16 unique studies (13 NRSIs and three cRCTs) that assessed the effectiveness of various NPIs on infection-related outcomes intended to safeguard LTCF residents and staff against viral respiratory pathogens with pandemic potential. All but three studies focused on SARS-CoV-2. The remaining three studies examined the effect of hand hygiene interventions against influenza or upper respiratory tract infections. The evidence in this review indicates that especially a combination of measures across multiple intervention domains as well as hand hygiene interventions are probably effective in protecting residents and staff from infection-related outcomes due to viral respiratory pathogens (moderate certainty of evidence). Furthermore, the evidence suggests that the entry-regulation measure of self-confinement of residents with staff, the contact regulation measure of compartmentalization of staff members and the screening and surveillance measure of routine testing of residents and/or staff in LTCFs may be effective in protecting residents and staff (low certainty of evidence). Other measures, such as restricting shared spaces, serving meals in room, separating infected, or non-infected residents may be effective, but the evidence is very uncertain (very low certainty of evidence).

### Risk of bias and certainty of the evidence

The certainty of evidence, as defined within GRADE, was rated as moderate for only two interventions, namely the effectiveness of hand hygiene measures and the combination of multiple different measures. All other NPI assessments were rated as either low or very low. This suggests that the true effects may be substantially different from the estimates reported in this review [60, 61]. The common reasons for downgrading the evidence included concerns about potential risk of bias regarding confounding and imprecision.

Most assessments in the NRSIs were judged to be at high risk of *bias due to confounding*. Although many of these studies were well conducted under the circumstances and with the limited data available at the time, we cannot rule out the possibility that other factors, such as confounding by underlying characteristics of the facilities and/or the population within them or concordantly implemented NPIs, may have influenced the observed associations between intervention and outcome.

We downgraded our certainty of evidence for *imprecision* primarily when the confidence intervals for the effect estimates within a given body of evidence crossed the null effect, our predefined threshold of interest in this review. This would allow for the possibility of the true effect being either in the opposite direction or non-existent.

As specified in our protocol, we intended to assess publication bias through visual examination of funnel plots and the application of tests for funnel plot asymmetry, such as Egger’s tests [62]. However, this approach proved unfeasible in our review due to the scarcity of studies within the same intervention domain and category, falling short of the required minimum of ten comparable studies. Nonetheless, our comprehensive search strategy covered pre-print servers and study-registries, revealing no indication of publication bias for any intervention.

In addressing potential biases, it's key to recognize that intervention effectiveness can be reduced by inconsistent adherence. For instance, despite providing training and resources to enhance hand hygiene, effectiveness might be compromised by poor adherence, misuse of measures like incorrect mask-wearing or handwashing techniques, or the use of inadequate tools such as low-sensitivity point-of-care tests or defective masks. These critical factors are often unreported in studies, potentially compromising the real effectiveness of interventions and leading to underestimation or misinterpretation of their assumed benefits. These issues may conceal an intervention's true efficacy.

### Generalizability of findings

Many studies lacked comprehensive descriptions of the specific LTCFs and their residents, limiting the transferability of findings to other types of facilities like assisted living facilities or populations with varying care needs. The lack of detailed facility and population data as well as the limited availability of evidence from a diverse range of settings (e.g., countries in which a study was conducted) limits the applicability and generalizability of the results beyond the studied context.

All studies were conducted in high-income settings, with the majority being conducted in Western high-income countries (only exception a study from Hong Kong [43]), thereby constraining the generalizability of the findings to low- and middle-income country settings. However, even within western high-income countries, transferability and generalizability of the findings can be limited: The divergent structures of long-term care systems, healthcare funding models, access to care, and policies regarding e.g., infection control practices or staffing levels between these contexts necessitate caution in the adaption and application of the results. While the research gap indicates a need for more research beyond high-income Western countries, a better understanding of contextual factors mediating the effect of the intervention and the feasibility of implementation within different political and long-term care systems is warranted.

Furthermore, most studies predominantly focused on SARS-CoV-2, with limited exploration of other pathogens. Remarkably, no study on hand hygiene interventions focusing on SARS-CoV-2 was identified. Variations in the transmission mode, infectiousness levels, and the relative importance of fomite, droplet, or airborne aerosol transmission plays a significant role in determining the effectiveness of measures. Consequently, the relevance of findings on hand hygiene or screening (to name only two examples) may not directly translate to different pathogens, leaving the transferability of these findings to other infectious diseases or future pathogens uncertain.

In many instances, both the setting and the interventions were inadequately described in the studies. This lack of detail hampers the assessment of the generalizability of their findings to other contexts or intervention strategies.

### Adverse and other unintended consequences of NPIs implemented in LTCFs

While our review primarily focused on the effectiveness of NPIs in protecting LTCF residents and staff during pandemics and severe influenza seasons, we are aware that these measures can impose substantial adverse effects on the mental and physical health of residents (e.g., reduced physical activity, social isolation, depression, and anxiety) [63–69] and staff (e.g., psychological distress or burnout) [70–74]. In our searches, we also included NRSIs investigating the adverse and unintended consequences of NPIs on resident’s and staff’s mental and physical health. See Supplementary material for more details. These findings will be addressed in more detail in a separate publication, including other forms of evidence, such as qualitative study results.

In summary, concerning NRSIs investigating adverse and other unintended outcomes, we identified two NRSIs of high quality conducted in Canada and the Netherlands that assessed adverse effects of lockdown measures, including visiting restrictions and contact reduction measures among residents, during the SARS-CoV-2 pandemic [58, 59]. In these studies, these lockdown measures were accompanied by various measures to reduce potential adverse consequences. After adjusting for multiple testing, neither study found statistically significant adverse effects for several mental health-related outcomes. While NPIs implemented in the setting of LTCFs can lead to severe harmful consequences [75], these studies indicate this may not universally apply for all residents and under all conditions. Moreover, the findings suggest that adverse effects of NPIs may be mitigated through additional countermeasures. Nevertheless, further research with robust study designs is needed to comprehensively assess not only assess the effects of the NPIs against infection related outcomes, but also what additional measures and contextual factors are effective in mitigating harm.

### Strengths and potential biases in the review process

Our review adhered to the systematic, transparent, and reproducible methodology outlined in the *Cochrane Handbook for Systematic Reviews of Interventions* [61]. Furthermore, we employed methods similar to those used in the *Cochrane SARS-CoV-2 LTCF review*, which underwent critical assessment by several content and method experts at both the protocol level and final manuscript level [17, 76]. In order to comprehensively describe the growing bodies of evidence, particularly in the context of the SARS-CoV-2 pandemic, we encompassed not only RCTs but also NRSIs. While these studies inherently possess higher internal validity risks, we consider this a strength of our systematic review, given the impracticality and ethical constraints associated with conducting RCTs during ongoing pandemic-like events. Expanding the scope to NRSIs allowed us to contribute evidence in the absence of RCTs.

We did not include mathematical modelling studies. Although these studies can provide valuable insights, especially where other designs are impractical or unethical (e.g., on the rate of routine surveillance testing), their reliance on a number of assumptions may raise concern about reliability and generalizability. Furthermore, approaches to include them in evidence syntheses are not well established yet [17, 77, 78]. Nevertheless, conducting a systematic review of modelling studies assessing NPIs in LTCFs could yield important insights.

Due to the limited number of studies in each intervention domain and the heterogeneity of reported outcome measures, conducting a meta-analysis was unfeasible. Instead, we adopted a structured narrative synthesis approach, focusing on the direction of effect [28, 31]. Vote-counting, reliant solely on direction, may introduce bias away from the null when small and non-significant findings are present. In order to enhance precision, we consistently applied the terms *unclear* and *clear* effects to distinguish effect estimates with confidence intervals overlapping the null (thus allowing the possibility of a different direction of effect) from those with intervals falling exclusively on one side of the null.

The quality of systematic review findings depends on the quality of the studies conducted on the topic. Many of the studies we identified, including both RCTs and NRSIs, displayed methodological limitations and other deficiencies. It is important to note that these studies, frequently assessing complex interventions in the context of an ongoing pandemic, were not necessarily poorly conducted. To advance the evidence base more robust study designs are needed, with a particular emphasis on quasi-experimental NRSIs.

Furthermore, our searches only include studies published up to September 2022. We therefore do not cover publications which may have been eligible for inclusion but were published after this point. For example, we are aware of such a study conducted on air purification systems in the USA [79].

Infection control within healthcare and long-term care environments has undergone significant evolution and expansion since its inception in the mid-twentieth century, particularly when juxtaposed with practices from earlier periods. This evolution encompasses a heightened safety culture, achieved through organizational modifications in care delivery. These modifications include evaluating effectiveness, along with revising, standardizing, and monitoring procedures, all of which are underpinned by the adoption of evidence-based strategies [80–83]. In particular, the development of guidelines and recommendations in the 1990s, in the wake of the AIDS epidemic, shaped the way modern health care and long-term care infection control practices are conducted [80]. There is a considerable contrast between the IPC practices implemented during the SARS-CoV-2 pandemic and the practices in e.g., the 1950 or 19060 s. To account for these changes and produce evidence of relevance for the current day, we decided to exclude literature published prior to 1992, thus focusing on the most recent three decades of research. However, an exact date at which these gradual changes have occurred cannot be established. Therefore, we consider the line we drew as part of our eligibility criteria as a limitation of our review.

## Conclusions

### Conclusion for research

This review has highlighted several relevant evidence gaps regarding the effectiveness of measures to protect residents and staff from viral respiratory pathogens with pandemic potential, as displayed in the evidence gap map (Table 3).

**Table 3:**
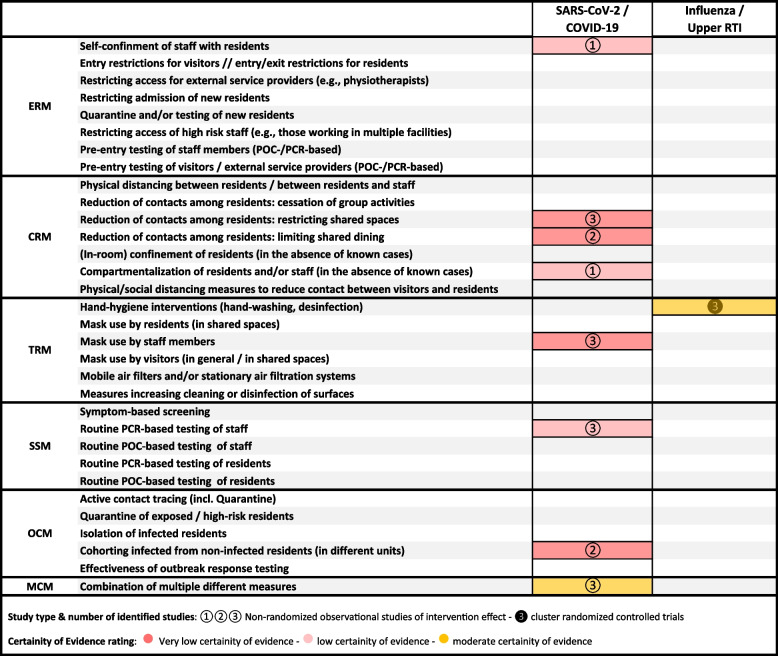
Evidence-gap map

*Abbreviations*: *ERM* Entry regulation measures, *CRM* Contact regulation measures, *TRM* Transmission reducing measures, *SSM* Screening and surveillance measures, *OCM* Outbreak control measures, *MCM* Multicomponent measures, *POC-/PCR-based testing* Point of Care-testing / testing based on polymerase chain reaction, *SARS-CoV2* severe acute respiratory syndrome coronavirus type 2, *COVID-19* Coronavirus disease 2019, *Upper RTI* Upper respiratory tract infections

Addressing these gaps requires the generation of more reliable and robust empirical evidence, to assess the effectiveness and unintended consequences of non NPIs aimed at safeguarding LTCFs against viral respiratory infections. While experimental trials like RCTs are valuable evidence sources, their logistical feasibility during rapidly evolving epidemics and pandemics is challenging. Quasi-experimental designs, such as interrupted time-series analysis, difference-in-difference studies, or propensity score matching can offer practical insights in a timely manner with sufficient reliability of their findings. Public health agencies should consider whether integrating robust evaluation practices can be integrated in their outbreak response and general infection control practices to expand on the body of evidence and provide mode real world data.

In addition, the current body of evidence has a strong focus on SARS-CoV-2, and additional studies addressing infection-related outcomes of other pathogens are needed for almost all interventions except hand hygiene, for which we identified several studies of good to moderate quality.

Moreover, improved reporting is essential. Current studies often inadequately report on interventions, infection control measures, facility information, and contextual details. Enhancing reporting can facilitate the transferability of findings and improve the quality of research within LTCFs.

Furthermore, there is a pressing need for additional research in settings outside Western high-income countries. We did not identify any studies implemented in a low- or middle-income country setting, thereby limiting the generalizability of findings. Research conducted in diverse settings is essential, given the structural and institutional disparities prevalent in long-term care.

Understanding the influence of political priorities, ethical considerations in research, and (existing) limitations in resources like staff, equipment, and funding on implementing NPIs in LTCFs as well as their impact on effectiveness and harmful consequences is crucial. Closing these knowledge gaps can help to facilitate the development context-specific guidelines and recommendations which maximize benefit while minimizing harm within the specific context the LTCFs are placed in.

More research is needed on the effectiveness of various interventions. As displayed in the evidence gap map in Table 3. On many NPIs no reliable evidence was available, while for others only low and very low certainty evidence was available.

### Conclusion for practice

We have identified several measures that may serve as effective strategies to protect residents and staff in LTCFs against outbreaks, infections, and their associated consequences in future pandemic-like events. Our evidence suggests that the implementation of NPIs in combination with each other is crucial to fully realize their potential [44, 45, 57]. However, it is important to note that, with some exceptions, the certainty of the evidence was *low* to *very low*. This reflects the inherent challenges of generating robust evidence in the context of pandemics, where conducting rigorous RCTs with a high internal validity is often technically infeasible or ethically unjustifiable. Given the high burden of morbidity and mortality in LTCFs without adequate vaccination coverage, implementing NPI measures identified as potentially effective is often the only reasonable option available until satisfactory vaccination rates can be achieved, despite concerns about the certainty of evidence.

Although we did not find evidence to numerous important interventions, including visiting restrictions, pre-entry testing with point-of-care tests, quarantine and isolation, air filters, or improved environmental hygiene, this absence of evidence should not be equated with evidence of absence [84]. The lack of direct evidence from LTCFs setting should not hinder the implementation of these measures if expert judgement from practitioners working in the field, modelling studies, evidence from other settings, or other sources of evidence indicate their benefits and necessity.

Given the gaps in the evidence base regarding the effectiveness of these NPIs, their implementation should always be accompanied with appropriate evaluations mechanisms to avoid allocating resources to ineffective measures and to close these evidence gaps in the future.

Many NPIs assessed in this review pose a substantial infringement of the individual rights and liberties of one of the most vulnerable populations in our society. During the SARS-CoV-2 pandemic, many LTCF residents spent their last weeks or months confined to their room and in isolation from family and friends. This raises questions about the potential benefit in terms of reduction of infection-related outcomes and the need to critically evaluate and balance them against potential harms. NPIs should always be accompanied by adequate countermeasures to assess and mitigate adverse consequences.

Considering the low certainty of evidence regarding the effectiveness of these NPIs and the potential yet uncertain harm they may cause, careful deliberation processes are imperative. We have proposed the WHO-INTEGRATE COVID-19 (WICID) framework as a tool to aid public health practitioners, public health experts, and public health policy decision makers in navigating this complex task [85, 86]. WICID aims to facilitate the development of effective strategies to safeguard LTCF residents and staff during future epidemics and pandemics [44, 45, 57].

In summary, our review highlights the gaps in the evidence and the need for additional research, but also provides a comprehensive and systematic review of the available measures potentially effective in protecting residents and staff of long-term care facilities in the pandemics to come.

### Supplementary Information

**Supplementary Material 1.**

## Data Availability

All data generated or analysed during this study are included in this published article. They are based on research articles available in the public domain.

## Abbreviations

AUC: Adverse and other unintended outcomes
CBA: Controlled before-and-after study
CINAHL: Cumulative Index to Nursing and Allied health Literature
(c)ITS: (Controlled) interrupted-time-series analysis
COVID-19: Coronavirus disease 2019
CRM: Contact regulation measure
(c)RCT: (Cluster) Randomized Controlled Trial
ECDC: Europen Centre for Disease Prevention and Contol
EPIET: ECDC Fellowship Programme, Field Epidemiology Path
ERM: Entry regulation measure
GRADE: Grading of Recommendations, Assessment, Development and Evaluation
LTCF: Long-term care facility
MCM: Multi-component measure
MERS: Middle East Respiratory Syndrome
NPI(s): Non-pharmacological intervention(s)
NRSI: Non-randomized observational study of intervention effects
OCM: Outbreak control measure
PI(s): Pharmacological intervention(s)
PPE: Personal protective equipment
PCR: Polymerase chain reaction
PCR/RCS: Controlled prospective / retrospective cohort study
POC: Point of care
RD: Regression discontinuity
RMS: Repeated measurement study
RNA: Ribonucliec acid
RoB: Risk of Bias
ROBINS-I: Risk Of Bias In Non-randomised Studies-of Interventions
RTI: Respiratory tract infection
SARS: Severe acute respiratory syndrome
SARS-CoV-2: Severe acute respiratory syndrome coronavirus type 2
SSM: Screening and surveillance measure
SWIM: Synthesis without meta-analysis
TRM: Transmission reduction measure
WHO: World Health Organization
WICID: WHO-INTEGRATE COVID-19
