# Supplementary material for: How to protect long-term care facilities from pandemic-like events? - A systematic review on the effectiveness of non-pharmacological measures to prevent viral respiratory infections
Source: BMC Infect Dis. 2024 Jun 17;24:589. doi: 10.1186/s12879-024-09271-7 (PMC11181531; doi:10.1186/s12879-024-09271-7)
Supplement: Supplementary file 1 — Supplementary Material 1. [file 12879_2024_9271_MOESM1_ESM.docx]

# Supplementary material

[Supplementary material 1](#_Toc149737172)

[1. Appendix Ia: Extended description of eligibility criteria 3](#_Toc149737173)

[1.1. Types of studies 3](#_Toc149737174)

[1.2. Types of interventions 4](#_Toc149737175)

[1.3. Types of settings 7](#_Toc149737176)

[1.4. Types of populations 7](#_Toc149737177)

[1.5. Types of comparators 8](#_Toc149737178)

[1.6. Types of outcomes 8](#_Toc149737179)

[1.7. Publication date 9](#_Toc149737180)

[1.8. Language restrictions 9](#_Toc149737181)

[2. Appendix Ib: Eligibility criteria 10](#_Toc149737182)

[3. Appendix II: Search strategy 12](#_Toc149737183)

[3.1. COVID-19 specific electronic searches 12](#_Toc149737184)

[3.2. Not COVID-19 specific electronic searches 15](#_Toc149737185)

[4. Appendix III: List of reviews used in the reference searches 23](#_Toc149737186)

[5. Appendix IV: Extended Methods section 25](#_Toc149737187)

[5.1. Risk of bias assessment 25](#_Toc149737188)

[5.2. Minimal threshold for public health relevance 26](#_Toc149737189)

[5.3. Intervention effect of interest 26](#_Toc149737190)

[5.4. Measures of intervention effect 26](#_Toc149737191)

[5.5. Data synthesis and analysis 27](#_Toc149737192)

[5.6. Assessment of reporting/publication biases 28](#_Toc149737193)

[5.7. Assessing heterogeneity 28](#_Toc149737194)

[5.8. Assessment of certainty of evidence and creating summary of findings (SoF) tables 29](#_Toc149737195)

[6. Appendix V: Extended description of risk of bias of included studies 29](#_Toc149737196)

[6.1. Risk of bias of included randomized controlled trials 29](#_Toc149737197)

[6.2. Risk of bias of included observational studies 30](#_Toc149737198)

[7. Appendix VI: Effectiveness of NPIs as strategies to protect LTCF residents and staff from infection-related outcomes of viral respiratory pathogens with pandemic potential 34](#_Toc149737199)

[7.1. Entry regulation measures 34](#_Toc149737200)

[7.2. Contact-regulating measures 34](#_Toc149737201)

[7.3. Transmission-reduction measures 37](#_Toc149737202)

[7.4. Screening and surveillance measures 39](#_Toc149737203)

[7.5. Outbreak control measures 40](#_Toc149737204)

[7.6. Combination of multiple measures across multiple intervention domains 41](#_Toc149737205)

[8. Appendix VII: Summary of Findings tables 43](#_Toc149737206)

[9. Appendix References 47](#_Toc149737207)

# Appendix Ia: Extended description of eligibility criteria

## Types of studies

We included both (cluster) randomized controlled trials (RCTs / cRCTs) as well as non-randomized observational studies of intervention effects (NRSI). While we recognized that NRSIs are at higher risk of bias compared with RCTs, a previous scoping of the literature had indicated, that very few RCTs on the topic were available, in particular regarding measures implemented in the context of the SARS-CoV-2 pandemic. Primarily, as due to the rapid progression and severe consequences of no or delayed actions during health emergencies, it often was considered not appropriate, feasible, or ethical to conduct rigorous RCTs (1, 2). Thus, a sole reliance on RCTs would have prevented us from providing any evidence for most important groups of interventions.

We included the following types of studies:

1. (cluster) randomized controlled trials (RCTs / cRCTs)
2. non-randomized observational studies of intervention effects (NRSIs) (3, 4) which allow control of both observed and unobserved confounding. This includes: quasi-RCTs (Q-RCTs), controlled before-and-after (CBA) studies, (controlled) interrupted-time-series (ITS / cITS) studies, instrumental variable (IV) studies, propensity score matching (PSM) studies, and regression discontinuity (RD) studies; as defined by Reeves et al. (4).
3. controlled prospective and retrospective cohort studies (PCS / RCS), that have the following characteristics:

- the study is a non-randomized observational study which intends to evaluate the effect of an NPI implemented in LTCFs
- the assessment is based on systematically collected, quantitative data on at least one of the outcomes of interest, with at least one measurement of the data in both the intervention and control group, collected after the implementation of the intervention or the respective control
- the study allows the effect of the intervention to be estimated, based on differences between groups of individuals or clusters receiving either the intervention of interest or a comparator (this includes comparing the extent of change over time between groups)
- the study allows for establishing the chronological order of introducing the intervention and its effects on outcomes (i.e., the intervention having caused the outcome, rather than the outcome leading to the introduction of the measure)

In line with the Cochrane EPOC guidance (2), we limited cluster-RCTs, NRCTs, CBA studies, as well as PCSs and PRCs to those with at least two intervention and control sites (in our case LTCFs, wards, or similar units of analysis). For ITS/cITS-like, studies, limited the inclusion to those studies with a clearly defined intervention time point and at least three data points before and after the interruption (i.e., the intervention). We considered studies published in scientific journals, as well as those published on preprint servers (e.g., medRxiv) and in the grey literature.

We did not to include:

- NRSIs without a control group or other means of approximation of a counterfactual (e.g., uncontrolled before-and-after studies).
- quantitative empirical studies which do not allow for a chronological distinction between implementation of intervention and outcome measure (e.g., cross-sectional studies)
- studies that did not provide a quantitative measure of impact (e.g., qualitative studies)
- mathematical modelling studies (although we systematically collected the references of mathematical modelling studies which fulfilled the other eligibility criteria)
- diagnostic test accuracy studies (i.e., studies assessing the sensitivity and specificity of different screening tests, which did not intend to assess the effects of (real-world) application of routine use of testing on the outcomes of interest)
- studies that did not provide primary empirical data (e.g., commentaries, editorials, literature reviews not focusing on primary empirical data)
- systematic and literature reviews (although those we systematically collected and used for reference searches of relevant literature)
- conference abstracts and summary reports, since these do not report sufficient data on population, intervention, comparison, outcomes, and settings to allow an assessment of their eligibility

## Types of interventions

We included studies that assessed the impact of NPIs implemented in LTCFs (as specified in the next section) in preventing infections with pathogens with pandemic potential as well as similar respiratory viral infections. These included multiple different sets of measures in the intervention domains:

- **Entry regulation measures (ERM):**
  Measures intended to limit the introduction of the pathogen into the facility, primarily by preventing contagious individuals or individuals with an increased risk of being contagious from entering LTCFs. For example, but not limited to:
  - **Access restrictions:** Defined as temporarily restricting access to LTCFs to all or some population groups. This can include limiting or prohibiting visitors from entering the facility, limiting the (re-)admission of residents to the facility, or limiting the access to certain groups of staff (e.g., agency staff).
  - **Pre-entry screening:** Defined as using rapid point-of-care testing or symptom-based screening to identify individuals with an increased likelihood of being infectious. This can include pre-entry testing or screening of visitors or of staff members prior to entry in the facility as well as symptom-, point of care (POC)-based testing or screening of residents being (re-)admitted to the facility.
  - **Quarantine of new admissions:** Defined as quarantining newly (re-)admitted residents e.g., until negative PCR test results are available or for a specified period of time.
  - **Self-confinement of staff members with residents:** By this we mean (voluntary) self-confinements made by staff. This includes interventions where staff temporarily reside in the LTCF in order to reduce the risk of becoming infected in the community and carrying the pathogen into the facility.
- **Contact regulating measures (CRMs)**
  Measures intended to reduce the number of contacts at risk of transmission within the facility. This included contacts among and between relevant groups in the LTCF, including residents, nursing staff, visitors, and other non-nursing staff, such as:
  - **Measures to reduce contacts between residents:** Defined as measures where residents are confined to their rooms, reducing the sizes of groups of residents during social or communal activities (e.g., staggered dining times to reduce the number of residents in a common room), closing of communal spaces, or halting social events (e.g., religious services).
  - **Measures to reduce contacts between staff members:** This included organizational and structural measures intended to reduce the contact between and among nursing staff and non-nursing staff members. This may include measures to reduce the contact between shifts (e.g., digital or written handover between shifts), measures to reduce the number of contacts between different staff members (e.g., through organizing the shift planning), as well as measures to reduce contact between staff members during shifts (e.g., staggered break times).
  - **Compartmentalization** **within the LTCF:** Defined as organizational or structural measures to reduce the contact between units within the LTCF (e.g., wards or floors). For example, limiting the movement of residents or staff members between the units or floors or measures to reduce contacts between staff members and residents to a minimum (e.g., by fixed care arrangements).
- **Transmission-reduction measures (TRMs)**
  Measures to reduce the risk of transmission from droplets or airborne particles upon contact within the facility as well as from transmission through fomites.
  - **Respiratory protective equipment:** Defined as measures focused on staff, visitors, and/or residents wearing masks (e.g., surgical masks, N95 respirators) in specific situations or for specific tasks to reduce the risk of respiratory transmissions.
  - **Other personal-protective equipment (PPE):** This includes the use of PPE other than respiratory protective equipment (e.g., protective clothing, gloves, visors, and face shields) by staff, visitors and/or residents in specific situations or for specific tasks.
  - **Hand hygiene and other personal hygiene practices:** Including measures focused on improving hand hygiene (e.g., hand washing, using hand sanitizer) by staff, visitors and/or residents in general or focused on specific situations or for specific tasks.
  - **Environmental hygiene:** Measures aiming to reduce the risk of transmission through fomites by improving the cleaning or disinfecting of environmental surfaces. For example, increasing the frequency of cleaning or disinfection of high-touch surfaces.
  - **Physical or structural environmental barriers:** Installing physical barriers in the facility; for example glass barriers to limit direct contact between staff, residents and/or visitors.
  - **Improving air quality:** Measures to reduce respiratory transmission through improving air quality, for example by installing air filters or through improving ventilation.
- **Surveillance and testing-based measures (STM)**
  Measures to detect cases early or to identify asymptomatic but contagious individuals, in combination with appropriate countermeasures (e.g., quarantine and isolation).
  - **Symptom-based testing or screening:** Measures such as checking residents for disease-specific symptoms (e.g., fever) on a regular basis, likely followed by more reliable approaches of testing for the disease and/or quarantine/isolation measures.
  - **Regular POC or PCR-based testing:** Residents or staff members being tested on a regular basis using POC or PCR-based tests (e.g., weekly PCR-based tests). This measure is likely often combined with quarantine or isolation measures.
- **Outbreak control measures (OCM)**Measures implemented in the facility with the intention to interrupt or prevent further spread of an outbreak after a case of the respective disease detected within the LTCF.
  - **Contact-tracing and targeted quarantine:** Measures aimed at identifying contact persons of cases, followed by a targeted quarantine of contact persons.
  - **Generalized testing approaches:** Generalized testing of all residents and staff members in a specific unit (e.g., ward, floor, or the facility as a whole) after a case of the respective disease was detected within this unit.
  - **Quarantine or isolation:** Quarantine of individuals with a high risk of infection (e.g., individuals showing symptoms or being a contact person of cases) and/or isolation of individuals tested positive for the disease of interest. This includes staff abstaining from work in the case of being found to be at elevated risk of being infectious (e.g., for having had a risk contact) or in the case of being tested positive for the disease of interest.

We did not include:

- Studies on pharmacological interventions (e.g., medication, vaccines).
- Studies which do not assess or allow to determine the effect of an NPI of interest.
- Studies including interventions which aimed to assess the effect of NPIs primarily intended to mitigate the adverse effect of interventions implemented to protect residents from viral respiratory infections (e.g., video-calls to reduce loneliness of residents during times of visiting restrictions).
- Studies investigating institutional-level risk factors for transmission-related outcomes in LTCFs. In this context, we defined risk factors as characteristics of, or practices in, LTCFs that (1) have not been specifically implemented or modified to protect residents from (respiratory) infections and (2) can be hypothesized to explain differences in disease burden across and between several LTCFs (relevant risk factors include: e.g., number of beds in a facility, staff-to-bed ratio, share of single rooms, reliance on agency staff, availability of outdoor spaces). However, we would have include studies, which assessed measures actively addressing these risk factors, such as moving all residents into single rooms or limiting the access of agency staff to the facility.

In situations, were the distinction between NPI and risk-factor was not clear, this was discussed within the research team until a decision was reached.

## Types of settings

In line with the Cochrane SARS-CoV-2 LTCF review (1), we focused on NPIs implemented in the setting of LTCFs, employing a broad understanding of this setting. LTCFs were thereby defined as residential institutions which provide care for people who require support due to experiencing difficulties in living independently in the community. These difficulties may result from physical, mental, intellectual, or sensory impairments, possibly related to old age or chronic medical conditions. While different terms are used for the various institutions falling under this umbrella, in this review we used the term long-term care facility and the abbreviation LTCF to also encompass skilled nursing facilities, nursing homes, retirement homes, assisted-living facilities, residential care homes, or other similar facilities or institutions (5).

We excluded studies reporting on measures implemented in facilities or institutions which primarily or exclusively provide medical care or medical treatment (e.g., hospitals), specialized forms of medical (short-term) care (e.g., rehabilitation centres), or (specialized) palliative care facilities (e.g., hospices).

Furthermore, we did not include home care and care provided in home care services, where a person receives (ambulatory) nursing care or other medical and social support through family members, (specialized) home care nursing or social services, but does not reside within a LTCF.

In addition, facilities which were primarily or exclusively focused on paediatric populations were not included. We defined this as more than 75% of the population receiving care in the institution being under 18 years old.

Within this review, a measure was considered implemented in a LTCF if it was:

1. implemented within the building and/or the LTCF premises (e.g., air filters installed in the building),
2. directly affecting the practices of daily living of residents residing in the facilities (e.g., limiting social activities) or practices of staff when working within the facilities (e.g., mask-mandates for staff when providing care to patients),
3. implemented or practiced outside the LTCF premises but when the measure was intended to directly affect the risk of individuals introducing the pathogen into the facility (e.g., requiring visitors to conduct PCR-tests before coming to the LTCF), or
4. when the measure is implemented on a systemic or structural measure but aims to support or facilitate the conduct of one of the measures above (e.g., a national level policy providing sick-pay for nursing staff).

Measures which are not specifically implemented in or targeting the setting of LTCF were not included (for example, general lockdown measures, travel restrictions, or school closures), even if the study aims to assess the outcome of these measures on populations working or residing in LTCFs.

## Types of populations

We included studies assessing the outcomes of interest for at least one of following population groups:

- adult residents (≥18 years) primarily residing in LTCFs
- individuals visiting residents if the LTCFs
- staff working in the setting of LTCFs

## Types of comparators

We included studies that assessed the impact of NPIs by relying on the following comparisons:

- Measure versus no measure (e.g., LTCFs with vs. without a mask-mandate). We were aware however, that intervention as well as control group would have some form of baseline infection prevention and control measures in addition to the measure of interest in place).
- More stringent versus less stringent implementation of a measure (e.g., a daily vs. weekly PCR-based testing of staff).
- Measure versus an alternative measure (e.g., LTCFs with mandatory POC-based testing vs. LTCFs with visitor restriction).
- Earlier versus later implementation of a measure (e.g., implementing generalized testing sooner vs. later after the detection of a case).

## Types of outcomes

In this review, we assessed both infection-related outcomes (including: number of infections, outbreaks, and deaths) as well as health-related adverse and other unintended consequences of the intervention.

Regarding the infection-related outcomes, we include studies on viral respiratory pathogens that had already occurred in the context of a pandemic or epidemic (e.g., SARS-CoV-2, SARS-CoV, MERS-CoV, 2009-H1N1 Influenza). Furthermore, included studies on illnesses caused by viral pathogens which are transmitted via the respiratory route, which are similar to those illnesses caused by the viral, respiratory pathogens which have caused epidemics or pandemics in the past (i.e., influenza-like illnesses, acute respiratory tract infections).

We were aware, that a substantial burden of respiratory tract infections, in particular lower respiratory tract infections, occurs in the context of aspiration pneumonia. While measures to reduce aspiration pneumonia are important, we considered this beyond the scope of our study. We therefore include studies reporting on this outcome, if it could be assumed that a substantial proportion of those infections (≥ 50 %) were caused by viral, respiratory pathogens (e.g., upper respiratory tract infections) and when there is no indication, that the intervention effect is primarily due to a reduction in the proportion of non-viral infections (e.g., as would be the case for aspiration pneumonia).

We excluded studies which focused on infections

- due to non-viral pathogens
- infection-related outcomes caused exclusively or primarily by non-viral pathogens (e.g., lobar pneumonia or aspiration pneumonia), as well as
- infections caused by viral pathogens that are members of the family of pathogens with an increased pandemic potential (6) (i.e., orthomyxoviruses, paramyxoviruses, pneumoviruses, coronaviruses, and some picornaviruses), but that are not (primarily) transmissible via the respiratory route and/or do not (primarily) cause respiratory tract infections (e.g., poliovirus or hepatitis A virus).

In line with the WHO-INTEGRATE COVID-19 (WICID) framework (7) and the Cochrane review on NPIs implemented in LTCFs in the context of SARS-CoV-2 (1), we include studies assessing any of the following primary outcomes:

1. **Infection-related outcomes (informing on the effectiveness of the measure to protect LTCFs against pandemic-like events)**
   - 1. Number, rate, or proportion of viral respiratory infections due to the viral respiratory pathogen (e.g., laboratory-confirmed SARS-CoV-2 infections, influenza infections or infections with other viral respiratory pathogens, such as acute respiratory tract infections or influenza-like illnesses)
     2. Number, rate, or proportion of contaminations of LTCFs due to the viral respiratory pathogens of interest (This refers to LTCFs contaminated by viral respiratory pathogens. In this context, contamination of LTCFs refers to LTFCs with at least one case infection in the observation period which was introduced from outside of the LTCF)
     3. Number, rate, or proportion of outbreaks in LTCFs due to the viral respiratory pathogens of interest (This refers to LTFCs with more than one infection of the pathogen of interest and/or situations, were it can be assumed that at least one infection had occurred within the LTCF)
     4. Number, rate, or proportion of hospital admissions due to the viral respiratory pathogen of interest
     5. Number, rate, or proportion of deaths due to the viral respiratory pathogen of interest
2. **Adverse and other unintended mental or physical health outcomes (e.g., rate of residents experiencing loneliness) of NPIs**

We did not assess any secondary outcomes in this review.

## Publication date

To reflect changes in (medical) care and public health practice but in order to include publications from the 2003 SARS-pandemic, we restricted searches to the past 30 years (i.e., in or before 1992). It should be noted however, that we did not implement this filter within the database searches and did not exclude any study based on this exclusion reason. The search cover the period up to September 2022, as this was the day the searches were conducted.

## Language restrictions

We considered studies published in English, French, German, Italian, and Spanish. We excluded studies in languages other than those listed. This decision was based on the language skills available within the research team. The WHO COVID-19 Global literature on coronavirus disease registry also included abstracts in other languages (e.g., Chinese). We planned to attempt to acquire and translate of publications which were considered to be of relevance, however did not identify any such record.

# Appendix Ib: Eligibility criteria

Table: Inclusion and Exclusion criteria in PICO-S-ST

|  | **Inclusion (Label)** | **Exclusion (Reasons)** |
| --- | --- | --- |
| **Population** | Study focuses on **adult residents** living in LTCFs or nursing staﬀ or non-nursing staﬀ or individuals visiting LTCFs on a regular or irregular basis (for work and non-work-related purposes) or other individuals directly affected by measures implemented in LTCFs.   - Study focuses on several populations, one of them being residents/staff/visitors of LTCFs **[Label: SUBPOPULATION]** - If a study addresses the interventions of interest in nursing staff, where it is unclear whether this may allow for an specific assessment of the outcomes within LTCF nursing staff **[Label: NURSING.STAFF.UNCLEAR]** | - Study focuses on the **general population** - Study focuses primarily on individuals not living and/or working in the LTCF - Study focuses primarily or exclusively on pediatric populations (> 75% of the study population is younger than 18 years old) **[Reason: PAEDIATRIC]** |
| **Intervention** | Study assesses a measure implemented with the intention to protect residents or staff of LTCFs from viral, respiratory-transmissible pathogens with RNA genomes  The intervention aims to prevent **respiratory diseases caused by COVID-19 or other viral pathogens**  **PATHOGENS & DISEASES** to be included  (In particular: orthomyxoviruses, paramyxoviruses, pneumoviruses, coronaviruses, and picornaviruses)   - **[Label: COVID19]**   - **SARS-CoV-2 / COVID-19 / Coronavirus disease** - **[Label: INFLUENZA]**   - **Influenza**   - **Influenza-like-diseases** - **[Label: OTHER.PATHOGEN]**   - **SARS**   - **MERS**   - **mumps**   - **rubella virus**   - **measles**   - **Morbillivirus**   - **Rhinovirus**   - **Respiratory Syncytial Virus Infection**   - **Human Metapneumoviruses** - **[Label: PATHOGEN.UNCLEAR]**   - Intervention aims to prevent **respiratory infections** (incl. rhinitis); but the causative agent is unclear / not defined / not studied - Intervention targets **bacterial AND viral** pathogens that cause respiratory infections | Study does not assesses a measure implemented with the intention to protect residents or staff of LTCFs from viral, respiratory-transmissible pathogens with RNA genomes **[Reason: NO.INTERVENTION]**  **PATHOGENS & DISEASES** to be excluded   - **Diseases caused by bacterial pathogens or by parasites**   - **Pneumococcus**   - **Legionella pneumophila**   - **Legionnaires' disease**   - **Pneumococcal pneumonia**   - **Tuberculosis**   - **Chlamydia**   - **Staphylococcus**   - **Streptococcus**   - **Salmonella**   - **Giardia**   - **Bordetella pertussis**   - **Aspiration pneumonia** - **Diseases** caused by viral pathogens that do **not primarily cause respiratory diseases**   - **Norovirus infections**   - **Poliovirus infections**   - **Coxsackievirus infections**   - **Rotavirus infections** |
|  | NPI   - The measure is a non-pharmacological **intervention** (NPI) | - The measure is a pharmacological intervention (e.g., prophylactic drug treatment of patients in LTCFs). - The labels assess the efficacy, effectiveness, or impact of a vaccine within the context of LTCFs |
|  | CAVE: Measures/interventions focusing on **oral health**   - measures/interventions that focus on oral health as a non-pharmacological intervention (e.g., gargling as NPI) **[label: ORAL.HEALTH]** | - respiratory tract infections as a healthcare-associated infection will be excluded **[Reason: ORAL.HEALTH]** - oral health care measures targeting **aspiration pneumonia** will be excluded (as mostly bacterial) **[Reason: ORAL.HEALTH]** |
| **Comparison** | Intervention (“experimental” character)   - The measure is a **deviation from the regular praxis** conducted outside the context of a pandemic or epidemic (e.g., basic food hygiene). | - **Studies** that did not collect empirical data on possible interventions (but all other PICO criteria in place) will be excluded but labelled accordingly **[Reason: LIKELY.NOT.EMPIRICAL]** |
| **Outcomes** | Study reports on at least one of the following primary outcome categories.   - **infections avoided** due to the measure - **Contaminations of LTCF**s avoided due to the measure - **Outbreaks in LTCFs** avoided due to the measure - related **hospitalizations avoided** due to the measure - related **deaths avoided** due to the measure - **Adverse and other unintended outcomes** see notes **[Label: AUC]** *--> see notes* | Study reports none of the primary outcome categories **[Reason: OTHER]**, but e.g., on one of the following:   - **societal or ecological outcomes** (e.g., changes in waste production or energy consumption) - **economic or financial outcomes** (e.g., studies estimating cost or resource use of an intervention) or(other) - **implementation-related outcomes** (e.g., reported acceptability or adherence to the measure, reported barriers for implementation) |
|  |  | **Test accuracy studies [Reason: TEST.ACCURACY.STUDY]**:   - Studies which exclusively or primarily are interested in **testing a diagnostic device** (e.g., a point of care test) within the context of an LTCF. - If the main interest of the study is to assess s**ensitivity or specificity of the test**, then this should be excluded. If it focuses on other outcomes (e.g., infections avoided, then it should be included) |
| **Setting** | - The measure is implemented in the **setting of long term care facilities (LTCFs)** *--> see notes* | - The measure is i**mplemented outside the setting of LTCF**s, independent of the effect on transmission within LTCFs (e.g., school closures affecting the transmission in LTCFs through the overall pandemic progression) - The measure is implemented in facilities primarily or exclusively providing one of the following:   - acute (medical) care (e.g., **hospitals**)   - rehabilitative care (e.g., **rehabilitation facilities**)   - palliative care. (e.g., **hospices**) |
|  |  | - The measure is implemented **in the setting of home** care and home care services **[Reason: HOMECARE]** |
|  |  | - LTCFs that are primarily or exclusively **focused on paediatric populations** (> 75% of the population is <18 years old) will be excluded **[Reason: PEDIATRIC]** |
| **Study types** | **Empirical quantitative data**   - Study provides **quantitative data** on the outcomes of interest - Study is a RCT, cRCT, NRCT, CBA, or ITS -*-> see notes* **[Label: EXPERIMENTAL]** - Study is another quantitative study design (e.g., case-control study, cross-sectional study, cohort study) **[Label: OBSERVATIONAL]** | - Study provides **only qualitative data** on the outcomes of interest **[Reason: QUALITATIVE]** - Study is a non-comparative study  (e.g., case series) - Study is an opinion paper, editorial, commentary - Study is a mathematical modelling study **[Reason: modelling]** - Publication is a conference abstract **[Reason: CONFERENCE.ABSTRACT]** |
|  | **Outbreak studies:**   - The study reports on a **COVID-19-related outbreak in a LTCF** **[Label: OUTBREAK.STUDY]** *The publication reports on one or more COVID-19 outbreaks in depth. This includes, description e.g., of the course of the outbreak, the number of infected individuals, and which measures were taken. Here, the other PICO criteria do not necessarily have to be fulfilled* | - **Outbreak studies:** The study reports on an outbreak in a LTCF with a pathogen involved, we’re not focusing on  *→ see Population* **[Reason: OUTBREAK.STUDY]** |
|  | **Risk factor studies [Label: RISKFACTOR.STUDY]**   - **Studies on institutional risk factors** will be included: The study assesses which risk or protective factors of or in relation to a LTCF increase or decrease the risk of an outbreak in the facility and/or an adverse course of the participants - Studies that identify **structural/environmental factors in LTCFs as modifiers** (e.g., studies that recognize that LTCF size and amenities have a relevant impact on infection risk --> LTCF contextual factors as "intervention") | - **Individual level risk factors** will be excluded: Studies which assess individual level factors of staff or residents which predict a good or worse course of the disease (e.g., a study assessing, whether or not high blood pressure or older age leads to a higher mortality rate among LTCF residents if they are infected with the disease) - **Risk factor studies, only identifying living in a LTCF as risk factor**: Epidemiological studies that assesses risk factors (e.g., high risk of transmission or mortality) and conclude, that living in an LTCF / higher number of LTCFs in an area is a risk factor - without identifying “modifying” characteristics of the LTCFs (e.g., size, structure, staff-to-bed-ratio) |
|  | **Systematic Reviews [Label: REVIEW]**   - **All (systematic) reviews** focusing on the prevention of respiratory diseases caused by COVID-19 or other viral pathogens within the context of LTCFs will be included for snowballing |  |
| **Publication date and language** | **Publication date**   - studies published before 1992 will be excluded   **Language restrictions**   - Studies published in English, French, German, Italian, and Spanish will be included - We will exclude studies in languages other than those listed. | - studies published before 1992 will be excluded   **[Reason: TOO.OLD]**   - Studies published in another language than English, French, German, Italian, and Spanish will be excluded **[Reason: FOREIGN.LANGUAGE]** |

# Appendix II: Search strategy

Our approach to identifying relevant studies was based on database searches in multiple databases, reference searches, as well as contacting authors. The identification of literature through databases consist of two components: a component addressed SARS-CoV-2-focused literature while a second component focused on other pathogens of interest. The first component focused on literature searches on SARS-CoV-2 using two SARS-CoV-2 specific study registries as well as the database CINAHL EBSCO. The second component focused on literature searches on respiratory tract infections and infection of other relevant pathogens in the databases Embase, MEDLINE, and CINAHL EBSCO.

## COVID-19 specific electronic searches

The SARS-CoV-2-focused component was based on the database searches of the Cochrane SARS-CoV-2 LTCF review (1), which was updated for the period January 2021 to September 2022. The search strategy consisted of terms focused on SARS-CoV-2/COVID-19 and terms related to the setting of LTCFs. Here, we searched the specialized SARS-CoV-2-focused study registers Cochrane COVID-19 Register and WHO COVID-19 Global literature on coronavirus disease, as well as CINAHL EBSCO (Cumulative Index to Nursing and Allied Health Literature). The two specialized registers are comprised of literature focused on SARS-CoV-2/COVID-19 derived from regular searches in, among others, the databases PubMed, Embase, Central, Web of Science, PsychInfo, as well as medRxiv.

### Cochrane COVID-19 Study Register

| 1 | "nursing home" OR "nursing homes" OR "care home" OR "care homes" OR "nursing residence" OR "nursing residences" OR "nursing residency" OR "nursing residencies" OR "nursing care facility" OR "nursing care facilities" OR "nursing care home" OR "nursing care homes" OR "nursing care residence" OR "nursing care residences" OR "nursing residency" OR "nursing residencies" OR "senior citizen home" OR "senior citizens home" OR "senior citizen homes" OR "senior citizens homes" OR "senior citizen facility" OR "senior citizen facilities" OR "senior citizens facility" OR "senior citizens facilities" OR "senior citizen residence" OR "senior citizen residences" OR  "senior citizen residency" OR "senior citizen residencies" OR "senior citizens residence" OR "senior citizens residences" OR "senior citizens residency" OR "senior citizens residencies" OR "assisted living facility" OR "assisted living facilities" OR "assisted living home" OR "assisted living homes" OR "assisted living residence" OR "assisted living residences" OR "assisted living residency" OR "assisted living residencies" OR "assisted living community" OR "assisted living communities" OR "skilled nursing facility" OR "skilled nursing facilities" OR "skilled nursing residence" OR "skilled nursing residences" OR "skilled nursing residency" OR "skilled nursing residencies" OR  "longterm care home" OR "longterm care homes" OR "longterm care facility" OR "longterm care facilities" OR "longterm care residence" OR "longterm care residences" OR "longterm care residency" OR "longterm care residencies" OR "longterm care resident" OR "longterm care residents" OR "long-term care resident" OR "long-term care residents" OR "long-term care home" OR "long-term care homes" OR "long-term care facility" OR "long-term care facilities" OR "long-term care residence" OR "long-term care residences" OR "long-term care  residency" OR "long-term care residencies" OR "longterm care residence" OR "longterm care residences" OR "longterm care residency" OR "longterm care residencies" OR "convalescent home" OR "convalescent homes" OR "convalescent hospital" OR "convalescent hospitals" OR "convalescent facility" OR "convalescent facilities" OR "convalescent residence" OR "convalescent residences" OR "convalescent residency" OR "convalescent residencies" OR "retirement facility" OR "retirement facilities" OR "retirement home" OR "retirement homes"  OR "retirement residence" OR "retirement residences" OR "retirement residency" OR "retirement residencies" OR "rest home" OR "rest homes" OR "residential care home" OR "residential care homes" OR "residential care facility" OR "residential care facilities" OR "home of the aged" OR "homes of the aged" OR "extended care facility" OR "extended care facilities" OR "extended care home" OR "extended care homes" OR "old age home" OR "old age homes" OR "old age residence" OR "old age residences" OR "old age residency" OR "old age residencies" OR "old peoples home" OR "old people home" OR "old people’s home" OR "old people homes" OR "old peoples homes" OR  "old people’s homes" OR "old people residence" OR "old people residences" OR "old people residency" OR "old people residencies" OR "old peoples residence" OR "old peoples residences" OR "old peoples residency" OR "old peoples residencies" OR "old people’s residence" OR "old people’s residences" OR "old people’s residency" OR "old people’s residencies" OR "charitable home" OR "charitable homes" OR "charitable facility" OR "charitable facilities" |
| --- | --- |

### World Health Organization COVID-19 Global literature on coronavirus disease

| 1 | "nursing home" OR "nursing homes" OR "care home" OR "care homes" OR "nursing residence" OR "nursing residences" OR "nursing residency" OR "nursing residencies" OR "nursing care facility" OR "nursing care facilities" OR "nursing care home" OR "nursing care homes" OR "nursing care residence" OR "nursing care residences" OR "nursing residency" OR "nursing residencies" OR "senior citizen home" OR "senior citizens home" OR "senior citizen homes" OR "senior citizens homes" OR "senior citizen facility" OR "senior citizen facilities" OR "senior citizens facility" OR "senior citizens facilities" OR "senior citizen residence" OR "senior citizen residences" OR  "senior citizen residency" OR "senior citizen residencies" OR "senior citizens residence" OR "senior citizens residences" OR "senior citizens residency" OR "senior citizens residencies" OR "assisted living facility" OR "assisted living facilities" OR "assisted living home" OR "assisted living homes" OR "assisted living residence" OR "assisted living residences" OR "assisted living residency" OR "assisted living residencies" OR "assisted living community" OR "assisted living communities" OR "skilled nursing facility" OR "skilled nursing facilities" OR "skilled nursing residence" OR "skilled nursing residences" OR "skilled nursing residency" OR "skilled nursing residencies" OR  "longterm care home" OR "longterm care homes" OR "longterm care facility" OR "longterm care facilities" OR "longterm care residence" OR "longterm care residences" OR "longterm care residency" OR "longterm care residencies" OR "longterm care resident" OR "longterm care residents" OR "long-term care resident" OR "long-term care residents" OR "long-term care home" OR "long-term care homes" OR "long-term care facility" OR "long-term care facilities" OR "long-term care residence" OR "long-term care residences" OR "long-term care  residency" OR "long-term care residencies" OR "longterm care residence" OR "longterm care residences" OR "longterm care residency" OR "longterm care residencies" OR "convalescent home" OR "convalescent homes" OR "convalescent hospital" OR "convalescent hospitals" OR "convalescent facility" OR "convalescent facilities" OR "convalescent residence" OR "convalescent residences" OR "convalescent residency" OR "convalescent residencies" OR "retirement facility" OR "retirement facilities" OR "retirement home" OR "retirement homes"  OR "retirement residence" OR "retirement residences" OR "retirement residency" OR "retirement residencies" OR "rest home" OR "rest homes" OR "residential care home" OR "residential care homes" OR "residential care facility" OR "residential care facilities" OR "home of the aged" OR "homes of the aged" OR "extended care facility" OR "extended care facilities" OR "extended care home" OR "extended care homes" OR "old age home" OR "old age homes" OR "old age residence" OR "old age residences" OR "old age residency" OR "old age residencies" OR "old peoples home" OR "old people home" OR "old people’s home" OR "old people homes" OR "old peoples homes" OR  "old people’s homes" OR "old people residence" OR "old people residences" OR "old people residency" OR "old people residencies" OR "old peoples residence" OR "old peoples residences" OR "old peoples residency" OR "old peoples residencies" OR "old people’s residence" OR "old people’s residences" OR "old people’s residency" OR "old people’s residencies" OR "charitable home" OR "charitable homes" OR "charitable facility" OR "charitable facilities" |
| --- | --- |

### CINAHL EBSCO

| ## | Query |
| --- | --- |
| S1 | (MM "SARS-CoV-2") OR (MM "Coronavirus") OR (MH "COVID-19+") OR (MM "COVID-19 Pandemic") |
| S2 | AB ( "SARS-CoV-2" OR "SARS-CoV2" OR "SARSCoV-2" OR SARSCoV2 OR "SARS-CoV*" OR SARSCoV* OR "severe acute respiratory syndrome 2" OR "severe acute respiratory syndrome cov*" OR "Covid-19" OR Covid19* OR Covid OR nCoV* OR 2019nCoV* OR 19nCoV* OR "HCoV-19" OR coronavirus* OR "corona virus*" ) AND TI ( "SARS-CoV-2" OR "SARS-CoV2" OR "SARSCoV-2" OR SARSCoV2 OR "SARS-CoV*" OR SARSCoV* OR "severe acute respiratory syndrome 2" OR "severe acute respiratory syndrome cov*" OR "Covid-19" OR Covid19* OR Covid OR nCoV* OR 2019nCoV* OR 19nCoV* OR "HCoV-19" OR coronavirus* OR "corona virus*" ) |
| S3 | AB ( nursing home* or care home* or nursing residen* or nursing care facilit* or nursing care home* or nursing care residen* or senior citizen* home* or senior citizen* faclit* or senior citizen* residen* or assisted living facilit* or assisted living home* or assisted living residen* or assisted living communit* or skilled nursing facilit* or skilled nursing home* or skilled nursing residen* or longterm care home* or longterm care facilit* or longterm care residen* or long-term care home* or long-term care facilit* or long-term care residen* or convalescent home* or convalescent hospital* or convalescent facilit* or convalescent residen* or retirement facilit* or retirement home* or retirement residen* or rest home* or Residential care home* or Residential care facilit* or home of the aged or homes of the aged or extended care facilit* or extended care home* or old age home* or old age residen* or old people* home* or old people* residen* or LTCF or charitable hom* or charitable facilit* ) AND TI ( nursing home* or care home* or nursing residen* or nursing care facilit* or nursing care home* or nursing care residen* or senior citizen* home* or senior citizen* faclit* or senior citizen* residen* or assisted living facilit* or assisted living home* or assisted living residen* or assisted living communit* or skilled nursing facilit* or skilled nursing home* or skilled nursing residen* or longterm care home* or longterm care facilit* or longterm care residen* or long-term care home* or long-term care facilit* or long-term care residen* or convalescent home* or convalescent hospital* or convalescent facilit* or convalescent residen* or retirement facilit* or retirement home* or retirement residen* or rest home* or Residential care home* or Residential care facilit* or home of the aged or homes of the aged or extended care facilit* or extended care home* or old age home* or old age residen* or old people* home* or old people* residen* or LTCF or charitable hom* or charitable facilit* ) |
| S4 | AB ((elder* or senior*or aged or "old age" or "old people" or "old person*") N3 (nursing or "long-term care" or "LTC" or "long term care") N3 (home or homes or hous* or residenc* or facilit* or hospital*)) |
| S5 | (MM "Residential Facilities") OR (MH "Nursing Homes+") OR (MM "Nursing Home Personnel") OR (MM "Nursing Home Patients") |
| S6 | S1 OR S2 |
| S7 | (S3 OR S4 OR S5) |
| S8 | S6 AND S7 |

## Not COVID-19 specific electronic searches

The second component was based on database searches in Medline (Ovid), Embase(Ovid), and CINAHL EBESCO. The search strategy consisted of a combination of search terms related to LTCFs, terms related to respiratory tract infections and/or the pathogens of interest, as well as a set of terms related to the NPIs of interest. The search terms related to the LTCF-setting were based on the search strategy developed for the Cochrane SARS-CoV-2 LTCF review (1) while the outcome and intervention-related terms were developed by expanding on the search strategy of the Cochrane review on “physical interventions to interrupt or reduce the spread of respiratory viruses” (8).

### Medline (Ovid)

| 1 | residential facilities/ or assisted living facilities/ or group homes/ or exp homes for the aged/ or exp nursing homes/ |
| --- | --- |
| 2 | "nursing home*".ti,ab,kw. |
| 3 | "care home*".ti,ab,kw. |
| 4 | ("long-term care facilit*" or "longterm care facilit*" or LTCF).ti,ab,kw. |
| 5 | ("skilled nursing facilit*" or "skilled nursing home*" or "skilled nursing residenc*").ti,ab,kw. |
| 6 | ("nursing care facilit*" or "nursing care home*" or "nursing care residenc*").ti,ab,kw. |
| 7 | ("senior citizen* home*" or "senior citizen* facilit*" or "senior citizen* residenc*").ti,ab,kw. |
| 8 | ("assisted living facilit*" or "assisted living home*" or "assisted living residenc*" or "assisted living communit*").ti,ab,kw. |
| 9 | ("longterm care home*" or "longterm care residen*" or "long-term care home*" or "long-term care residen*").ti,ab,kw. |
| 10 | "nursing residenc*".ti,ab,kw. |
| 11 | ("convalescent home*" or "convalescent hospital*" or "convalescent facilit*" or "convalescent residenc*").ti,ab,kw. |
| 12 | ("retirement facilit*" or "retirement home*" or "retirement residenc*").ti,ab,kw. |
| 13 | ("extended care facilit*" or "extended care home*").ti,ab,kw. |
| 14 | ("home of the aged" or "homes of the aged").ti,ab,kw. |
| 15 | ("old age home*" or "old age residenc*" or "old people* home*" or "old people* residenc*").ti,ab,kw. |
| 16 | ("charitable hom*" or "charitable facilit*").ti,ab,kw. |
| 17 | ("rest home*" or "residential care home*" or "residential care facilit*").ti,ab,kw. |
| 18 | 2 or 3 or 4 or 5 or 6 or 7 or 8 or 9 or 10 or 11 or 12 or 13 or 14 or 15 or 16 or 17 |
| 19 | 1 or 18 |
| 20 | respiratory tract infections/ or exp bronchitis/ or bronchiolitis/ or laryngitis/ or pharyngitis/ or rhinitis/ or supraglottitis/ or tracheitis/ or nasopharyngitis/ or pneumonia/ or bronchopneumonia/ or healthcare-associated pneumonia/ or pneumonia, viral/ or rhinitis/ or tracheitis/ or Bronchiolitis, Viral/ or Infectious bronchitis virus/ |
| 21 | ((respiratory or airway* or lung* or airborne or Cough* or Sneez* or droplet* or aerosol* or pneumonia or bronchopneumonia or bronchitis or bronchiolitis) adj5 (outbreak* or infection* or transmission* or illness* or epidemic* or pandemic*)).ti,ab,kw. |
| 22 | exp Influenza, Human/ or (influenza or influenzas or flu or flus or H1N1 or H2N2 or H3N2 or H5N1 or H7N7 or H1N2 or H9N2 or H7N2 or H7N3 or H10N7).ti,ab,kw. |
| 23 | respiratory syncytial viruses/ or exp respiratory syncytial virus, human/ or ("respiratory syncytial virus" or RSV).ti,ab,kw. |
| 24 | ("common cold" or colds or coryza).ti,ab,kw. or common cold/ |
| 25 | parainfluenza*.ti,ab,kw. |
| 26 | paramyxoviridae infections/ or exp henipavirus infections/ or exp pneumovirus infections/ or exp respirovirus infections/ or exp rubulavirus infections/ or exp orthomyxoviridae infections/ or picornaviridae infections/ or enterovirus infections/ or exp coxsackievirus infections/ or exp echovirus infections/ |
| 27 | ("Middle East respiratory syndrome" or MERS).ti,ab,kw. |
| 28 | exp Severe Acute Respiratory Syndrome/ or "severe acute respiratory syndrome".ti,ab,kw. or SARS.ti,ab,kw. |
| 29 | exp Orthomyxoviridae/ or orthomyxovir*.ti,ab,kw. |
| 30 | exp Coronavirus/ or (Coronavir* or Alphacoronavir* or betacoronavir*).ti,ab,kw. |
| 31 | exp paramyxovirinae/ or exp Paramyxoviridae Infections/ or (paramyxovir* or Morbillivir* or measles or respirovir* or henipavir* or rubulavir* or mumps or hendra* or nipah*).ti,ab,kw. |
| 32 | exp Pneumovirinae/ or (pneumovir* or metapneumovir* or orthopneumovir*).ti,ab,kw. |
| 33 | exp Picornaviridae/ or exp Picornaviridae Infections/ or (picornavir* or enterovir* or rhinovir* or parechovir* or Echovir*).ti,ab,kw. |
| 34 | 20 or 21 or 22 or 23 or 24 or 25 or 26 or 27 or 28 or 29 or 30 or 31 or 32 or 33 |
| 35 | communicable disease control/ or infection control/ or Primary Prevention/ or Universal Precautions/ |
| 36 | (("non-pharmacologic*" or nonpharmacologic* or protect* or prevent* or mitigat* or control* or reduce* or contain* or limit) adj5 (measure* or intervention* or strategy or strategies)).ti,ab,kw. |
| 37 | ((outbreak* or transmission* or infection* or communicable* or droplet* or aerosol*) adj5 (protect* or prevent* or mitigat* or control* or reduce* or contain* or precaution* or regulat*)).ti,ab,kw. |
| 38 | 35 or 36 or 37 |
| 39 | ((readmission* or admission* or admit* or visit* or entry or entries or entrance* or access* or traffic*) adj5 (restrict* or reduce* or limit* or stop* or prohibit* or control*)).ti,ab,kw. |
| 40 | 39 |
| 41 | exp mass screening/ or anonymous testing/ or exp Population Surveillance/ or Point-of-Care Testing/ or self-testing/ or (testing or screenin* or monitor* or surveillance*).ti,ab,kw. |
| 42 | (((early or rapid*) adj5 (detect* or diagnos* or identif*)) or ((routin* or periodic* or regular*) adj5 test*)).ti,ab,kw. or early diagnosis/ |
| 43 | Body Temperature/ or ((temperature or fever) adj5 (check* or measur* or monitor*)).ti,ab,kw. |
| 44 | ((diagnos* or detect* or identif*) adj4 (asymptomatic* or presymptomatic* or "pre‐symptomatic*")).ti,ab,kw. |
| 45 | 41 or 42 or 43 or 44 |
| 46 | exp Respiratory Protective Devices/ or masks/ or (mask* or facemask* or respirator* or N95 or FFP or FFP2 or FFP3).ti,ab,kw. |
| 47 | protective devices/ or exp personal protective equipment/ or ("protective equipment*" or "protective gown*" or PPE or "barrier nursing").ti,ab,kw. |
| 48 | eye protective device/ or (Glasses or Goggle* or "Eyeprotection" or Faceshield* or visor*).ti,ab,kw. |
| 49 | exp Gloves, Protective/ or (glove or gloves).ti,ab,kw. |
| 50 | hand hygiene/ or exp hand disinfection/ or (handwashing or "hand-washing" or "respiratory etiquette").ti,ab,kw. |
| 51 | ((Hand or hands or Alcohol) adj3 (wash or Washing or Cleansing or Rinses or hygiene or rub or Rubbing or sanitizer or sanitiser or cleanser or disinfected or Disinfectant or Disinfect or antiseptic or virucid)).ti,ab,kw. |
| 52 | Hygiene/ or sanitation/ or antisepsis/ or sterilization/ or disinfection/ or Disinfectants/ or (hygiene* or sanitize* or sanitise* or disinfect* or desinfect* or clean* or sterilize* or decontaminat*).ti,ab,kw. |
| 53 | 46 or 47 or 48 or 49 or 50 or 51 or 52 |
| 54 | exp physical distancing/ or ((contact* or interaction* or group* or activit*) adj5 (reduc* or limit* or prohibit* or control* or regulat* or precaution* or stop*)).ti,ab,kw. or distancing.ti,ab,kw. |
| 55 | (cohorting* or compartment* or zone or zones or zoning).ti,ab,kw. |
| 56 | exp medical leave/ or (("sick" or "medical") adj2 ("leave" or "pay")).ti,ab,kw. or (Presenteeism or "working while sick" or "working while contagious" or "working while infectious").ti,ab,kw. |
| 57 | 54 or 55 or 56 |
| 58 | exp Air Filters/ or (ventilat* or "air quality" or "air filter" or "air filtration").ti,ab,kw. |
| 59 | exp Built Environment/ or exp Environment Design/ or (barrier* or engineer*).ti,ab,kw. |
| 60 | 58 or 59 |
| 61 | Patient Isolation/ or Quarantine/ or (quarantin* or isolat* or confine*).ti,ab,kw. |
| 62 | exp Contact Tracing/ or (contact tracing* or contact examination*).ti,ab,kw. |
| 63 | 61 or 62 |
| 64 | 38 or 40 or 45 or 53 or 57 or 60 or 63 |
| 65 | 19 and 34 and 64 |
| 66 | Animal/ not human/ |
| 67 | 65 not 66 |
| 68 | limit 67 to editorial |
| 69 | 67 not 68 |

### Embase(Ovid)

| 1 | ("nursing home*" or "care home*" or "nursing residenc*" or "nursing care facilit*" or "nursing care home*" or "nursing care residenc*").ti,ab,kw. |
| --- | --- |
| 2 | ("longterm care facilit*" or "long-term care facilit*" or LTCF or "longterm care home*" or "longterm care residen*" or "long-term care home*" or "long-term care residen*").ti,ab,kw. |
| 3 | ("assisted living facilit*" or "assisted living home*" or "assisted living residenc*" or "assisted living communit*").ti,ab,kw. |
| 4 | ("skilled nursing facilit*" or "skilled nursing home*" or "skilled nursing residenc*").ti,ab,kw. |
| 5 | ("senior citizen* home*" or "senior citizen* facilit*" or "senior citizen* residenc*").ti,ab,kw. |
| 6 | ("convalescent home*" or "convalescent hospital*" or "convalescent facilit*" or "convalescent residenc*").ti,ab,kw. |
| 7 | ("retirement facilit*" or "retirement home*" or "retirement residenc*").ti,ab,kw. |
| 8 | ("rest home*" or "residential care home*" or "residential care facilit*").ti,ab,kw. |
| 9 | ("home of the aged" or "homes of the aged").ti,ab,kw. |
| 10 | ("extended care facilit*" or "extended care home*").ti,ab,kw. |
| 11 | ("old age home*" or "old age residenc*" or "old people* home*" or "old people* residenc*").ti,ab,kw. |
| 12 | ("charitable hom*" or "charitable facilit*").ti,ab,kw. |
| 13 | exp nursing home/ or exp nursing home patient/ or exp nursing home personnel/ or exp residential home/ or exp home for the aged/ |
| 14 | 1 or 2 or 3 or 4 or 5 or 6 or 7 or 8 or 9 or 10 or 11 or 12 or 13 |
| 15 | exp viral respiratory tract infection/ or respiratory tract infection/ |
| 16 | exp viral upper respiratory tract infection/ or upper respiratory tract infection/ or laryngotracheobronchitis/ or nose infection/ or rhinopharyngitis/ |
| 17 | exp viral lower respiratory tract infection/ or exp virus pneumonia/ or pneumonia/ or bronchopneumonia/ or infectious pneumonia/ or interstitial pneumonia/ or lung infiltrate/ or health care associated pneumonia/ or bornholm disease/ or chest infection/ or lung infection/ or hantavirus pulmonary syndrome/ or viral bronchiolitis/ |
| 18 | ((respiratory or airway* or lung* or airborne or Cough* or Sneez* or droplet* or aerosol* or pneumonia or bronchopneumonia or bronchitis or bronchiolitis) adj5 (outbreak* or infection* or transmission* or illness* or epidemic* or pandemic*)).ti,ab,kw. |
| 19 | exp influenza/ or (influenza or influenzas or flu or flus or H1N1 or H2N2 or H3N2 or H5N1 or H7N7 or H1N2 or H9N2 or H7N2 or H7N3 or H10N7).ti,ab,kw. |
| 20 | ("respiratory syncytial virus" or RSV).ti,ab,kw. or exp respiratory syncytial virus infection/ |
| 21 | ("common cold" or colds or coryza).ti,ab,kw. or common cold/ |
| 22 | exp Parainfluenza virus infection/ or parainfluenza.ti,ab,kw. |
| 23 | exp severe acute respiratory syndrome/ or "severe acute respiratory syndrome".ti,ab,kw. or SARS.ti,ab,kw. |
| 24 | ("Middle East respiratory syndrome" or MERS).ti,ab,kw. or exp Middle East respiratory syndrome/ |
| 25 | exp orthomyxovirus infection/ or orthomyxovirus*.ti,ab,kw. |
| 26 | exp Coronavirus/ or exp Coronavirus infection/ or (Coronavir* or Alphacoronavir* or betacoronavir*).ti,ab,kw. |
| 27 | exp orthomyxovirus infection/ or orthomyxovir*.ti,ab,kw. |
| 28 | exp paramyxovirinae/ or exp paramyxovirus infection/ or (paramyxovir* or Morbillivir* or measles or respirovir* or henipavir* or rubulavir* or mumps or hendra* or nipah*).ti,ab,kw. |
| 29 | exp picornaviridae/ or exp picornavirus infection/ or (picornavir* or enterovir* or rhinovir* or parechovir* or Echovir*).ti,ab,kw. |
| 30 | exp pneumovirus/ or exp pneumovirus infection/ or (pneumovir* or metapneumovir* or orthopneumovir*).ti,ab,kw. |
| 31 | 15 or 16 or 17 or 18 or 19 or 20 or 21 or 22 or 23 or 24 or 25 or 26 or 27 or 28 or 29 or 30 |
| 32 | exp infection control/ or exp communicable disease control/ or exp infection prevention/ |
| 33 | prevention/ or primary prevention/ or "prevention and control"/ or disease control/ or control/ or control strategy/ or universal precaution/ |
| 34 | (("non-pharmacologic*" or nonpharmacologic* or protect* or prevent* or mitigat* or control* or reduce* or contain* or limit) adj5 (measure* or intervention* or strategy or strategies)).ti,ab,kw. |
| 35 | ((outbreak* or transmission* or infection* or communicable* or droplet* or aerosol*) adj5 (protect* or prevent* or mitigat* or control* or reduce* or contain* or precaution* or regulat*)).ti,ab,kw. |
| 36 | 32 or 33 or 34 or 35 |
| 37 | ((readmission* or admission* or admit* or visit* or entry or entries or entrance* or access* or traffic*) adj5 (restrict* or reduce* or limit* or stop* or prohibit* or control*)).ti,ab,kw. |
| 38 | 37 |
| 39 | screening test/ or disease surveillance/ or mass screening/ or "mandatory testing"/ or (testing or screenin* or monitor* or surveillance*).ti,ab,kw. |
| 40 | (((early or rapid*) adj5 (detect* or diagnos* or identif*)) or ((routin* or periodic* or regular*) adj5 test*)).ti,ab,kw. or early diagnosis/ |
| 41 | Body Temperature/ or ((temperature or fever) adj4 (check* or measur* or monitor*)).ti,ab,kw. |
| 42 | ((diagnos* or detect* or identif*) adj4 (asymptomatic* or presymptomatic* or "pre‐symptomatic*")).ti,ab,kw. |
| 43 | 39 or 40 or 41 or 42 |
| 44 | exp respiratory protection/ or exp mask/ or (mask* or facemask* or respirator* or N95 or FFP or FFP2 or FFP3 or "barrier nursing").ti,ab,kw. |
| 45 | exp protective equipment/ or exp protective clothing/ or ("protective equipment*" or "protective gown*" or PPE).ti,ab,kw. |
| 46 | eye protective device/ or (Glasses or Goggle* or "Eyeprotection" or Faceshield* or visor*).ti,ab,kw. |
| 47 | exp protective glove/ or (glove or gloves).ti,ab,kw. |
| 48 | exp hand washing/ or exp hand disinfection/ or (handwashing or "hand-washing" or "respiratory etiquette").ti,ab,kw. |
| 49 | ((Hand or hands or Alcohol) adj3 (wash or Washing or Cleansing or Rinses or hygiene or rub or Rubbing or sanitizer or sanitiser or cleanser or disinfected or Disinfectant or Disinfect or antiseptic or virucid)).ti,ab,kw. |
| 50 | hygiene/ or exp hospital hygiene/ or (hygiene* or sanitize* or sanitise* or disinfect* or desinfect* or clean* or sterilize* or decontaminat*).ti,ab,kw. |
| 51 | 44 or 45 or 46 or 47 or 48 or 49 or 50 |
| 52 | exp social distancing/ or ((contact* or interaction* or group* or activit*) adj5 (reduc* or limit* or prohibit* or control* or regulat* or precaution* or stop*)).ti,ab,kw. or distancing.ti,ab,kw. |
| 53 | (cohorting* or compartment* or zone or zones or zoning).ti,ab,kw. |
| 54 | exp medical leave/ or (("sick" or "medical") adj2 ("leave" or "pay")).ti,ab,kw. or (Presenteeism or "working while sick" or "working while contagious" or "working while infectious").ti,ab,kw. |
| 55 | 52 or 53 or 54 |
| 56 | exp air conditioning/ or exp air quality/ or exp air filter/ or (ventilat* or "air quality" or "air filter" or "air filtration").ti,ab,kw. |
| 57 | exp "construction work and architectural phenomena"/ or exp "environmental planning"/ or (barrier* or engineer*).ti,ab,kw. |
| 58 | 56 or 57 |
| 59 | exp quarantine/ or exp isolation/ or exp patient isolation/ or (quarantin* or isolat* or confine*).ti,ab,kw. |
| 60 | contact examination/ or (contact tracing* or contact examination*).ti,ab,kw. |
| 61 | 59 or 60 |
| 62 | 36 or 38 or 43 or 51 or 55 or 58 or 61 |
| 63 | 14 and 31 and 62 |
| 64 | Animal/ not human/ |
| 65 | 63 not 64 |
| 66 | (("case study" or "case series") and (patient* or resident*)).ti. |
| 67 | 65 not 66 |

### CINAHL EBSCO

| # | Query |
| --- | --- |
| S01 | TI ( "nursing home*" or "care home*" or "nursing residenc*" or "nursing care facilit*" or "nursing care home*" or "nursing care residenc*" or "senior citizen* home*" or "senior citizen* facilit*" or "senior citizen* residenc*" or "assisted living facilit*" or "assisted living home*" or "assisted living residenc*" or "assisted living communit*" or "skilled nursing facilit*" or "skilled nursing home*" or "skilled nursing residenc*" or "longterm care home*" or "longterm care facilit*" or "longterm care residen*" or "long-term care home*" or "long-term care facilit*" or "long-term care residen*" or "convalescent home*" or "convalescent hospital*" or "convalescent facilit*" or "convalescent residenc*" or "retirement facilit*" or "retirement home*" or "retirement residenc*" or "rest home*" or "residential care home*" or "residential care facilit*" or "home of the aged" or "homes of the aged" or "extended care facilit*" or "extended care home*" or "old age home*" or "old age residenc*" or "old people* home*" or "old people* residenc*" or LTCF or "charitable hom*" or "charitable facilit*" ) OR AB ( "nursing home*" or "care home*" or "nursing residenc*" or "nursing care facilit*" or "nursing care home*" or "nursing care residenc*" or "senior citizen* home*" or "senior citizen* facilit*" or "senior citizen* residenc*" or "assisted living facilit*" or "assisted living home*" or "assisted living residenc*" or "assisted living communit*" or "skilled nursing facilit*" or "skilled nursing home*" or "skilled nursing residenc*" or "longterm care home*" or "longterm care facilit*" or "longterm care residen*" or "long-term care home*" or "long-term care facilit*" or "long-term care residen*" or "convalescent home*" or "convalescent hospital*" or "convalescent facilit*" or "convalescent residenc*" or "retirement facilit*" or "retirement home*" or "retirement residenc*" or "rest home*" or "residential care home*" or "residential care facilit*" or "home of the aged" or "homes of the aged" or "extended care facilit*" or "extended care home*" or "old age home*" or "old age residenc*" or "old people* home*" or "old people* residenc*" or LTCF or "charitable hom*" or "charitable facilit*" ) |
| S02 | (MM "Nursing Home Personnel") OR (MM "Nursing Home Patients") OR (MM "Home Nursing, Professional") OR (MH "Nursing Homes+") |
| S03 | (S1 OR S2) |
| S04 | (MM "Respiratory Tract Infections") OR (MM "Bronchitis") OR (MH "Laryngitis+") OR (MM "Pharyngitis") OR (MM "Pneumonia") OR (MM "Bronchopneumonia") OR (MM "Healthcare-Associated Pneumonia") OR (MM "Pneumonia, Viral") OR (MM "Rhinitis") OR (MM "Rhinosinusitis") OR (MM "Sinusitis") OR (MM "Tonsillitis") |
| S05 | TI ( (respiratory or airway* or lung* or airborne or Cough* or Sneez* or droplet* or aerosol* or pneumonia or bronchopneumonia or bronchitis or bronchiolitis) N8 (outbreak* or infection* or transmission* or illness* or epidemic* or pandemic*) ) OR AB ( (respiratory or airway* or lung* or airborne or Cough* or Sneez* or droplet* or aerosol* or pneumonia or bronchopneumonia or bronchitis or bronchiolitis) N8 (outbreak* or infection* or transmission* or illness* or epidemic* or pandemic*) ) |
| S06 | TI ( influenza or influenzas or flu or flus or H1N1 or H2N2 or H3N2 or H5N1 or H7N7 or H1N2 or H9N2 or H7N2 or H7N3 or H10N7) OR AB ( influenza or influenzas or flu or flus or H1N1 or H2N2 or H3N2 or H5N1 or H7N7 or H1N2 or H9N2 or H7N2 or H7N3 or H10N7) OR (MH "Influenza+") |
| S07 | TI ( "respiratory syncytial virus" or RSV ) OR AB ( "respiratory syncytial virus" or RSV ) OR ( (MM "Respiratory Syncytial Virus Infections") OR (MM "Respiratory Syncytial Viruses") ) |
| S08 | TI ( "common cold" or colds or coryza ) OR AB ( "common cold" or colds or coryza ) OR (MM "Common Cold") |
| S09 | TI parainfluenza* OR AB parainfluenza* |
| S10 | TI ( "Severe Acute Respiratory Syndrome" OR SARS ) OR AB ( "Severe Acute Respiratory Syndrome" OR SARS ) AND ( (MM "SARS Virus") OR (MM "Severe Acute Respiratory Syndrome") ) |
| S11 | TI ( "Middle East respiratory syndrome" or MERS ) OR AB ( "Middle East respiratory syndrome" or MERS ) AND ( (MM "Middle East Respiratory Syndrome") OR (MM "Middle East Respiratory Syndrome Coronavirus") ) |
| S12 | TI Coronavirus* OR AB Coronavirus* AND (MH "Coronavirus+") |
| S13 | TI Orthomyxovir* OR AB Orthomyxovir* AND (MH "Orthomyxoviridae+") |
| S14 | TI ( Respirovir* OR Henipavir* OR Hantavir* OR nipah* OR respirovir* OR rubulavir* or mumps OR measles OR morbillivir* OR paramyxovir* OR echovir* OR enterovir* OR picornavir* OR pneumovir* OR metapneumovir* OR orthopneumovir* OR rhinovir* OR parechovir* ) OR AB ( Respirovir* OR Henipavir* OR Hantavir* OR nipah* OR respirovir* OR rubulavir* or mumps OR measles OR morbillivir* OR paramyxovir* OR echovir* OR enterovir* OR picornavir* OR pneumovir* OR metapneumovir* OR orthopneumovir* OR rhinovir* OR parechovir* ) OR ( (MH "Paramyxovirus Infections+") or (MH "Paramyxoviruses+") (MM "Echovirus Infections") OR (MM "Enterovirus Infections") OR (MH "Picornavirus Infections+") ) |
| S15 | S4 OR S5 OR S6 OR S7 OR S8 OR S9 OR S10 OR S11 OR S12 OR S13 OR S14 |
| S16 | (MM "Infection Control") |
| S17 | TI ( ("non-pharmacologic*" or nonpharmacologic* or protect* or prevent* or mitigat* or control* or reduce* or contain* or limit) N6 (measure* or intervention* or strategy or strategies) ) OR AB ( ("non-pharmacologic*" or nonpharmacologic* or protect* or prevent* or mitigat* or control* or reduce* or contain* or limit) N6 (measure* or intervention* or strategy or strategies) ) |
| S18 | TI ( (outbreak* or transmission* or infection* or communicable* or droplet* or aerosol*) N6 (protect* or prevent* or mitigat* or control* or reduce* or contain* or precaution* or regulat*) ) OR AB ( (outbreak* or transmission* or infection* or communicable* or droplet* or aerosol*) N6 (protect* or prevent* or mitigat* or control* or reduce* or contain* or precaution* or regulat*) ) |
| S19 | TI ( (readmission* or admission* or admit* or visit* or entry or entries or entrance* or access* or traffic*) N6 (restrict* or reduce* or limit* or stop* or prohibit* or control*) ) OR AB ( (readmission* or admission* or admit* or visit* or entry or entries or entrance* or access* or traffic*) N6 (restrict* or reduce* or limit* or stop* or prohibit* or control*) ) |
| S20 | TI (testing or screenin* or monitor* or surveillance* ) OR AB (testing or screenin* or monitor* or surveillance* ) OR ( (MM "Mandatory Reporting") OR (MM "Mandatory Testing") OR (MM "Disease Surveillance") ) |
| S21 | AB ((temperature or fever or symptom*) N4 (check* or measur* or monitor*)) OR TI ((temperature or fever or symptom*) N4 (check* or measur* or monitor*)) |
| S22 | TI ( (early or rapid* or routin*) N6 (detect* or diagnos* or identif* or test*) ) OR AB ( (early or rapid* or routin*) N6 (detect* or diagnos* or identif* or test*) ) |
| S23 | TI ( (diagnos* or detect* or identif*) N4 (asymptomatic* or presymptomatic* or "pre‐symptomatic*") ) OR AB ( (diagnos* or detect* or identif*) N4 (asymptomatic* or presymptomatic* or "pre‐symptomatic*") ) |
| S24 | S20 OR S21 OR S22 OR S23 |
| S25 | TI ( mask* or facemask* or respirator* or N95 OR FFP OR FFP2 OR FFP3 ) OR AB ( mask* or facemask* or respirator* or N95 OR FFP OR FFP2 OR FFP3 ) OR ( (MH "Respiratory Protective Devices+") OR (MM "N95 Respirators") OR (MM "Masks") ) |
| S26 | (MM "Protective Devices") OR (MM "Personal Protective Equipment") OR (MM "Protective Clothing") OR AB ( "protective equipment*" OR "protective gown*" or "protective cloth*" OR PPE ) OR TI ( "protective equipment*" or "protective cloth*" OR "protective gown*" OR PPE ) |
| S27 | TI ( Glasses or Goggle* or "Eyeprotection" or Faceshield* or visor*) OR AB ( Glasses or Goggle* or "Eyeprotection" or Faceshield* ) OR (MM "eye protective device" or visor*) |
| S28 | (MM "Gloves") OR AB ("glove*") OR TI ("glove*") |
| S29 | TI ( (Hand or hands or Alcohol) N3 (wash or Washing or Cleansing or Rinses or hygiene or rub or Rubbing or sanitizer or sanitiser or cleanser or disinfected or Disinfectant or Disinfect or antiseptic or virucid) ) OR AB ( (Hand or hands or Alcohol) N3 (wash or Washing or Cleansing or Rinses or hygiene or rub or Rubbing or sanitizer or sanitiser or cleanser or disinfected or Disinfectant or Disinfect or antiseptic or virucid) ) OR (MM "Handwashing") OR TI ("handwashing" OR "hand-washing") OR AB ("handwashing" OR "hand-washing") |
| S30 | TI ( hygiene* or sanitize* or sanitise* or disinfect* or desinfect* or clean* or sterilize* or decontaminat* ) OR AB ( hygiene* or sanitize* or sanitise* or disinfect* or desinfect* or clean* or sterilize* or decontaminat* ) OR ( (MM "Hygiene") OR (MM "Asepsis") OR (MM "Sterilization and Disinfection") ) |
| S31 | (MM "Universal Precautions") |
| S32 | S25 OR S26 OR S27 OR S28 OR S29 OR S30 OR S31 |
| S33 | TI ( (contact* or interaction* or group* or activit*) N6 (reduc* or limit* or prohibit* or control* or regulat* or precaution*) ) OR AB ( (contact* or interaction* or group* or activit*) N6 (reduc* or limit* or prohibit* or control* or regulat* or precaution*) ) |
| S38 | S33 OR S34 OR S35 OR S36 OR S37 |
| S39 | TI ( ventilat* or "air quality" or "air filter" or "air filtration" or "air quality" ) OR AB ( ventilat* or "air quality" or "air filter" or "air filtration" or "air quality" ) OR (MM "Air Filters") |
| S40 | TI ( barrier* OR construct* ) OR AB ( barrier* OR construct* ) OR ( (MM "Nursing Home Design and Construction") ) |
| S41 | TI ( quarantin* or isolat* or confine* ) OR AB ( quarantin* or isolat* or confine* ) OR ( (MM "Patient Isolation") OR (MM "Quarantine") ) |
| S42 | TI ( "contact tracing*" OR "contact examination*" ) OR AB ( "contact tracing*" OR "contact examination*" ) OR (MM "Contact Tracing") |
| S43 | S39 OR S40 OR S41 OR S42 |
| S44 | S16 OR S17 OR S18 OR S19 OR S24 OR S32 OR S38 OR S43 |
| S45 | S3 AND S15 AND S44 |

# Appendix III: List of reviews used in the reference searches

- Aida Suarez-Gonzalez, Jayeeta Rajagopalan, Gill Livingston, Suvarna Alladi. The effect of Covid-19 isolation measures on the cognition and mental health of people living with dementia: a rapid systematic review of one year of evidence 2021.
- Arias-Casais N, Thiyagarajan JA, Perracini MR, et al. What long-term care interventions have been published between 2010 and 2020? Results of a WHO scoping review identifying long-term care interventions for older people around the world. BMJ Open 2022; 12.
- Bach-Mortensen AM, Verboom B, Movsisyan A, Degli Esposti M. A systematic review of the associations between care home ownership and COVID-19 outbreaks, infections and mortality. Nat Aging 2021; 1(10): 948–61 [https://doi.org/10.1038/s43587-021-00106-7]
- Bach-Mortensen AM, Verboom B, Movsisyan A, Degli Esposti M. Ownership and COVID-19 in care homes for older people: A living systematic review of outbreaks, infections, and mortalities. Medrxiv 2021 [https://doi.org/10.1101/2021.01.28.21250547]
- Benbow WB. COVID-19 in Long-Term Care: The Built Environment Impact on Infection Control. HERD 2022; 15(4): 287–98 [https://doi.org/10.1177/19375867221101897][PMID: 35684993]
- Benzinger P, Kuru S, Keilhauer A, et al. Psychosoziale Auswirkungen der Pandemie auf Pflegekräfte und Bewohner von Pflegeheimen sowie deren Angehörige – Ein systematisches Review. Z Gerontol Geriatr 2021; 54(2): 141–5 [https://doi.org/10.1007/s00391-021-01859-x][PMID: 33624143]
- Beogo I, Sia D, Tchouaket Nguemeleu E, Zhao J, Gagnon M-P, Etowa J. Strengthening Social Capital to Address Isolation and Loneliness in Long-term Care Facilities During the COVID-19 Pandemic: Protocol for a Systematic Review of Research on Information and Communication Technologies. JMIR Res Protoc 2022; 11(3): e36269 [https://doi.org/10.2196/36269][PMID: 35275841]
- Beogo I, Tchouaket EN, Sia D, et al. Promising best practices implemented in long-term care homes during COVID-19 pandemic to address social isolation and loneliness: a scoping review protocol. BMJ Open 2022; 12(1): e053894 [https://doi.org/10.1136/bmjopen-2021-053894][PMID: 34980621]
- Bethell J, Aelick K, Babineau J, et al. Social Connection in Long-Term Care Homes: A Scoping Review of Published Research on the Mental Health Impacts and Potential Strategies During COVID-19. J Am Med Dir Assoc 2021; 22(2): 228-237.e25 [https://doi.org/10.1016/j.jamda.2020.11.025][PMID: 33347846]
- Blum B, Daoud J, Alvarez-Villalba C. Landscape of Isolation: Covid-19 and Geriatric Mental Health. The American Journal of Geriatric Psychiatry 2022; 30(4): S93 [https://doi.org/10.1016/j.jagp.2022.01.224]
- Calcaterra L, Cesari M, Lim WS. Long-Term Care Facilities (LTCFs) During the COVID-19 Pandemic-Lessons from the Asian Approach: A Narrative Review. J Am Med Dir Assoc 2022; 23(3): 399–404 [https://doi.org/10.1016/j.jamda.2022.01.049][PMID: 35085508]
- Daly T, Shanagher D. COVID-19 NURSING HOMES EXPERT PANEL REPORT IMPACT ASSESSMENT. Age and ageing 2021; 50: 1 [https://doi.org/10.1093/ageing/afab219.69]
- Dykgraaf SH, Matenge S, Desborough J, et al. Protecting Nursing Homes and Long-Term Care Facilities From COVID-19: A Rapid Review of International Evidence. J Am Med Dir Assoc 2021; 22(10): 1969–88 [https://doi.org/10.1016/j.jamda.2021.07.027]
- Egunsola O, Hofmeister M, Dowsett LE, Noseworthy T, Clement F. Preventing the Transmission of COVID-19 in Older Adults Aged 60 Years and Above Living in Long-Term Care: Rapid Review Update 2021.
- [Fischer F, Raiber L, Boscher C, Winter MHJ. COVID-19-Schutzmasnahmen in der stationaren Altenpflege. Pflege 2020; 33: 199–206.
- Frazer K, Mitchell L, Stokes D, Lacey E, Crowley E, Kelleher CC. A rapid systematic review of measures to protect older people in long-term care facilities from COVID-19. BMJ Open 2021; 11(10) [https://doi.org/10.1136/BMJOPEN-2020-047012]
- Gmehlin CG, Munoz-Price LS. Coronavirus disease 2019 (COVID-19) in long-term care facilities: A review of epidemiology, clinical presentations, and containment interventions. Infect Control Hosp Epidemiol 2022; 43(4): 504–9 [https://doi.org/10.1017/ice.2020.1292]
- Gordon AL, Logan PA, Jones RG, et al. A systematic mapping review of randomized controlled trials (RCTs) in care homes. BMC Geriatr 2012; 12: 31 [https://doi.org/10.1186/1471-2318-12-31]
- Gould DJ, Moralejo D, Drey N, Chudleigh JH, Taljaard M. Interventions to improve hand hygiene compliance in patient care. Cochrane Database Syst Rev 2017; 9(9): CD005186 [https://doi.org/10.1002/14651858.CD005186.pub4][PMID: 28862335]
- Graverholt B, Forsetlund L, Jamtvedt G. Reducing hospital admissions from nursing homes: a systematic review. BMC Health Serv Res 2014; 14: 36 [https://doi.org/10.1186/1472-6963-14-36]
- Heneghan C, Dietrich M, Brassey J, Jefferson T. Effects of COVID-19 in Care Homes - A Mixed Methods Review. Medrxiv 2022 [https://doi.org/10.1101/2022.04.14.22273903]
- Hocine MN, Temime L. Impact of hand hygiene on the infectious risk in nursing home residents: A systematic review. Am J Infect Control 2015; 43(9): e47-52 [https://doi.org/10.1016/j.ajic.2015.05.043][PMID: 26184767]
- Hugelius K, Harada N, Marutani M. Consequences of visiting restrictions during the COVID-19 pandemic: An integrative review. Int J Nurs Stud 2021; 121: 104000 [https://doi.org/10.1016/j.ijnurstu.2021.104000]
- Jefferson T, Del Mar CB, Dooley L, et al. Physical interventions to interrupt or reduce the spread of respiratory viruses. Cochrane Database of Systematic Reviews 2020; (11) [https://doi.org/10.1002/14651858.CD006207.pub5]
- Jones K, Schnitzler K, Borgstrom E. The implications of COVID-19 on health and social care personnel in long-term care facilities for older people: An international scoping review. Health Soc Care Community 2022 [https://doi.org/10.1111/hsc.13969]
- Koszalinski RS, Olmos B. Communication challenges in social isolation, subjective cognitive decline, and mental health status in older adults: A scoping review (2019-2021). Perspect. psychiatr. care 2022 [https://doi.org/10.1111/ppc.13115]
- Laher Z, Robertson N, Harrad-Hyde F, Jones CR. Prevalence, Predictors, and Experience of Moral Suffering in Nursing and Care Home Staff during the COVID-19 Pandemic: A Mixed-Methods Systematic Review. Int. j. environ. res. public health (Online) 2022; 19(15) [https://doi.org/10.3390/ijerph19159593]
- Lee MH, Lee GA, Lee SH, Park YH. A systematic review on the causes of the transmission and control measures of outbreaks in long-term care facilities: Back to basics of infection control. PLoS One 2020; 15(3): e0229911 [https://doi.org/10.1371/journal.pone.0229911]
- Liljas AEM, Morath LP, Burstrom B, Schon P, Agerholm J. The impact of organisational characteristics of staff and facility on infectious disease outbreaks in care homes: a systematic review. BMC Health Serv Res 2022; 22(1): 339 [https://doi.org/10.1186/s12913-022-07481-w]
- Low LF, Fletcher J, Goodenough B, et al. A Systematic Review of Interventions to Change Staff Care Practices in Order to Improve Resident Outcomes in Nursing Homes. PLoS One 2015; 10(11): e0140711 [https://doi.org/10.1371/journal.pone.0140711]
- National Collaborating Centre for Methods and Tools. What strategies mitigate risk of COVID-19 outbreaks and mortality in long-term care facilities? 2021.
- Olson NL, Albensi BC. Dementia-Friendly "Design": Impact on COVID-19 Death Rates in Long-Term Care Facilities Around the World. J Alzheimers Dis 2021; 81(2): 427–50 [https://doi.org/10.3233/JAD-210017]
- Rainwater-Lovett K, Chun K, Lessler J. Influenza outbreak control practices and the effectiveness of interventions in long-term care facilities: a systematic review. Influenza Other Respir Viruses 2014; 8(1): 74–82 [https://doi.org/10.1111/irv.12203]
- Rios P, Radhakrishnan A, Williams C, et al. Preventing the transmission of COVID-19 and other coronaviruses in older adults aged 60 years and above living in long-term care: a rapid review. Syst Rev 2020; 9(1): 218 [https://doi.org/10.1186/s13643-020-01486-4]
- Rodrigues NG, Han CQY, Su Y, Klainin-Yobas P, Wu XV. Psychological impacts and online interventions of social isolation amongst older adults during COVID-19 pandemic: A scoping review. J Adv Nurs 2022; 78(3): 609–44 [https://doi.org/10.1111/jan.15063]
- Sims S, Harris R, Hussein S, et al. Social Distancing and Isolation Strategies to Prevent and Control the Transmission of COVID-19 and Other Infectious Diseases in Care Homes for Older People: An International Review. Int. j. environ. res. public health (Online) 2022; 19(6) [https://doi.org/10.3390/ijerph19063450]
- Spencer, Llinos Haf Hartfiel Ned Hendry Annie Anthony Bethany Makanjuola Abraham Bray Nathan Hughes Dyfrig Wilkinson Clare Fitzsimmons Deb Edwards Rhiannon Tudor. Have infection control and prevention measures resulted in any adverse outcomes for care home and domiciliary care residents and staff? (preprint) 2022 [https://doi.org/10.1101/2022.05.04.22274657]
- Walsh KA, Broderick N, Ahern S, et al. Effectiveness of rapid antigen testing for screening of asymptomatic individuals to limit the transmission of SARS-CoV-2: A rapid review. Rev Med Virol 2022: e2350-e2350 [https://doi.org/10.1002/rmv.2350]
- Yang H, Rigsby M, Zhu X, Lee C, Ory M. COVID-19 in Long-Term Care Facilities: A Rapid Review of Infection Correlates and Impacts on Mental Health and Behaviors. HERD 2022; 15(3): 277–94 [https://doi.org/10.1177/19375867221092149]

# Appendix IV: Extended Methods section

## Risk of bias assessment

Two review authors independently rated the risk of bias (RoB) of each included study using different tools depending on the study type. For the RoB assessment of RCTs, we applied the Cochrane RoB 2 tool (9) with adapted versions for cluster-RCTs (10). For NRSIs, we used the most recent version of ROBINS-I (11), employing the guidance on the adaption of the tool laid out in the Cochrane Handbook for the RoB assessment of ITS, CBA, and cITS studies (12).

Discrepancies and uncertainties in the assessment were discussed together with a third reviewer from the review team.

Within the assessment, our focus lay on the assessment of the direction of effect (i.e., how high do we consider the risk, that bias would have caused the found direction of effect, while the true effect within the study was either a null-effect or an effect in the opposite direction).

In applying the ROBINS-I, it is recommended to define important confounding factors a-priori. A primary consideration lay on confounding due to underlying characteristics of the LTCF, in the form of an abstract notion of “quality” (e.g., LTCFs which were better managed, had more financial resources, and had more social or political capital to their disposal). Within this rationale, LTCFs with higher levels of “quality” could be more likely to implement protective measures (e.g., due to higher motivation and/or more capacity to invest the necessary resources) while at the same time directly or indirectly affecting the outcomes of interest (e.g., by having more or other baseline infection control measures in place or the structure of the facility leading to a lower likelihood of outbreaks).

Based on the logic model, further important confounding factors which could both be associated with the likelihood of NPIs being implemented and/or the adherence to them as well as to the outcomes of interest include:

- intra-individual risk and protective factors (e.g., age, sex, or health status of residents; e.g., leading to a stronger motivation to protect themselves or to be protected)
- physical or metabolic vulnerability of the residents or staff members for becoming infected (e.g., vaccination rates)
- underlying structural or organizational risk factors for infections/outbreaks in LTCFs (e.g., size of the facility)
- underlying risk factors for infections/outbreaks in LTCFs resulting from non-pharmacological infection prevention measures in place (e.g., regulation of PPE usage)
- epidemiological pressure of the pathogen outside of the facility and the resulting risk of infection of residents, visitors, and staff outside of the facility (e.g., risk of infection of staff in the community resulting from the type and strain of the pathogen and the local 7-day incidence rate)
- factors relating to social practices (e.g., holidays affecting rates of visitors in a time-series analysis)
- testing-related factors (e.g., differences in procedures to detect or identify cases)
- general implementation factors (e.g., differences in how the intervention is implemented across facilities)

We considered co-interventions as further source of bias in NRSIs, if applied differently between intervention and control group. Due to the heterogeneity in the accepted standard care in LTCFs (i.e., rules on the rate of disinfection of high-touch surfaces) as well as infection prevention and control practices varying over time even during pandemic events, it was not possible for us to define relevant co-interventions a-priori. However, we compiled a list with important co-interventions within the team responsible for conducting the RoB assessment for each included study before conducting the RoB assessment (12).

## Minimal threshold for public health relevance

Within this study, we defined the thresholds for the public-health relevance (corresponding to the minimal patient-relevant differences) as any difference from the null. This is done, as in pandemic-like public health emergencies, any intervention which allows for a reduction of infection risk could potentially be relevant.

Accordingly, the focus of the RoB assessment, the narrative synthesis and the graphical display thereof, as well as the assessment of the certainty of evidence was the direction of effect, rather than the effect size. However, we provided information on the effect sizes, to inform decision makers in the balancing process of beneficial and adverse consequences of the intervention.

## Intervention effect of interest

In complex public health interventions, the effective component of an intervention (the component of the intervention intended to prevent/reduce the infection-related outcome of interest; e.g., mask wearing by staff when in contact with residents) are often clearly distinct from the actual intervention being implemented (the measures intended to implement/increase the effective component e.g., an educational intervention promoting mask use by emphasizing the benefits). If an intervention fails to affect the effective component of interest (e.g., an educational intervention does not increase mask use), this would provide insights into the effectiveness of the particular educational intervention, but does not provide information on the effectiveness of mask wearing against e.g., SARS-CoV-2 infections. By contrast, if an intervention significantly increases mask use in the target population, but no difference between intervention and control group regarding the outcomes can be observed, this indicates that the effective component (mask wearing) may not be effective in protecting against the outcome of interest. The effective component could be address through a multiplicity of different implemented interventions (e.g., both a national legislation as well as educational interventions both can aim to reduce the outcome of outbreaks through the effective component of proper mask use among LTCF staff). However, if an effective component itself is not effective against the outcome of interest, any interventions aimed at the effective component will neither.
In this review, the effect of interest was the effect of adherence to the effective component of an intervention, rather than the implemented intervention itself.

## Measures of intervention effect

If a study reported both unadjusted and adjusted intervention effects, we used the adjusted effects and provide a list of the covariates adjust for in the analysis. If a study reported multiple adjusted estimates of an intervention effect, we use the one that we judged to minimize the risk of bias due to confounding (4).

When studies reported outcomes for different populations of interest (e.g., rates of infection among residents and among staff), we preferably report the outcome for the combination of the population groups of interest. If this is not provided in the study, we calculated the effect measure of interest from the data provided. If this was not possible, we prioritize the effect estimate among residents within the evidence synthesis.

Some studies reported on bundles of interventions across multiple intervention domains and categories, without the possibility of estimating the effect of a single effective intervention component. In such cases, we provided effect estimates for a multi-component intervention based on the intervention domains the particular measures fell under (e.g., “combination of different entry regulation measures”).

In our protocol we stated intervention effects may be reported using different effect measures in similar studies reporting on the same intervention and outcome. In these cases, we selected (or calculated, if data allowed) the effect measure, which was used (or can be calculated) in all or most other studies in the same intervention category. If multiple effect measures met this condition, we chose the one with the lowest risk of bias.

**Deviation from the protocol**: If studies reported measurements with multiple time points for the same outcome, we stated in our protocol that we would select the estimate with the longest follow-up period from the intervention. This is of particular relevance in one study included study, where the later time point fell into a period of very low infection rates in the context of a SARS-CoV-2 pandemic. Here, we decided to focus on the first reported time period, in which the risk of infection within the facility was still relatively high and which therefore allowed for a more reliable estimate of the effectiveness of the measures in the context of a pandemic-like event, as was the interest within this study.

## Data synthesis and analysis

For the data synthesis, we planned to conduct a meta-analysis to pool the effects of the intervention in the same intervention domain and category, if at least three applicable studies are available and the data allows (i.e., the effect measure was judged to be sufficiently similar). Due to the heterogeneity of the pathogens under study, we planned to conduct separate analysis regarding the infection-related outcomes for the different pathogens of interest, however pooling all findings for the outcome of adverse- and other unintended consequences. However, none of the intervention domains met this criterion.

As a meta-analysis was not possible or appropriate (e.g., the intervention effects being incompletely reported, or the studies reporting different effect measures) (13), we conducted a narrative synthesis through on vote counting based on the direction of effect, in accordance with the Cochrane Handbook and the SWiM-guidance (13, 14). This would include direction-of-effect tables including potential moderating variables, as well as visualizing the results through direction of effect plots or harvest plots (15, 16).

While we counted the direction of effect, independent of the significance in the direction of effect, we included the significance level in the effect direction plots and in the effect summary description through referring to significant effects as “clear” and non-significant effects as “unclear” effects favouring either intervention or control. This was done, as although the vote counting relied only on the direction of effects (e.g., three studies favouring the intervention and 2 studies favouring the control) we believe this information provides valuable information for judging the certainty of the evidence (for example, as we included the significance of the effect within the GRADE assesses in the domain of “imprecision”). In doing so, we follow the synthesis approach used in a previous Cochrane review this publication is based on.

As a strength of the evidence synthesis through vote counting of the direction of effects allows for a synthesis of findings across multiple different intervention domains and categories, we conducted a data synthesis across the different infection-related outcomes. In accordance with our logic model, we assume that regarding non-pharmacological interventions, infection-related outcomes are closely related. If an intervention prevents an outbreak in a facility, this will lead to fewer infections (detected and undetected). Fewer infections will lead to fewer severe cases and fewer deaths. A key assumption of our work is that the NPIs will not have effects in opposing directions regarding the outcomes grouped within our composite outcome of effectiveness in preventing infection-related outcomes due to viral respiratory pathogens with pandemic potential. This composite outcome aims to reflect that the intervention may reduce the risk of outbreaks in a cluster of LTCFs, may reduce the outbreak size, and due to an overall number of infections may reduce infection-related hospitalizations or deaths among residents and staff in the LTCFs.

Within this composite outcome, we synthesized the direction of effect for the different infection-related outcome measures. In this synthesis, each study provided only one effect estimate per comparison. In instances where a given publication addressed multiple infection-related endpoints (e.g., the study reporting both on number of infections and number of hospitalizations), those were prioritized in the following order: Number, rate, or proportion of (1) contaminations, (2) outbreaks, (3) infections, (4) hospital admission due to infection, and (5) deaths due to infection. We decided to prioritize the outcomes of contaminations and outbreaks, as we assumed that preventing an outbreak in a facility would be overall preferable in comparison to mitigating the effects of ongoing outbreaks. The decision for the order of infection, hospital admissions, and deaths was derived from the overall number of expected events (i.e., in an outbreak, the number of infections will likely be higher than the number of individuals dying from an infection), therefore increasing the power of the analysis.

## Assessment of reporting/publication biases

We aimed to assess reporting/publication bias, if we identify at least 10 comparable studies within the same intervention domain and category. If appropriate, we planned to use visual inspections of funnel plots and perform tests for funnel plot asymmetry (e.g., Egger’s tests) (17). However, this was not possible within our review, due to the limited number of studies available.

## Assessing heterogeneity

As we did not conduct a meta-analysis, we examined heterogeneity per outcome through visual inspection of the effect direction plots (i.e., assessing the heterogeneity in the direction of effects). If the effect of one or more studies out of every three estimates points in a different direction to the others, we had planned to conduct a hypothesis-generating, subgroup analysis through creating separate harvest or effect direction plots in line with the pre-specified subgroup analysis. However, this criterion was not met, therefore we abstained from conducting a heterogeneity assessment as outlined previously.

## Assessment of certainty of evidence and creating summary of findings (SoF) tables

We used the GRADE approach to assess the certainty of primary outcomes (18) and to provide summary of findings (SoF) tables. One review author collated the evidence in SoF tables (18) and developed a preliminary assessment of the certainty of evidence. Afterwards, the result were be checked by and discussed within the research team where a final decision was made (19).

In GRADE, evidence from RCTs enters the rating as high certainty, as does evidence from NRSI whose risk of bias has been assessed using ROBINS-I (20). Therefore, we provided one joint GRADE rating across the three groups of studies to be included in this review. From the starting point of “high”, the rating can be downgraded across five domains (study limitations, inconsistency, indirectness, imprecision (here: referring to whether the confidence interval allowed for a null effect and/or and effect in the opposite direction), and publication bias) and upgraded via three domains (plausible confounding, large effect sizes, and a dose-response relationship).

For the assessment of whether a body of evidence should be downgraded for study limitations, we employed the following overall study limitation classification for the body of evidence:

- **Low RoB**: most information for the outcome is generated from studies at low RoB.
- **Moderate RoB**: most information is from studies at low RoB or studies with some concerns / moderate RoB.
- **High RoB**: the proportion of information from studies at high RoB is sufficient to affect the interpretation of results.

# Appendix V: Extended description of risk of bias of included studies

Across all comparisons in the studies, our focus lay on the direction of effect. To be precise, we assessed the risk that bias, rather than the intervention of interest, was the cause of the reported direction of effect (e.g., observed beneficial effect vs. a “true” null or an adverse effect).

## Risk of bias of included randomized controlled trials

We assessed the risk of bias of cRCTs with ROB-2. The results are presented as traffic light plot (Figure 1), generated with robvis (21). Of the 3 RCTs (22–24) assessed with the RoB2-tool, two cRCTs were classified as having a high risk of bias (22, 23). One study had substantial imbalances in baseline characteristics between the intervention and control groups; suggesting a problem with the randomization process (22). The same study was designed as an observer-blinded and observed-blinded cRCT, but some ward managers refused to keep observations blinded, so some nurses were informed of the purpose of the observations. Furthermore, the authors could not rule out that some observers could figure out which units received the intervention if they saw some of the intervention material (e.g., posters), causing potential observer bias. Another study was judged with high risk of bias mostly due to inconsistencies in the selection of the reported results (23). The authors did not provide sufficient information regarding the multiple measurements analysed and no pre-specified analysis plan was available.

One study (24) was classified at “some concerns”.


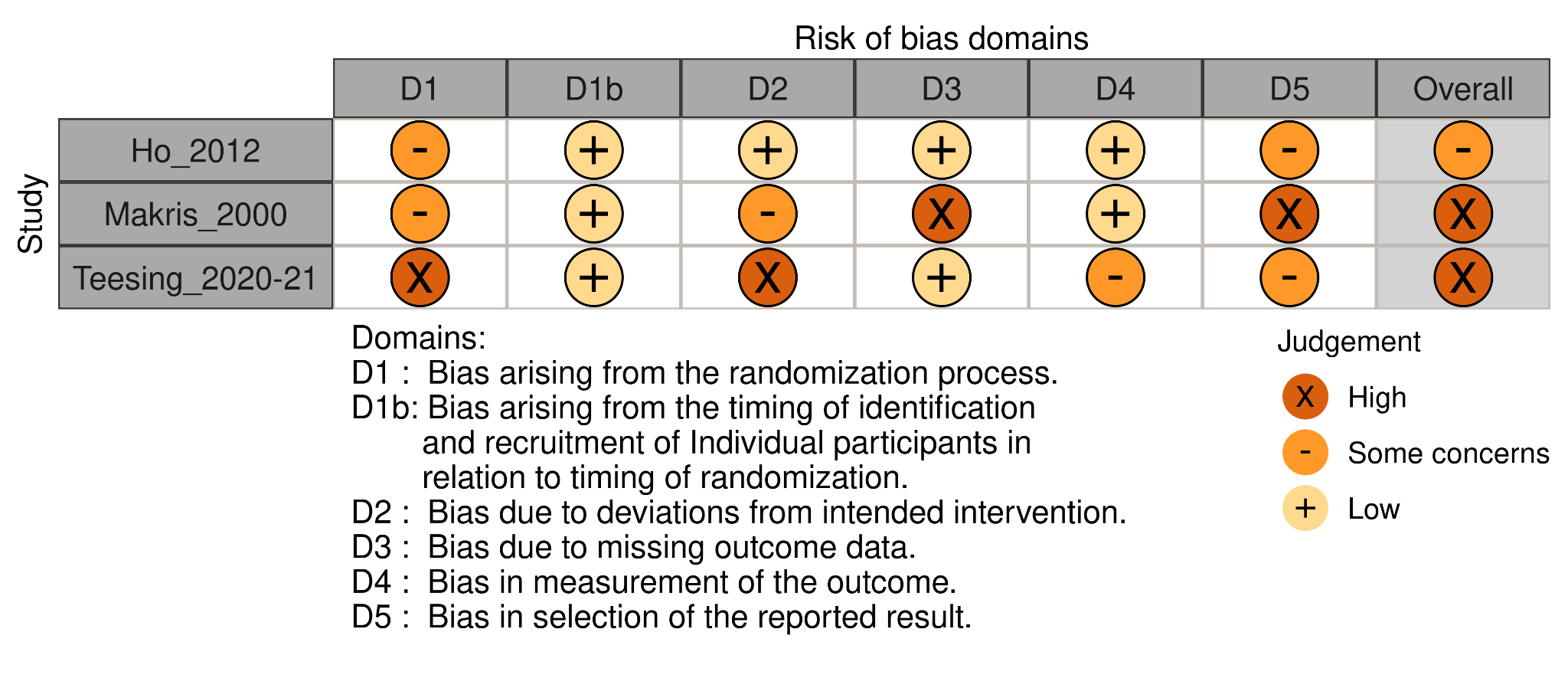


Figure 1 Traffic-light plot for the risk of bias domains assessed with RoB-2 and robvis

## Risk of bias of included observational studies

We assessed the risk of bias of NRSI with ROBINS-I. The results are presented as summary plot (Figure 2) and traffic light plot (Figure 1), both generated with robvis (21). We judged the comparisons in all but two studies (25, 26) to be at serious risk of bias, primarily due to the domain 'bias due to confounding' and the domain 'bias due to deviation from intended intervention'. We judged the domains 'bias in selection of participants into the study', 'bias in classification of intervention', 'bias due to missing data', 'bias in measurement of outcomes', and 'bias in selection of the reported results' at a mix of low, moderate, serious, and unclear risk of bias.


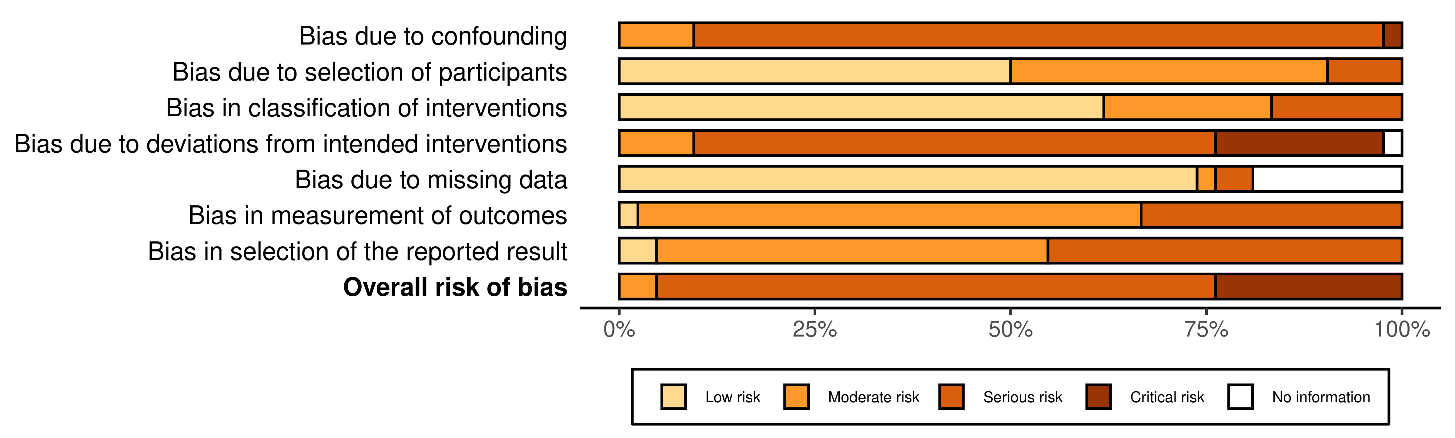


Figure 2 Summary plot for the risk of bias domains assessed with ROBINS-I robvis

We judged most observational studies to have not appropriately measured or controlled for at least one known important confounder. Mostly, as it could not be ruled out that observed associations between the intervention and outcomes of interest could be due to underlying characteristics of the facilities and/or the population within them, which were both associated with the intervention and outcomes. For example, in these cases we mostly could not rule out the possibility that LTCFs of higher “quality” (e.g., better managed, more financial resources) had the intervention in place at the time of assessment due to a higher capacity and/or motivation to introduce protective measures, while at the same time other factors associated with the “quality” of the LTCF, rather than the measure itself, were the cause of the outcome of interest. A more in-depth description and or analysis of LTCFs which reported to have implemented the measure and those without, in order to show their similarity in relevant characteristics, would have strengthened our confidence in their findings.

Our focus lay on implementation and adherence to the effective component of the intervention (e.g., increased hand hygiene), rather than the assigned intervention (e.g., educational intervention to increase hand hygiene). This was assessed in the domain “bias due to deviation from intended intervention”. Due to this domain, two studies were classified as having a critical risk of bias and were excluded from the synthesis (27, 28): Both studies assessed the effect of providing opportunities for voluntary self-testing of staff and visitors. In both studies, the adherence to the testing was very low. Thus, while the study indicates that within the specific contexts, providing opportunities for voluntary self-testing does not increase testing rates, the study cannot contribute any meaningful evidence on the effect of routine testing to prevent/mitigate outbreaks in LTCFs. Meaning, that we judged that a bias due to low adherence to the intended intervention is likely to have strongly contributed to the direction of effect (null effect / effect favouring the control), and therefore the risk of bias to be critically high. However, both studies were well conducted well and we are confident that their finding of no positive effect of providing voluntary testing on infection-related outcomes in these specific settings is valid. The rating of critical risk of bias results from the effect of interest, which is the implementing and adhering to the effective component of routine testing of staff.


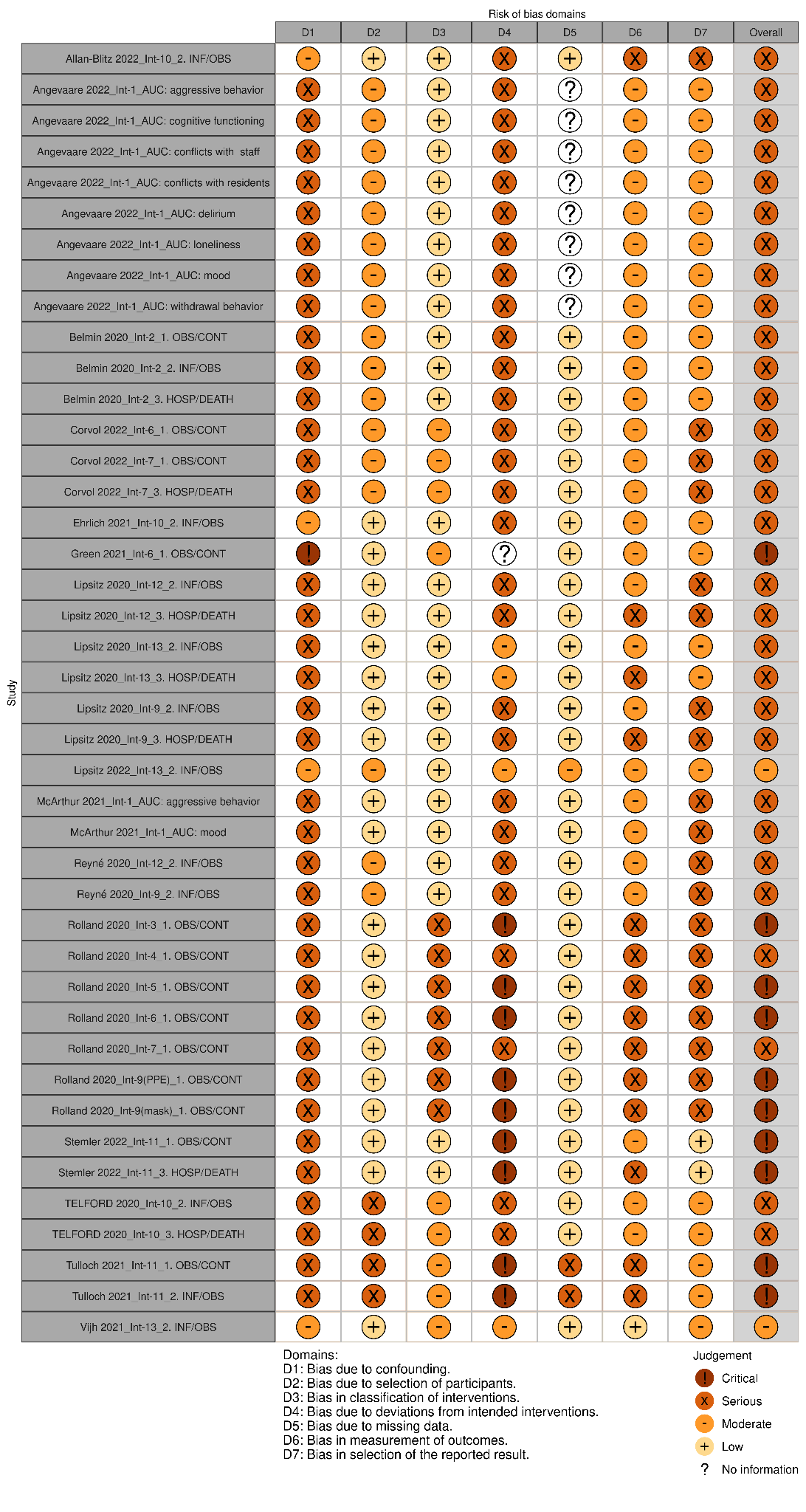


Figure 3 Traffic-light plot for the risk of bias domains assessed with ROBINS-I robvis

Abbreviations used in Figure 3

(Int1) Lockdown measures, including ERM and CRM (Int2) Self-confinement of staff in LTCFs

(Int3) Self-confinement of staff in LTCFs (Int4) Compartmentalization of staff

(Int5) Compartmentalization of residents (Int6) Restrictions in the use of shared spaces

(Int7) Serving meals in room (Int8) Hand hygiene

(Int9) Mask & PPE use (Int10) Routine testing of residents and/or staff

(Int11) Routine testing of staff and visitors (Int12) Cohorting of COVID-19 cases

(Int13) Combination of multi-component measure

In the section on study designs identified in this review, we mentioned studies which employed a cohort-like design, where they (retrospectively) assessed the status regarding one or several interventions of interest, characteristics of the LTCF, and the outcome status over a period of time (29–32). These studies explicitly stated that the measures were implemented prior to the outcome assessment, as we excluded studies where it was not clear, whether the intervention had been introduced prior to the outcome. These types of studies then used different forms of regression-analysis (e.g., multivariate logistic regression) to estimate the association between intervention status and outcome status. Most of comparisons in these studies were judged to be at serious or critical risk of bias within the domain “bias due to deviation from intended intervention”, as they did not adequately account for effects of other infection and control measures implemented across the facilities, and/or due to multicollinearity in the measures implemented in the assessment. For example, one study assessed the association of both, the measure “residents confined to rooms” and the measure “in-room meal service for over 1 month” (29). Based on the data presented, we judged it as likely that most interventions which implemented the measure “residents confined to rooms” also implemented the measure “in-room meal service for over 1 month”. This limits our ability to assess the effect of one intervention independent of the effect of the other. Larger sample sizes, combining of overlapping measures, and/or interaction terms could have helped to reduce the risk of bias in this domain.

# Appendix VI: Effectiveness of NPIs as strategies to protect LTCF residents and staff from infection-related outcomes of viral respiratory pathogens with pandemic potential

## Entry regulation measures

We included one observational study (33), that provided evidence on entry regulation measures, which are depicted in the eﬀect direction plots in **Error! Reference source not found.**.

Table 1 Direction of effect plots for intervention domain entry regulation measures (ERM)

| **Study ID** | **I. Outbreaks /  Contaminations** | **II. Number of infect-ion / outbreak size** | **III. Number of hospitalizations / deaths** | **Impact statement** |
| --- | --- | --- | --- | --- |
|  | *Likelihood of out- breaks or contaminations in LTCFs** | *Number of infections or size of outbreaks* | *Number of severe infections, hospitalizations, or deaths* |  |
| **Self-confinement of staff** | | | | |
| Belmin 2020 | 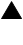 | 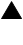 | 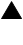 | *One study showed clear beneficial effects regarding the outcomes: (1) risk of outbreaks in facilities, (2) the number of infections, and (3) the number of deaths.* |

The cell colours represent the risk of bias judgment assessed with ROBINS-I: green = low, yellow = moderate, light orange = serious, red = critical. Explanation of the symbols:
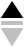
 non-significant effect favouring the intervention,
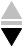
 non-significant effect favouring the control,
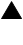
 significant effect favouring the intervention,
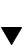
 significant effect favouring the control,
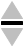
 null effect. Effect estimate in the blue box was used for the estimation of the composite outcome effectiveness in preventing infection-related outcomes.

### Self-confinement of staﬀ with residents

One study from France conducted in the early phase of the SARS-CoV-2 pandemic assessed the eﬀect of self-confinement of staﬀ members with residents as a measure to prevent the introduction of the virus through staﬀ (33). The study showed clear beneficial effects regarding the outcomes risk of outbreaks in facilities, the number of infections, and the number of deaths. Here, 1 out of 17 LTCFs with self-confinement (5.8%) had at least 1 case of COVID-19 among residents, compared with 4599 out of 9513 (48.3%) LTCFs in the national sample which served as a control (OR=0.07, 95% CI: 0.01 to 0.50). The outbreak in the LTCF in the intervention group had occurred prior to the implementation of the measure.

The evidence from this one study suggests that the measure may be effective as a strategy to protect LTCFs in the context of a pandemic-like event. Our confidence in these findings is, however, limited (low certainty evidence). Primarily due to concerns about the risk of bias.

## Contact-regulating measures

We included three observational studies (32, 29, 30) that contributed evidence on contact-regulating measures. Those are depicted in the eﬀect direction plots in **Error! Reference source not found.**.

Table 2 Direction of effect plots for intervention domain contact regulation measures (CRM)

| **Study ID** | **I. Outbreaks /  Contaminations** | **II. Number of infect-ion / outbreak size** | **III. Number of hospitalizations / deaths** | **Impact statement** |
| --- | --- | --- | --- | --- |
|  | *Likelihood of out- breaks or contaminations in LTCFs** | *Number of infections or size of outbreaks* | *Number of severe infections, hospitalizations, or deaths* |  |
| **CRM: Cessation of group activities** | | | | |
| Rolland 2020 | 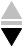 |  |  | *Study excluded from evidence synthesis due to critical risk of bias. No study provided evidence of the beneficial or harmful effect of cessation of group activities as CRM. Thus no GRADE assessment was performed.* |
| **CRM: Compartmentalization of staff members** | | | | |
| Rolland 2020 | 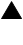 |  |  | *One study showed clear beneficial effects regarding the outcome: (1) risk of outbreaks in facilities.* |
| **CRM: Compartmentalization of residents** | | | | |
| Rolland 2020 | 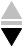 |  |  | *Study excluded from evidence synthesis due to critical risk of bias.*  *No study provided evidence of the beneficial or harmful effect of compartmentalization of staff members as CRM. Thus, no evidence synthesis was performed.* |
| **CRM: Restrictions in the use of shared spaces** | | | | |
| Corvol 2022 | 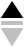 |  |  | *Studies by Green and Rolland 2020 excluded from evidence synthesis due to critical risk of bias.*  *One study showed unclear beneficial effects regarding the outcome: (1) risk of outbreaks in facilities. Two studies were excluded from the evidence synthesis due to critical risk of bias.* |
| Green  2021 | 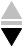 |  |  |  |
| Rolland 2020 | 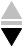 |  |  |  |
| **CRM: Serving meals in room** | | | | |
| Corvol 2022 | 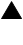 |  | 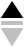 | *One study showed clear beneficial effects regarding the outcome (1) risk of outbreaks in facilities and unclear beneficial effects regarding the outcome (3) number of deaths. Another study showed unclear beneficial effects regarding the outcome (1) risk of outbreaks* |
| Rolland 2020 | 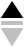 |  |  |  |

The cell colours represent the risk of bias judgment assessed with ROBINS-I: green = low, yellow = moderate, light orange = serious, red = critical (not included in evidence synthesis). Explanation of the symbols:
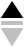
 non-significant effect favouring the intervention,
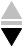
 non-significant effect favouring the control,
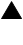
 significant effect favouring the intervention,
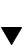
 significant effect favouring the control,
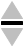
 null effect. Effect estimates in the blue box were used for the estimation of the composite outcome effectiveness in preventing infection-related outcomes.

### Cessation of group activities

One Study was excluded from evidence synthesis due to critical risk of bias. Therefore, no study included in the evidence-synthesis provided evidence on the beneficial or harmful effect of cessation of group activities as a measure to protect LTCFs in the context of a pandemic-like event.

### Compartmentalization of staff members

One study, a cohort study conducted in France in the early phase of the pandemic, contributed evidence on the eﬀect of compartmentalization of staff members as a measure to reduce the likelihood of outbreaks or contaminations in LTCFs (32). Of the 94 LTCFs without a known COVID-19 case, 65 (69%) reported to have implemented staff compartmentalization within zones. In the 30 LTCFs with at least one known COVID-19 case, 9 (30 %) reported to have implemented the measure. In the logistic regression model, accounting for the ownership status of the LTCF, a limited set of LTCF characteristics, and other measures, the aOR was 0.17 (95% CI: 0.04 - 0.07, p-value: 0.01) and thereby favouring the intervention.

The evidence from this study suggests that the measure may be effective as a strategy to protect LTCFs in the context of a pandemic-like event, although our confidence in these findings is limited due to serious risk of bias in the study (low certainty of evidence).

### Compartmentalization of residents

One Study was excluded from evidence synthesis due to critical risk of bias. Therefore, no study included in the evidence-synthesis provided evidence on the beneficial or harmful effect of compartmentalization of residents as a measure to protect LTCFs in the context of a pandemic-like event.

### Restrictions in the use of shared spaces

One study showed unclear beneficial effects of restrictions in the use of shared spaces as a measure to reduce the risk of outbreaks in LTCFs (29). Two additional studies were excluded from the evidence synthesis due to critical risk of bias (32, 30). In the included study, approx. 9 out of 151 LTCFs (6%) with the intervention reported to have at least one infection among residents vs. 15 out of 66 LTCFs (22%) without the intervention. The odds of having at least 1 case among residents was lower among LTCFs who responded affirming to have implemented the measure (aOR: 0.36; 95% CI: 0.08-1.54; p-value: 0.18) in comparison to the LTCFs who did not.

The measure may be effective as a strategy to protect LTCFs in the context of a pandemic-like event, but our confidence in the findings is very limited due to serious risk of bias in the study and concerns due to the imprecision of the effect (very low certainty of evidence).

### Serving meals in rooms

One study showed clear beneficial effects of serving meals in rooms as a measure to reduce the risk of outbreaks in LTCFs (29). Approximately 9 out of 185 LTCFs (5%) with the intervention reported to have at least one infection among residents vs. 15 out of 46 LTCFs (33%) without the intervention. In the multivariate analysis, the odds of reporting at least 1 case among residents was lower among LTCFs who responded affirming to have implemented the measure: in-room meal service for over 1 month: aOR: 0.10 (95% CI: 0.02-0.35; p-value: <0.001). The same study also showed unclear beneficial effects regarding the outcome number of deaths.

Another study showed unclear beneficial effects regarding the outcome risk of outbreaks (32). Here, of the 94 LTCFs without a known COVID-19 case, 41 (43%) reported to offer the meals in the bedroom. In the 30 facilities with at least one known COVID-19 case, 5 (17%) reported to offer the meals in the bedroom. In the logistic regression model, accounting for the ownership status of the LTCF, a limited set of LTCF characteristics, and other measures, the aOR for "organizing of the meals" was: 0.63 (95% CI: 0.34-1.15, p-value: 0.13; thereby favouring the intervention).

As two out of the two studies providing evidence to the composite outcome, the measure may be effective as a strategy to protect LTCFs in the context of a pandemic-like event. However, our confidence in the findings is very limited due to risk of bias in the included studies and the one of the two studies not reaching the level of statistical significance (very low certainty of evidence).

## Transmission-reduction measures

We included six studies (24, 22, 32, 34, 31, 23) that contributed evidence on transmission-reduction measures: three cRCTs assessing the effect of measures aimed at improving hand hygiene against infection-related outcomes due influenza and respiratory tract infections, as well as 3 NRSI assessing the effect of mask and PPE use.

Table 3 Direction of effect plots for intervention domain transmission-reduction measures (TRM)

| **Study ID** | **I. Outbreaks /  Contaminations** | **II. Number of infect-ion / outbreak size** | **III. Number of hospitalizations / deaths** | **Impact statement** |
| --- | --- | --- | --- | --- |
|  | *Likelihood of out- breaks or contaminations in LTCFs** | *Number of infections or size of outbreaks* | *Number of severe infections, hospitalizations, or deaths* |  |
| **TRM: Hand hygiene** | | | | |
| Ho 2012 | 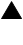 |  |  | *One study showed clear beneficial effects regarding the outcome (1) risk of outbreaks in facilities. Two studies showed clear beneficial effects regarding the outcome (2) number of infections.* |
| Teesing 2021 |  | 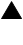 |  |  |
| Makris 2000 |  | 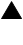 |  |  |
| The cell colours represent the risk of bias judgment assessed with ROB2: green = low, yellow = some concerns, orange = serious, red = critical. | | | | |
| **TRM: Mask & PPE use** | | | | |
| Rolland 2020 | 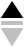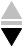 |  |  | *One study showed clear beneficial effects regarding the outcome (2) number of infections and unclear beneficial effects regarding (3) number of deaths. Another study showed unclear beneficial effects regarding (2) number of infections. One study was excluded due to critical risk of bias.* |
| Lipsitz 2020 |  | 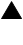 | 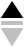 |  |
| Reyné 2020 |  | 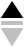 |  |  |

The cell colours represent the risk of bias judgment assessed with ROBINS-I: green = low, yellow = moderate, orange = serious, red = critical.
Explanation of the symbols:
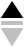
 non-significant effect favouring the intervention,
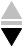
 non-significant effect favouring the control,

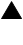
 significant effect favouring the intervention,
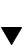
 significant effect favouring the control,
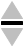
 null effect. Effect estimates in the blue box were used for the estimation of the composite outcome effectiveness in preventing infection-related outcomes.

### Hand hygiene measures

One study showed clear beneficial effects of hand hygiene as a measure to reduce the risk of outbreaks in LTCFs (24). Two studies showed clear beneficial effects regarding the outcome number of infections (22, 23).

The first study was a cluster RCT conducted in the USA. Here, LTCFs in the intervention group were provided with a combined approach consisting of an infection control educational program focusing on hand hygiene along with the regular use of disinfectants and cleaning solutions for a 12-month period. The number of upper respiratory tract infections showed a significant median decrease of 58% during the intervention period - the control sites showed a median decrease of 33% (24).

In the second cluster RCT, 66 LTCFs with 1862 beds in the Netherlands were randomly assigned to a multimodal intervention included a combination of activities for changing hygiene policy and the individual behaviour of nurses, including providing hand-hygiene materials. In the study, compliance with hand hygiene guidance was associated with significantly fewer influenza-like illnesses (IRR=0.51, 95% CI: 0.31 to 0.82) in the intervention arm when compared to the control arm (35, 23, 36, 37).

In the third cluster RCT conducted in Hong Kong, 18 LTCFs with 2407 residents were randomly assigned to a multi-modal approach to increase hand hygiene, including educational components as well as providing alcohol-based hand rub. The study found a significant reduction in respiratory outbreaks (IRR=0.12; 95% CI: 0.01 to 0.93) in the intervention group (23).

Three out of three studies showed beneficial effects favouring the intervention. The evidence from this study suggests that the measure can be effective as a strategy to protect LTCFs in the context of a pandemic-like event (moderate certainty of evidence).

### Mask and PPE usage

One study assessed the effect of different infection-control measures implemented in LTCFs in Massachusetts, USA. The study showed clear beneficial effects of mask and PPE use as a measure to reduce the number of infections in LTCFs and unclear beneficial effects regarding the outcome number of deaths (34). In the study, PPE use was associated with a reduction of 23 % in the weekly infection rate (95% CI: -0.45 to -0.01) and an increased odd of having an infection rate of zero (OR = 2.16; 95% CI: 1.42 to 3.30). A second study assessed the effects of delayed implementation of generalized mask-wearing in outbreaks of SARS-CoV-2. There was a non-significant increase in the risk of infection (defined through the proportion of contaminated residents at the conclusion of the outbreak) per additional day in delay of generalized mask wearing (aOR: 1.03; 95% CI: 0.99 to 1.08). The study provided multiple calculations using different covariables in the regression model. While the effect of mask wearing was statistically significant in a number of models, the model we selected due to judging it to have the lowest risk of bias, the effect of mask wearing did not reach the level of statistical significance (31).
A third study was excluded from the evidence synthesis due to critical risk of bias. In summary, two out of two studies showed an effect in favour of the measure. This indicates that the measure may be effective as a strategy to protect LTCFs in the context of a pandemic-like event. However, our confidence in the finding is very limited, primarily due to major concerns regarding the risk of bias of the included studies (very low certainty of evidence).

## Screening and surveillance measures

We included three studies (38–40), that provided evidence on screening and surveillance measures, which are depicted in the eﬀect direction plots in **Error! Reference source not found.**.

Table 4 Direction of effect plots for intervention domain surveillance screening measures (SSM)

| **Study ID** | **I. Outbreaks /  Contaminations** | **II. Number of infect-ion / outbreak size** | **III. Number of hospitalizations / deaths** | **Impact statement** |
| --- | --- | --- | --- | --- |
|  | *Likelihood of out- breaks or contaminations in LTCFs** | *Number of infections or size of outbreaks* | *Number of severe infections, hospitalizations, or deaths* |  |
| **SSM: Routine testing of residents and/or staff** | | | | |
| Allan-Blitz 2022 |  | 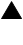 |  | *Three study showed clear beneficial effects regarding the outcome (2) number of infections and one study also showed clear beneficial effects regarding the outcome (3) number of deaths* |
| Ehrlich 2021 |  | 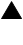 |  |  |
| Telford 2020 |  | 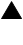 | 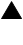 |  |

| **SSM: Routine testing of staff and visitors** | | | | |
| --- | --- | --- | --- | --- |
| Stemler 2022 | 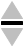 |  | 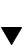 | *Two studies were excluded due to critical risk of bias. No study provided evidence of the beneficial or harmful effect of routine testing of staff and visitors as SSM. Thus, no evidence synthesis was performed.* |
| Tulloch 2021 | 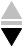 | 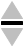 |  |  |

The cell colours represent the risk of bias judgment assessed with ROBINS-I: green = low, yellow = moderate, orange = serious, red = critical. Explanation of the symbols:
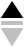
 non-significant effect favouring the intervention,
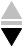
 non-significant effect favouring the control,
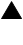
 significant effect favouring the intervention,
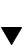
 significant effect favouring the control,
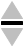
 null effect. Effect estimates in the blue box were used for the estimation of the composite outcome effectiveness in preventing infection-related outcomes.

**Routine testing of residents and/or staff:** Three NRSI assessing the effect of routine, symptom-independent testing of residents and staff using PCR-based tests in the first year of the SARS-CoV-2 pandemic to prevent outbreaks and mitigate their consequences in LTCFs.

One study assessed the effect of generalized testing in an LTCF as a response to the detection of a case following symptom-based screening (the control group) with LTCFs where generalized, symptom-independent screening was conducted. In the control group of 15 LTCFs, 42.4% of residents (723/1705) and 11.8% of staff members (230/1944) tested positive for SARS-CoV-2 through mass screening followed by 4 weeks of symptom-based screening. Out of the 13 LTCFs in the intervention group 8 LTCFs reported at least 1 case among residents or staff. Within the contaminated LTCFs in the IG, 1.8% of residents (17/919) and 2.2% of staff members (15/697) were identified as infected with SARS-CoV-2 through mass screening and 4 weeks of symptom-based screening (own calculation) (40). The study showed clear beneficial effects regarding the number of infections as well as clear beneficial effects regarding the outcome number of deaths (40).

A second study used longitudinal data to assess the effect of introducing state-wide mandate for semi-monthly testing of all residents and staff of LTCFs using RT-PCR-based tests in LTCFs in Florida in the first and second wave of the SARS-CoV-2 pandemic in the US. Following the introduction of the mandate, a 1% increase in testing rates (defined as number of tests per occupied bed) resulted in a 0.08% reduction (95%CI: –0.14 to –0.02) in weekly cases 3 weeks after testing began (38).

All three studies showed clear beneficial effects of routine testing of residents and/or staff as a measure to reduce the number of infections in LTCFs (38–40).

A third study conducted in the early phase of the SARS-CoV-2 pandemic assessed the effects of regular testing of all residents and staff of LTCFs using RT-PCR-based tests (mean duration between test round 1 and test round 2 was 30 days). After adjusting for community incidence and the change in screening practices, the implementation of serial testing was associated with a significant decrease in nursing home incidence rates of 77% (95% CI 71%–83%) in the first 15 days after the first generalized round of testing, of 41% (95% CI 12%–60%) from days 31–60, and 80% (95% CI 64%–89%) reduction from days 61–90, compared with the period before the generalized screening.

In summary, three out of three studies which contributed evidence to the composite outcome showed clear effects favouring the intervention. This suggests that the measure may be effective as a strategy to protect LTCFs in the context of a pandemic-like event. However, our confidence in the findings is limited primarily due the risk of bias in the included studies (low certainty of evidence).

**Routine testing of staff and visitors:** Two studies were excluded from the analysis due to critical risk of bias. Therefore, no study included in the evidence-synthesis provided evidence on the beneficial or harmful effect of routine testing of staff and visitors as a strategy to protect LTCFs in the context of a pandemic-like event.

## Outbreak control measures

We included two studies (34, 31), that provided evidence on outbreak control measures, which are depicted in the eﬀect direction plots in **Error! Reference source not found.**.

Table 5 Direction of effect plots for intervention domain outbreak control measures (OCM)

| **Study ID** | **I. Outbreaks /  Contaminations** | **II. Number of infect-ion / outbreak size** | **III. Number of hospitalizations / deaths** | **Impact statement** |
| --- | --- | --- | --- | --- |
|  | *Likelihood of out- breaks or contaminations in LTCFs** | *Number of infections or size of outbreaks* | *Number of severe infections, hospitalizations, or deaths* |  |
| **OCM: Cohorting of cases** | | | | |
| Lipsitz 2020 |  | 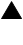 | 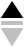 | *One study showed clear beneficial effects regarding the outcome (2) number of infections and unclear beneficial effects regarding (3) number of deaths. Another study showed unclear beneficial effects regarding (2) number of infections.* |
| Reyné 2020 |  | 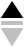 |  |  |

The cell colours represent the risk of bias judgment assessed with ROBINS-I: green = low, yellow = moderate, orange = serious, red = critical. Explanation of the symbols:
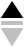
 non-significant effect favouring the intervention,
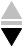
 non-significant effect favouring the control,
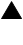
 significant effect favouring the intervention,
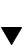
 significant effect favouring the control,
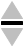
 null effect. Effect estimates in the blue box were used for the estimation of the composite outcome effectiveness in preventing infection-related outcomes.

***Separating infected and non-infected residents (Cohorting):*** One study assessed measures which were associated with a lower overall number of infections in a ward in the context of ongoing outbreaks due to SARS-CoV-2 in French LTCFs. In the context of weekly generalized testing and additional infection and control practices and measures in place, the presence of a COVID-19 unit was associated with non-significant reduction proportion of infected residents at the conclusion of the outbreak (aOR: 0.46; 95% CI: 0.14 to 1.87) (31).
A second study assessed whether cohorting, in the sense of residents who were confirmed by testing to be infected with SARS-CoV-2 were separated from residents who were not infected or had unknown status in 360 LTCFs in the first wave of SARS-CoV-2 in the USA. According to the study, cohorting was associated with a reduction of 50% in the weekly infection rate (95% CI: -0.84 to -0.16) and increased odds of a weekly infection rate of zero (OR = 3.00; 95% CI: 1.34 to 6.71) (34).

As two out of two studies showed an effect in favour of the intervention, the measure may be effective as a strategy to protect LTCFs in the context of a pandemic-like event. However, our confidence in these findings is limited due to the risk of bias of the included studies and the non-significant effect in one of these two studies (very low certainty of evidence).

## Combination of multiple measures across multiple intervention domains

We included three studies (34, 25, 26), that provided evidence on the combination of multiple measures across multiple intervention domains, which are depicted in the eﬀect direction plots in **Error! Reference source not found.**.

Table 6 Direction of effect plots for combination of multiple measures across multiple intervention domains (multi-component measures - MCM)

| **Study ID** | **I. OBS/CONT** | **II. INF/OBS** | **III. HOSP/DEATH** | **Impact statement** |
| --- | --- | --- | --- | --- |
|  | *Likelihood of out- breaks or contaminations in LTCFs** | *Number of infections or size of outbreaks* | *Number of severe infections, hospitalizations, or deaths* |  |
| **MCM: Combination of multi-component measures** | | | | |
| Lipsitz 2020 |  | 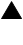 | 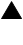 | *Three studies showed clear beneficial effects regarding the outcome (2) number of infections. One study also showed clear beneficial effects regarding the outcome (3) number of deaths.* |
| Lipsitz 2022 |  | 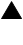 |  |  |
| Vijh 2021 |  | 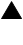 |  |  |

The cell colours represent the risk of bias judgment assessed with ROBINS-I: green = low, yellow = moderate, orange = serious, red = critical. Explanation of the symbols:
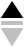
 non-significant effect favouring the intervention,
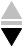
 non-significant effect favouring the control,
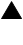
 significant effect favouring the intervention,
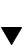
 significant effect favouring the control,
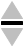
 null effect. Effect estimates in the blue box were used for the estimation of the composite outcome effectiveness in preventing infection-related outcomes.

***multi-component measures including ERM, CRM, TRM and OCM:*** Three NRSI showed clear beneficial effects of combining multi-component interventions as a measure to reduce the number of infections (34, 25, 26). One of these studies also showed clear beneficial effects regarding the reduction of the number of deaths (34). Two of these studies were the only NRSI judged to have a moderate, rather than a serious risk of bias among all NRSI assessed in this review. The measure can be effective as a strategy to protect LTCFs in the context of a pandemic-like event (moderate certainty of evidence).

# Appendix VII: Summary of Findings tables

Summary of findings 1. Entry regulation measures (ERM)

| **Number and type of studies** | **Overall number of LTCFs, residents & staff** | **Impact statement** | **Certainty of evidence** | **Summary of findings** |
| --- | --- | --- | --- | --- |
| **Intervention category 1. Self-confinement of staff** | | | | |
| One NRSI (CCS) | 1 Study; 17 LTCFs with 1250 residents | One study showed clear beneficial effects regarding the outcomes:  (1) risk of outbreaks in facilities, (2) the number of infections, and  (3) the number of deaths. | Low^[[1]](#footnote-1)^  ⨁⨁◯◯ | The measure may be effective as a strategy to protect LTCFs in the context of a pandemic-like event. |

Summary of findings 2. Contact-regulation measures (CRM) and transmission-reduction measures (TRM)

| **Number and type of studies** | **Overall number of LTCFs, residents & staff** | **Impact statement** | **Certainty of evidence** | **Summary of findings** |
| --- | --- | --- | --- | --- |
| **Intervention category 1. CRM: Cessation of group activities** | | | | |
| One NRSI (CCS) | 1 Study 124 LTCFs | No study provided evidence of the beneficial or harmful effect of cessation of group activities as CRM^[[2]](#footnote-2)^. Thus no GRADE assessment was performed. |  |  |
| **Intervention category 2. CRM: Compartmentalization of staff members** | | | | |
| One NRSI (CCS) | 1 observational study 124 LTCFs | One study showed clear beneficial effects regarding the outcome:  (1) risk of outbreaks in facilities. | Low^[[3]](#footnote-3)^  ⨁⨁◯◯ | The measure may be effective as a strategy to protect LTCFs in the context of a pandemic-like event. |
| **Intervention category 3. CRM: Compartmentalization of residents** | | | | |
| One NRSI (CCS) | 1 observational study 124 LTCFs | No study provided evidence of the beneficial or harmful effect of compartmentalization of residents as CRM or TRM^2^. Thus no evidence synthesis was performed. |  |  |
| **Intervention category 4. CRM: Restrictions in the use of shared spaces** | | | | |
| Three NRSIs (CCS) | 389 LTCFs with 21737 residents | One study^[[4]](#footnote-4)^ showed unclear beneficial effects regarding the outcome: (1) risk of outbreaks in facilities. Two studies were excluded from the evidence synthesis due to critical risk of bias. | Very low ^[[5]](#footnote-5)^  ⨁◯◯◯ | The measure may be effective as a strategy to protect LTCFs in the context of a pandemic-like event but the evidence is very uncertain. |
| **Intervention category 5. CRM: Serving meals in room** | | | | |
| Two NRSIs (CCS) | 355 LTCFs with 20881 residents | One study^[[6]](#footnote-6)^ showed clear beneficial effects regarding the outcome (1) risk of outbreaks in facilities and unclear beneficial effects regarding the outcome (3) number of deaths. Another study showed unclear beneficial effects regarding the outcome (1) risk of outbreaks. | Very low ^[[7]](#footnote-7)^  ⨁◯◯◯ | The measure may be effective as a strategy to protect LTCFs in the context of a pandemic-like event but the evidence is very uncertain. |
| **Intervention category 6. TRM: Hand hygiene** | | | | |
| Three cRCTs | 92 LTCFs with 5159 residents and 810 staff members | One study showed clear beneficial effects regarding the outcome (1) risk of outbreaks in facilities. Two studies showed a clear beneficial effects regarding the outcome (2) number infections. | Moderate ^[[8]](#footnote-8)^  ⨁⨁⨁◯ | The measure can be effective as a strategy to protect LTCFs in the context of a pandemic-like event |
| **Intervention category 7. TRM: Mask & PPE use** | | | | |
| Three NRSIs | 496 LTCFs with 930 residents and 360 staff members | Two studies^6^ showed clear beneficial effects regarding the outcome (2) number of infections and one study showed an unclear beneficial effects regarding the outcome (3) number of deaths. | Very low ^[[9]](#footnote-9)^  ⨁◯◯◯ | The measure may be effective as a strategy to protect LTCFs in the context of a pandemic-like event but the evidence is very uncertain. |

Summary of findings 3: Surveillance screening measures (SSM)

| **Number and type of studies** | **Overall number of LTCFs, residents & staff** | **Impact statement** | **Certainty of evidence** | **Summary of findings** |
| --- | --- | --- | --- | --- |
| **Intervention category 1. SSM: Routine testing of residents and/or staff** | | | | |
| Three NRSIs | 423 LTCFs with 7458 residents and 2803 staff members | Three study showed clear beneficial effects regarding the outcome  (2) number of infections and one study also showed clear beneficial effects regarding the outcome (3) number of deaths | Low^[[10]](#footnote-10)^  ⨁⨁◯◯ | The measure may be effective as a strategy to protect LTCFs in the context of a pandemic-like event. |
| **Intervention category 2. SSM: Routine testing of staff and visitors** | | | | |
| Two NRSIs | 147 LTCFs with 1105 residents and 1094 staff members | No study^[[11]](#footnote-11)^ provided evidence on the beneficial or harmful effect of routine testing of staff and visitors as SSM. Thus no evidence synthesis was performed. |  |  |

Summary of findings 4: Outbreak control measures (OCM)

| **Number and type of studies** | **Overall number of LTCFs, residents & staff** | **Impact statement** | **Certainty of evidence** | **Summary of findings** |
| --- | --- | --- | --- | --- |
| **Intervention category 1. OCM: Cohorting cases** | | | | |
| Two NRSIs | 372 LTCFs with 930 residents and 360 staff members | One study^[[12]](#footnote-12)^ showed clear beneficial effects regarding the outcome  (2) number of infections and unclear beneficial effects regarding the outcome (3) number of deaths. Another study showed unclear beneficial effects regarding the outcome (2) number of infections. | Very low ^[[13]](#footnote-13)^  ⨁◯◯◯ | The measure may be effective as a strategy to protect LTCFs in the context of a pandemic-like event but the evidence is very uncertain. |

Summary of findings 5: Multi-component measures (MCM)

| **Number and type of studies** | **Overall number of LTCFs and residents** | **Impact statement** | **Certainty of evidence** | **Summary of findings** |
| --- | --- | --- | --- | --- |
| **Intervention category 1. MCM: Combination of multi-component measures** | | | | |
| Three NRSIs | 432 LTCFs with 7722 residents and 1298 staff members | Three studies showed clear beneficial effects regarding the outcome (2) number of infections. One study also showed clear beneficial effects regarding the outcome (3) number of deaths. | Moderate ^[[14]](#footnote-14)^  ⨁⨁⨁◯ | The measure can be effective as a strategy to protect LTCFs in the context of a pandemic-like event. |

Summary of findings 6: Adverse and other unintended consequences (AUC)

| **Number and type of studies** | **Overall number of LTCFs and residents** | **Impact statement** | **Certainty of evidence** | **Summary of findings** |
| --- | --- | --- | --- | --- |
| **Intervention category 1. Lockdown measures, including visiting restrictions (ERM) and measures to reduce contact among residents (CRM)** | | | | |
| Two NRSIs | 65 LTCFs with 1343 residents | Two studies showed unclear and conflicting effects regarding the outcome (4) number of adverse and other unintended consequences in LTCFs. Direction of effect favoured the intervention in 6 outcome measures and favoured the control in 4 outcome measures. | Very low ^[[15]](#footnote-15)^  ⨁◯◯◯ | The evidence is very uncertain and is in line with the measure increasing, decreasing and not affecting adverse mental health consequences among residents. |

# Appendix References

1. Stratil JM, Biallas RL, Burns J, Arnold L, Geffert K, Kunzler AM et al. Non-pharmacological measures implemented in the setting of long-term care facilities to prevent SARS-CoV-2 infections and their consequences: a rapid review. Cochrane Database Syst Rev 2021; 9:CD015085. DOI: 10.1002/14651858.CD015085.pub2.

2. Cochrane Effective Practice and Organisation of Care (EPOC). What study designs can be considered for inclusion in an EPOC review and what should they be called?; 2017 [cited 2022 Dec 8]. Available from: URL: https://epoc.cochrane.org/sites/epoc.cochrane.org/files/public/uploads/Resources-for-authors2017/what_study_designs_should_be_included_in_an_epoc_review.pdf.

3. Reeves BC, Deeks JJ, Higgins JP, Shea B, Tugwell P, Wells GA. Chapter 24: Including non-randomized studies on intervention effects. In: Higgins J, Thomas J, Chandler J, Cumpston M, Li T, Page M et al., editors. Cochrane Handbook for Systematic Reviews of Interventions. 6.3 (updated February 2022); 2022 [cited 2022 Dec 8]. Available from: URL: https://training.cochrane.org/handbook/current/chapter-24.

4. Reeves BC, Wells GA, Waddington H. Quasi-experimental study designs series-paper 5: a checklist for classifying studies evaluating the effects on health interventions-a taxonomy without labels. J Clin Epidemiol 2017; 89:30–42. DOI: 10.1016/j.jclinepi.2017.02.016.

5. ECDC Public Health Emergency Team, Danis K, Fonteneau L, Georges S, Daniau C, Bernard-Stoecklin S et al. High impact of COVID-19 in long-term care facilities, suggestion for monitoring in the EU/EEA, May 2020. Euro Surveill 2020; 25(22). DOI: 10.2807/1560-7917.ES.2020.25.22.2000956.

6. Adalja AA, Watson M, Toner ES, Cicero A, Inglesby TV. Characteristics of Microbes Most Likely to Cause Pandemics and Global Catastrophes. In: Global Catastrophic Biological Risks. Springer, Cham; 2019. p. 1–20 Available from: URL: https://link.springer.com/chapter/10.1007/82_2019_176.

7. Stratil JM, Voss M, Arnold L. WICID framework version 1.0: criteria and considerations to guide evidence-informed decision-making on non-pharmacological interventions targeting COVID-19. BMJ Glob Health 2020; 5(11):e003699. DOI: 10.1136/bmjgh-2020-003699.

8. Jefferson T, Del Mar CB, Dooley L, Ferroni E, La Al-Ansary, Bawazeer GA et al. Physical interventions to interrupt or reduce the spread of respiratory viruses. Cochrane Database of Systematic Reviews 2020; (11). DOI: 10.1002/14651858.CD006207.pub5.

9. Sterne JAC, Savović J, Page MJ, Elbers RG, Blencowe NS, Boutron I et al. RoB 2: a revised tool for assessing risk of bias in randomised trials. BMJ 2019:l4898. DOI: 10.1136/bmj.l4898.

10. Eldridge S, Campbell M, Campbell M, Dahota A, Giraudeau B, Higgins J, Reeves B, Siegfried N. Revised Cochrane risk of bias tool for randomized trials (RoB 2.0): additional considerations for cluster-randomized trials; 2016 [cited 2022 Dec 8]. Available from: URL: https://www.unisa.edu.au/contentassets/72bf75606a2b4abcaf7f17404af374ad/rob2-0_cluster_parallel_guidance.pdf.

11. Sterne JA, Hernán MA, Reeves BC, Savović J, Berkman ND, Viswanathan M et al. ROBINS-I: a tool for assessing risk of bias in non-randomised studies of interventions. BMJ 2016; 355:i4919. DOI: 10.1136/bmj.i4919.

12. Sterne JA, Hernán MA, McAleenan A, Reeves BC, Higgins JP. Chapter 25: Assessing risk of bias in a non-randomized study. In: Higgins J, Thomas J, Chandler J, Cumpston M, Li T, Page M et al., editors. Cochrane Handbook for Systematic Reviews of Interventions. 6.3 (updated February 2022); 2022 Available from: URL: https://training.cochrane.org/handbook/current/chapter-25.

13. McKenzie JB. Chapter 12: Synthesizing and presenting findings using other methods. In: Higgins J, Thomas J, Chandler J, Cumpston M, Li T, Page M et al., editors. Cochrane Handbook for Systematic Reviews of Interventions. version 6.2; 2021.

14. Campbell M, McKenzie JE, Sowden A, Katikireddi SV, Brennan SE, Ellis S et al. Synthesis without meta-analysis (SWiM) in systematic reviews: reporting guideline. BMJ 2020; 368:l6890. DOI: 10.1136/bmj.l6890.

15. Ogilvie D, Fayter D, Petticrew M, Sowden A, Thomas S, Whitehead M et al. The harvest plot: A method for synthesising evidence about the differential effects of interventions. BMC Med Res Methodol 2008; 8(1). DOI: 10.1186/1471-2288-8-8.

16. Thomson HJ, Thomas S. The effect direction plot: visual display of non‐standardised effects across multiple outcome domains. Res. Syn. Meth. 2013; 4(1):95–101. DOI: 10.1002/jrsm.1060.

17. Page M, Higgins J, Sterne J. Chapter 13: Assessing risk of bias due to missing results in a synthesis. In: Higgins J, Thomas J, Chandler J, Cumpston M, Li T, Page M et al., editors. Cochrane Handbook for Systematic Reviews of Interventions. 6.3 (updated February 2022); 2022 Available from: URL: https://training.cochrane.org/handbook/current/chapter-13.

18. Guyatt G, Oxman AD, Akl EA, Kunz R, Vist G, Brozek J et al. GRADE guidelines: 1. Introduction—GRADE evidence profiles and summary of findings tables. J Clin Epidemiol 2011; 64(4):383–94. DOI: 10.1016/j.jclinepi.2010.04.026.

19. Schünemann H, Higgins J, Vist G, Glasziou P, Akl E, Skoetz N et al. Chapter 14: Completing ‘Summary of findings’ tables and grading the certainty of the evidence. In: Higgins J, Thomas J, Chandler J, Cumpston M, Li T, Page M et al., editors. Cochrane Handbook for Systematic Reviews of Interventions. 6.3 (updated February 2022); 2022 [cited 2022 Dec 8]. Available from: URL: https://training.cochrane.org/handbook/current/chapter-14.

20. Schünemann HJ, Cuello C, Akl EA, Mustafa RA, Meerpohl JJ, Thayer K et al. GRADE guidelines: 18. How ROBINS-I and other tools to assess risk of bias in nonrandomized studies should be used to rate the certainty of a body of evidence. J Clin Epidemiol 2019; 111:105–14. DOI: 10.1016/j.jclinepi.2018.01.012.

21. McGuinness LA, Higgins JPT. Risk-of-bias VISualization (robvis): An R package and Shiny web app for visualizing risk-of-bias assessments. Res. Syn. Meth. 2020; n/a(n/a). DOI: 10.1002/jrsm.1411.

22. Makris AT, Morgan L, Gaber DJ, Richter A, Rubino JR. Effect of a comprehensive infection control program on the incidence of infections in long-term care facilities. Am J Infect Control 2000; 28(1):3–7. Available from: URL: https://www.ncbi.nlm.nih.gov/pubmed/10679130. DOI: 10.1016/s0196-6553(00)90004-x.

23. Teesing GR, Richardus JH, Nieboer D, Petrignani M, Erasmus V, Verduijn-Leenman A et al. The effect of a hand hygiene intervention on infections in residents of nursing homes: a cluster randomized controlled trial. Antimicrob Resist Infect Control 2021; 10(1). Available from: URL: https://www.ncbi.nlm.nih.gov/pubmed/34016156. DOI: 10.1186/s13756-021-00946-3.

24. Ho M, Seto W, Wong L, Wong T. Effectiveness of multifaceted hand hygiene interventions in long-term care facilities in Hong Kong: a cluster-randomized controlled trial. Infect Control Hosp Epidemiol 2012; 33(8):761–7. Available from: URL: https://www.ncbi.nlm.nih.gov/pubmed/22759542. DOI: 10.1086/666740.

25. Lipsitz LA, Kosar C, Dufour AB, Travison TG, Mor V. Evaluation of a state-wide effort to improve COVID-19 infection control in Massachusetts nursing homes. Journal of the American Geriatrics Society 2022. DOI: 10.1111/jgs.17984.

26. Vijh R, Prairie J, Otterstatter MC, Hu Y, Hayden AS, Yau B et al. Evaluation of a multisectoral intervention to mitigate the risk of severe acute respiratory coronavirus virus 2 (SARS-CoV-2) transmission in long-term care facilities. Infection Control & Hospital Epidemiology 2021; 42(10):1181–8. DOI: 10.1017/ice.2020.1407.

27. Stemler J, Kramer T, Dimitriou V, Wieland U, Schumacher S, Sprute R et al. Mobile PCR-based surveillance for SARS-CoV-2 to reduce visiting restrictions in nursing homes during the COVID-19 pandemic: a pilot study. Infection 2022; 50(3):607–16. DOI: 10.1007/s15010-021-01716-4.

28. Tulloch JSP, Micocci M, Buckle P, Lawrenson K, Kierkegaard P, McLister A et al. Enhanced lateral flow testing strategies in care homes are associated with poor adherence and were insufficient to prevent COVID-19 outbreaks: results from a mixed methods implementation study. Age and ageing 2021; 50(6):1868–75. DOI: 10.1093/ageing/afab162.

29. Corvol A, Charras K, Prud’homm J, Lemoine F, Ory F, Viel JF et al. Structural and Managerial Risk Factors for COVID-19 Occurrence in French Nursing Homes. International Journal of Health Policy and Management 2022; 11(11):2630–7. DOI: 10.34172/ijhpm.2022.6741.

30. Green R, Tulloch JSP, Tunnah C, Coffey E, Lawrenson K, Fox A et al. COVID-19 testing in outbreak-free care homes: what are the public health benefits? J Hosp Infect 2021; 111:89–95. DOI: 10.1016/j.jhin.2020.12.024.

31. Reyné B, Selinger C, Sofonea MT, Miot S, Pisoni A, Tuaillon E et al. Analysing different exposures identifies that wearing masks and establishing COVID-19 areas reduce secondary-attack risk in aged-care facilities. Int J Epidemiol 2022; 50(6):1788–94. DOI: 10.1093/ije/dyab121.

32. Rolland Y, Lacoste MH, Mauleon A de, Ghisolfi A, Souto Barreto P de, Blain H et al. Guidance for the Prevention of the COVID-19 Epidemic in Long-Term Care Facilities: A Short-Term Prospective Study. Journal of Nutrition, Health and Aging 2020; 24(8):812–6. Available from: URL: https://www.ncbi.nlm.nih.gov/pubmed/33009529. DOI: 10.1007/s12603-020-1440-2.

33. Belmin J, Um-Din N, Donadio C, Magri M, Nghiem QD, Oquendo B et al. Coronavirus Disease That Implemented Staff Confinement with Residents. JAMA Network Open 2020; 3(8).

34. Lipsitz LA, Lujan AM, Dufour A, Abrahams G, Magliozzi H, Herndon L et al. Stemming the Tide of COVID-19 Infections in Massachusetts Nursing Homes. Journal of the American Geriatrics Society 2020; 68(11):2447–53. Available from: URL: https://www.ncbi.nlm.nih.gov/pubmed/32930389. DOI: 10.1111/jgs.16832.

35. Teesing GR, Graaf M de, Petrignani M, Erasmus V, Klaassen CHW, Schapendonk CME et al. Association of environmental surface contamination with hand hygiene and infections in nursing homes: a prospective cohort study. Infect Prev Pract 2021; 3(2). DOI: 10.1016/j.infpip.2021.100129.

36. Teesing GR, Erasmus V, Nieboer D, Petrignani M, Koopmans MPG, Vos MC et al. Increased hand hygiene compliance in nursing homes after a multimodal intervention: A cluster randomized controlled trial (HANDSOME). Infect Control Hosp Epidemiol 2020; 41(10):1169–77. DOI: 10.1017/ice.2020.319.

37. Teesing GR, Erasmus V, Petrignani M, Koopmans MPG, Graaf M de, Vos MC et al. Improving Hand Hygiene Compliance in Nursing Homes: Protocol for a Cluster Randomized Controlled Trial (HANDSOME Study). JMIR Research Protocols 2020; 9(5):e17419. Available from: URL: https://www.ncbi.nlm.nih.gov/pubmed/32356772. DOI: 10.2196/17419.

38. Allan-Blitz L-T, Aboabdo B, Turner I, Klausner JD. Effect of Frequent SARS-CoV-2 Testing on Weekly Case Rates in Long-Term Care Facilities, Florida, USA. Emerging infectious diseases 2022; 28(9). DOI: 10.3201/eid2809.212577.

39. Ehrlich HY, Harizaj A, Campbell L, Colt M, Yuan K, Rabatsky-Ehr T et al. SARS-CoV-2 in Nursing Homes after 3 Months of Serial, Facilitywide Point Prevalence Testing, Connecticut, USA. Emerging infectious diseases 2021; 27(5):1288–95. Available from: URL: https://www.ncbi.nlm.nih.gov/pubmed/33900171. DOI: 10.3201/eid2705.204936.

40. Telford CT, Onwubiko U, Holland DP, Turner K, Prieto J, Smith S et al. Preventing COVID-19 Outbreaks in Long-Term Care Facilities Through Preemptive Testing of Residents and Staff Members - Fulton County, Georgia, March-May 2020. Morbidity and Mortality Weekly Report 2020:1296–9. Available from: URL: https://www.cdc.gov/mmwr/volumes/69/wr/mm6937a4.htm?s_cid=mm6937a4_w.

1. Downgraded by 2 for risk of bias: inadequate adjustment for confounders in observational studies assessed with ROBINS-I. [↑](#footnote-ref-1)
2. One Study was excluded due to critical concerns of bias. [↑](#footnote-ref-2)
3. Downgraded by 2 for risk of bias: inadequate adjustment for confounders and/or potential contamination due to an interaction with other interventions in observational studies assessed with ROBINS-I. [↑](#footnote-ref-3)
4. 2 Studies were excluded from the analysis due to critical risk of bias [↑](#footnote-ref-4)
5. Downgraded by 2 for risk of bias: inadequate adjustment for confounders and/or potential contamination due to an interaction with other interventions in observational studies assessed with ROBINS-I.
   Downgraded by 1 for imprecision of the direction of effect: confidence intervals allows for the possibility of beneficial, null, or harmful effects. [↑](#footnote-ref-5)
6. 1 Study was excluded from the analysis due to critical risk of bias [↑](#footnote-ref-6)
7. Downgraded by 2 for risk of bias: inadequate adjustment for confounders and/or potential contamination due to an interaction with other interventions in observational studies assessed with ROBINS-I.
   Downgraded by 1 for imprecision of the direction of effect: confidence intervals allows for the possibility of beneficial, null, or harmful effects. [↑](#footnote-ref-7)
8. Downgraded by 1 for risk of bias: potential observer bias and variations in intervention groups suggesting a problem with randomization in cRCT studies assessed with ROB2. [↑](#footnote-ref-8)
9. Downgraded by 2 for risk of bias: inadequate adjustment for confounders in observational studies assessed with ROBINS-I.
   Downgraded by 1 for imprecision of the direction of effect: confidence intervals allows for the possibility of beneficial, null, or harmful effects. [↑](#footnote-ref-9)
10. Downgraded by 2 for risk of bias: inadequate adjustment for confounders in observational studies assessed with ROBINS-I. [↑](#footnote-ref-10)
11. 2 Studies were excluded from the analysis due to critical risk of bias [↑](#footnote-ref-11)
12. 1 Study was excluded from the analysis due to critical risk of bias [↑](#footnote-ref-12)
13. Downgraded by 2 for risk of bias: inadequate adjustment for confounders and/or potential contamination due to an interaction with other interventions in observational studies assessed with ROBINS-I.
    Downgraded by 1 for imprecision of the direction of effect: confidence intervals allow for the possibility of beneficial, null, or harmful effects. [↑](#footnote-ref-13)
14. Downgraded by 1 for risk of bias: inadequate adjustment for confounders in observational studies assessed with ROBINS-I. [↑](#footnote-ref-14)
15. Downgraded by 2 for risk of bias: inadequate adjustment for confounders in observational studies assessed with ROBINS-I.

    Downgraded by 1 for imprecision of the direction of effect: confidence intervals allow for the possibility of beneficial, null, or harmful effects. [↑](#footnote-ref-15)
